# Supplementary material for: Transcriptomics and Metabolomics Reveal the Critical Genes of Carotenoid Biosynthesis and Color Formation of Goji (Lycium barbarum L.) Fruit Ripening
Source: Plants (Basel). 2023 Jul 27;12(15):2791. doi: 10.3390/plants12152791 (PMC10421014; doi:10.3390/plants12152791)
Supplement: Supplementary file 1 [file plants-12-02791-s001.zip › Figure S1.pdf]

Compound name: α-Carotene
Regression Equation: y = 0.35893 x + 0.00122 (r = 0.99842) (weighting: 1 / x)

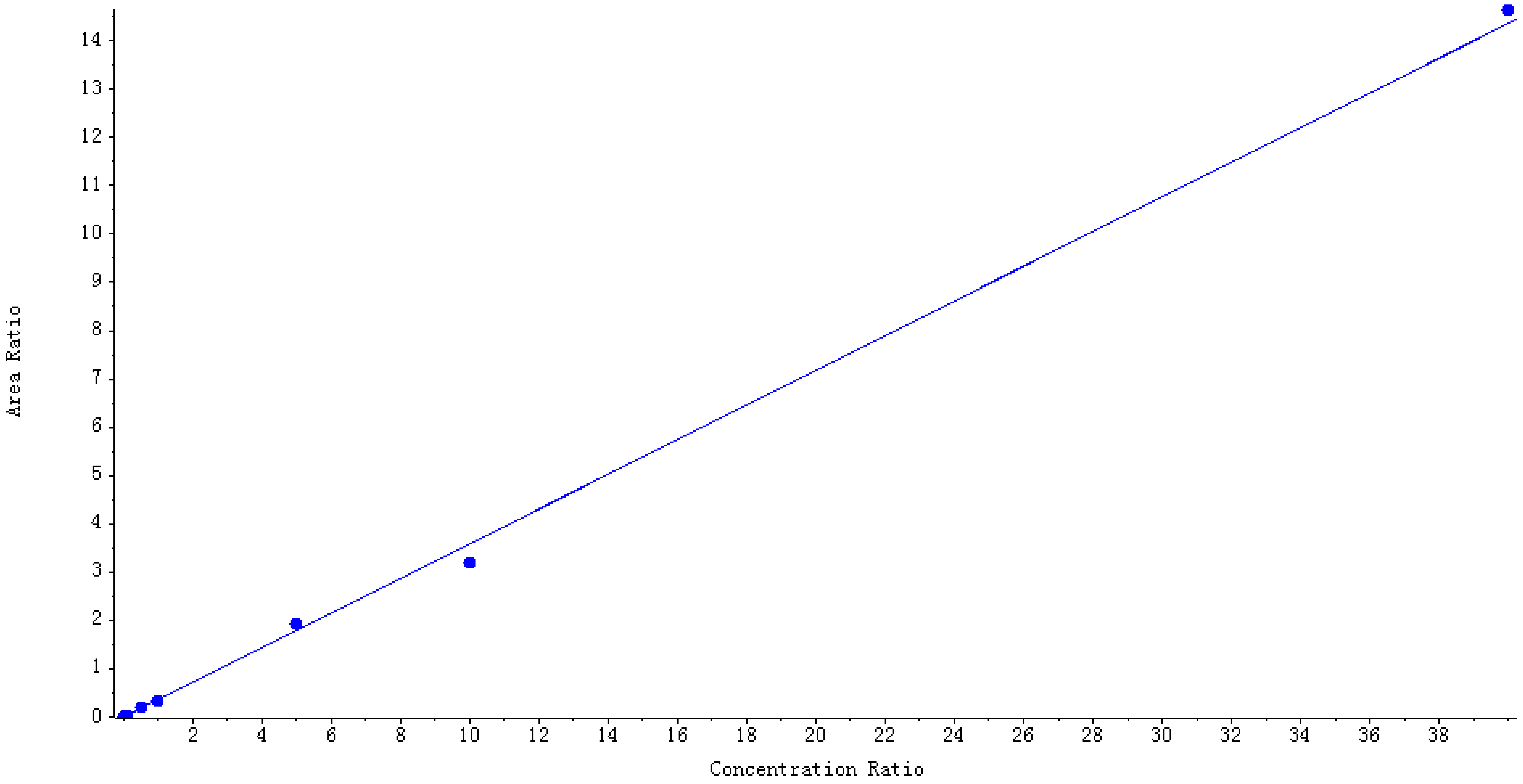

Peak Review

BLANK
C01 AREA:N/A S/N:N/A

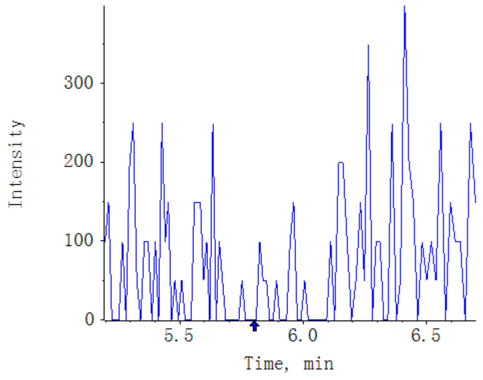

MWMS_20200904_1
C01 AREA:6.568e5 S/N:116.3

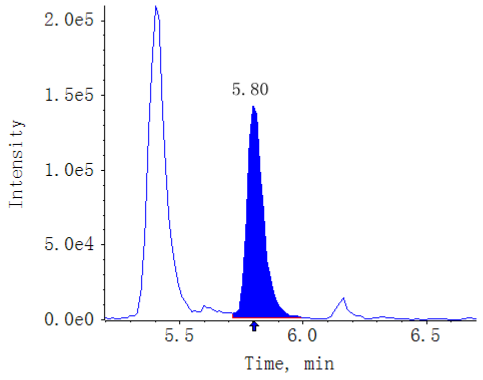

A20024797a_a
C01 AREA:6.225e4 S/N:6.2

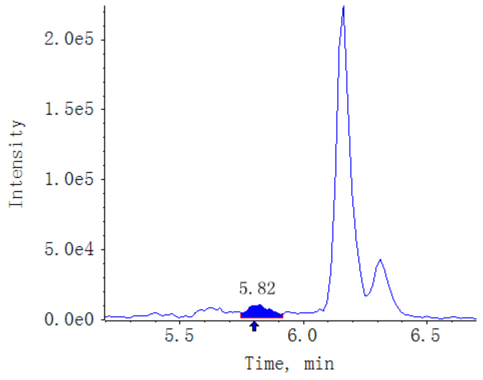

A20024797a_b
C01 AREA:6.973e4 S/N:5.4

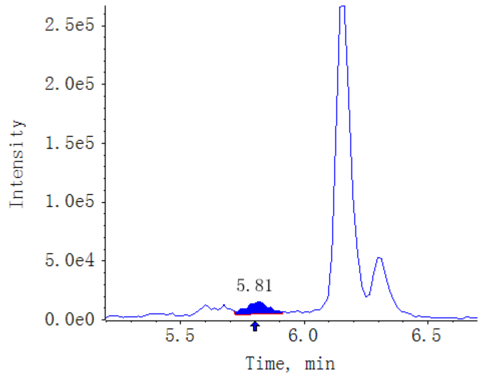

A20024800a_a
C01 AREA:N/A S/N:N/A

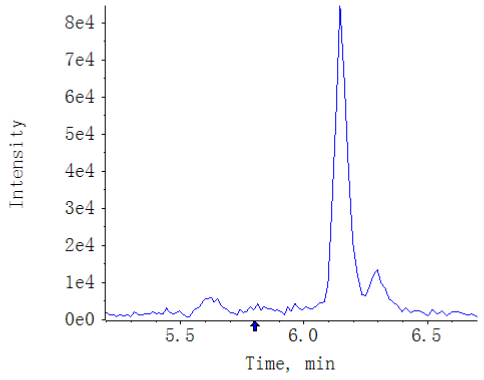

A20024800a_b
C01 AREA:N/A S/N:N/A

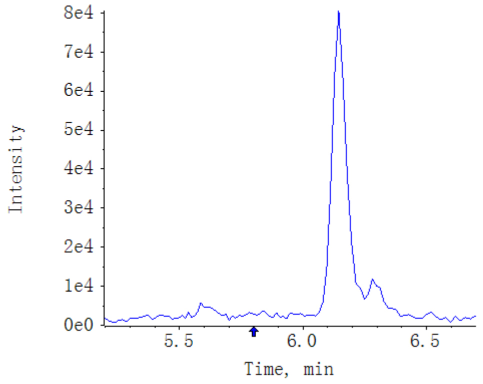

A20024802a_a
C01 AREA:N/A S/N:N/A

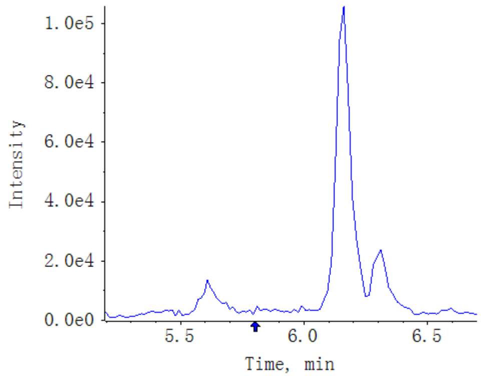

A20024802a_b
C01 AREA:N/A S/N:N/A

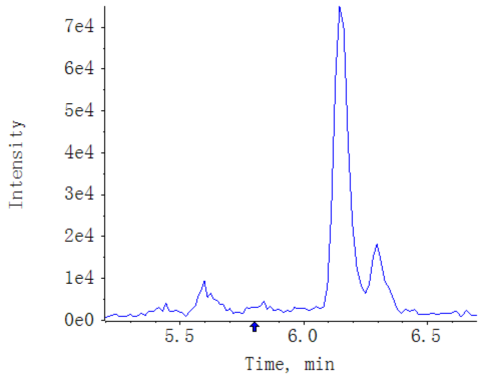

A20024805a_a
C01 AREA:5.760e4 S/N:5.0

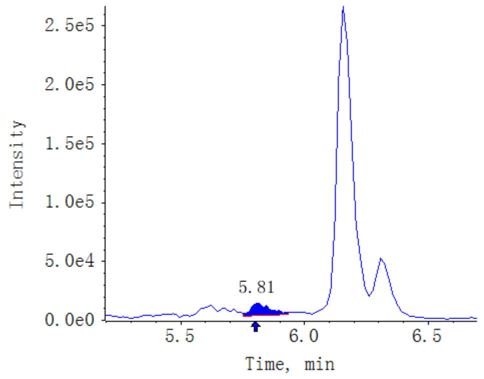

A20024805a_b
C01 AREA:6.152e4 S/N:4.6

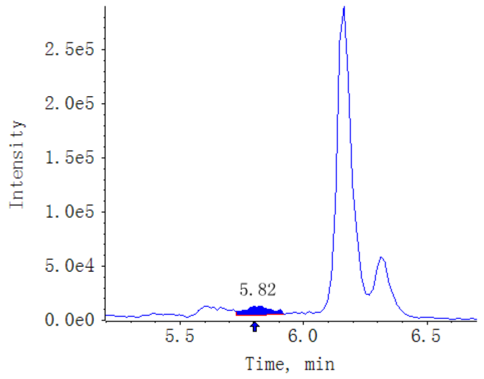

A20024808a_a
C01 AREA:N/A S/N:N/A

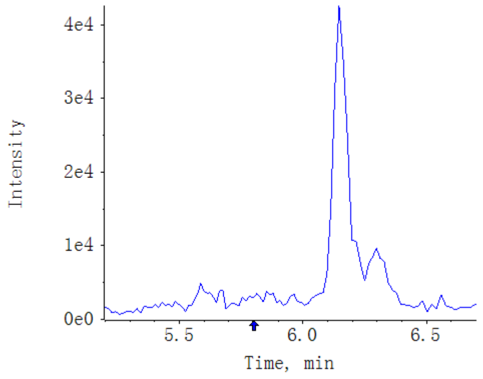

A20024808a_b
C01 AREA:N/A S/N:N/A

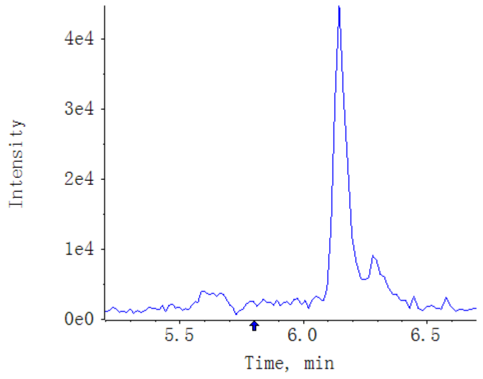

A20024811a_a
C01 AREA:N/A S/N:N/A

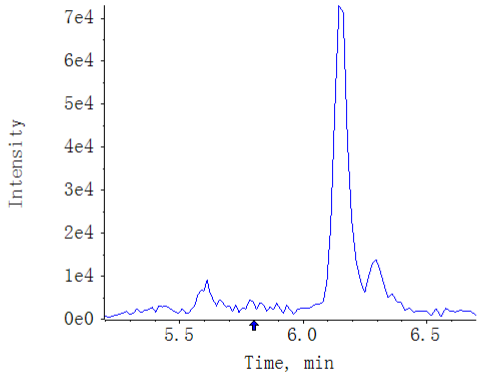

A20024811a_b
C01 AREA:N/A S/N:N/A

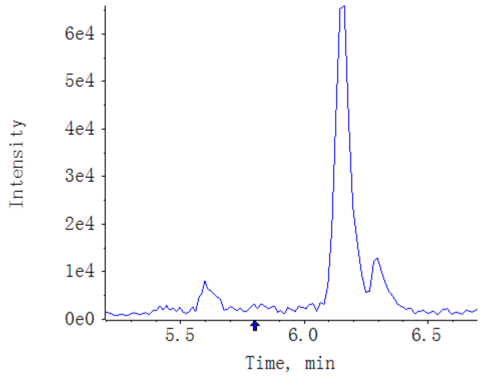

Compound name: Antheraxanthin
Regression Equation: y = 1.12432 x + -0.24196 (r = 0.99740) (weighting: 1 / x)

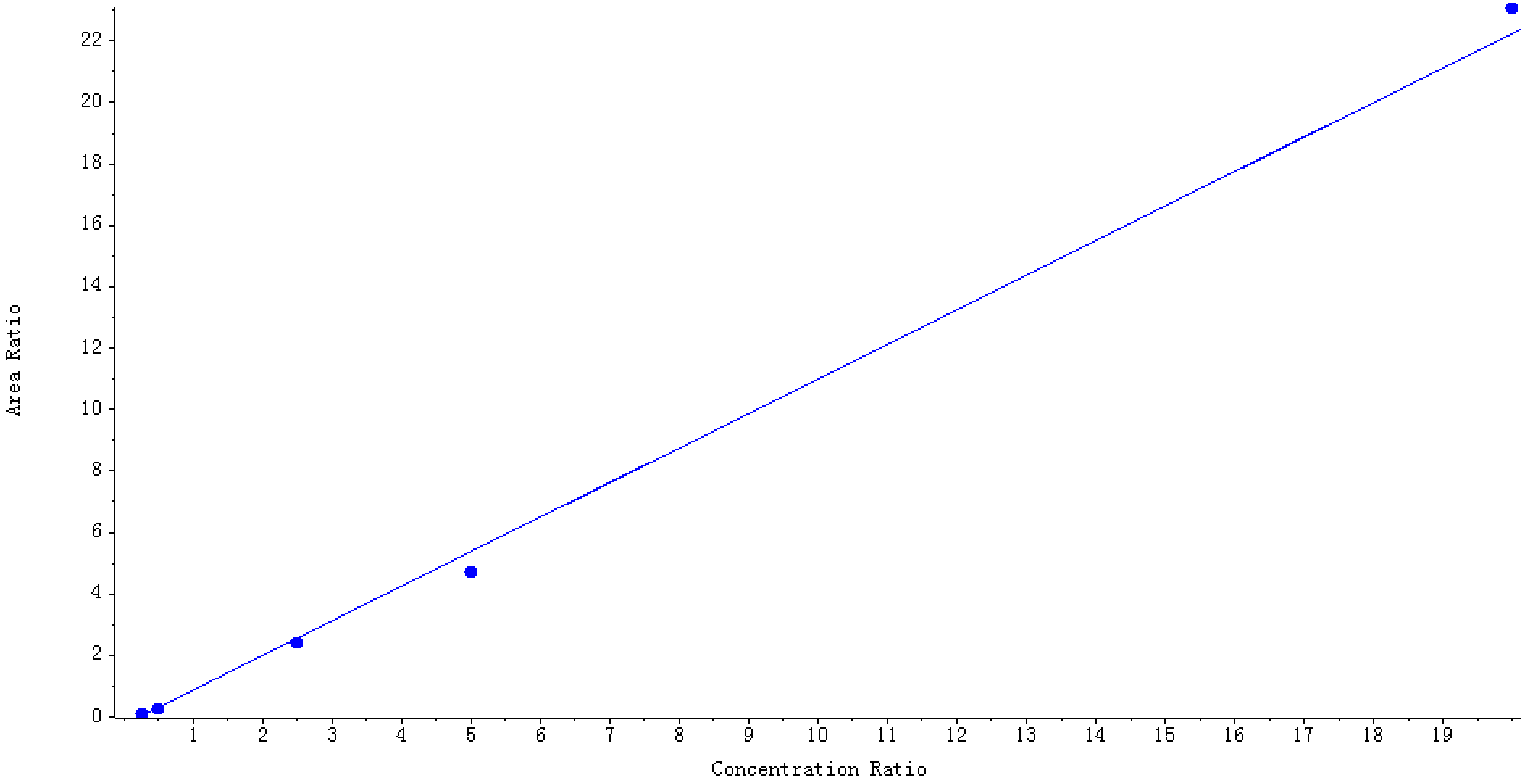

Peak Review

BLANK
C02 AREA:N/A S/N:N/A

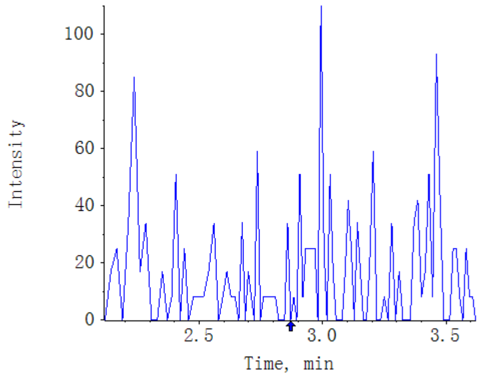

MWMS_20200904_1
C02 AREA:1.114e6 S/N:117.7

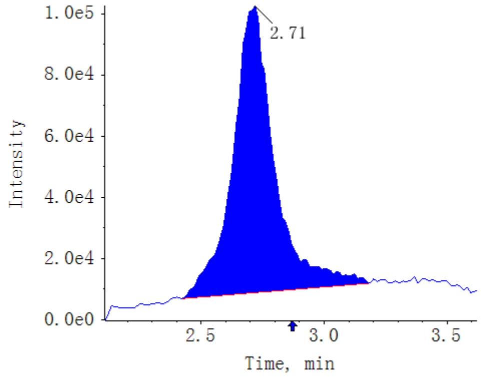

A20024797a_a
C02 AREA:4.363e5 S/N:49.7

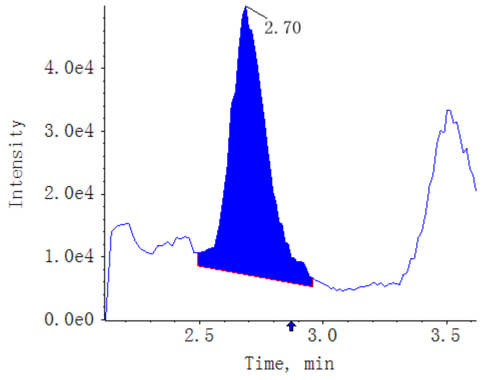

A20024797a_b
C02 AREA:4.631e5 S/N:50.9

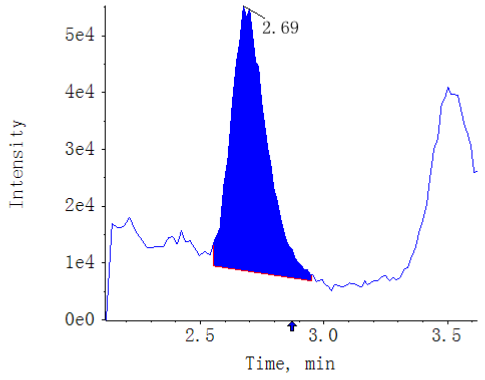

A20024800a_a
C02 AREA:5.865e5 S/N:53.4

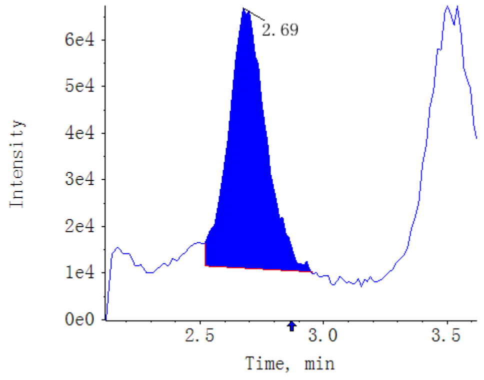

A20024800a_b
C02 AREA:6.705e5 S/N:57.6

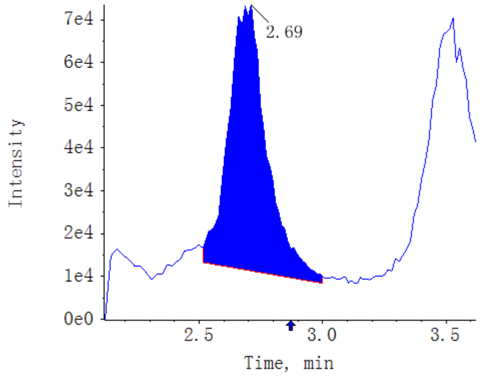

A20024802a_a
C02 AREA:2.862e4 S/N:16.0

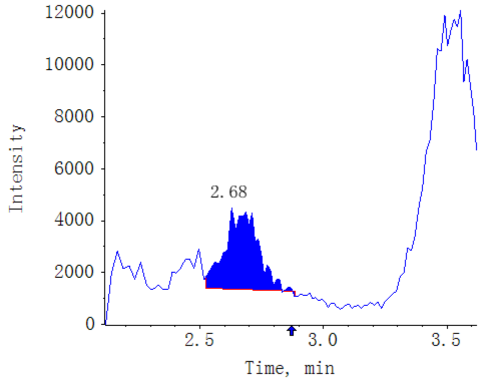

A20024802a_b
C02 AREA:2.796e4 S/N:18.6

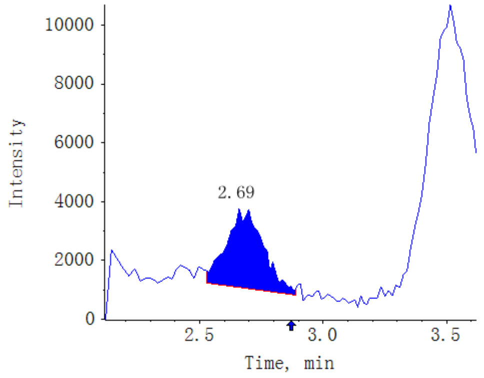

A20024805a_a
C02 AREA:3.967e5 S/N:39.2

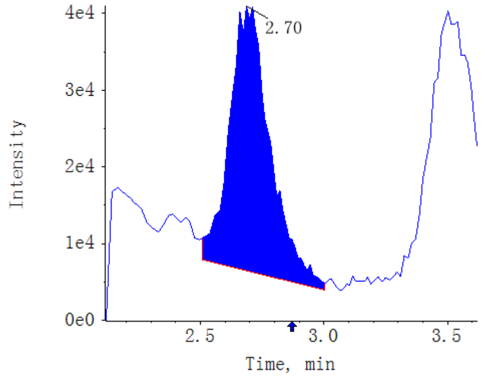

A20024805a_b
C02 AREA:3.408e5 S/N:40.8

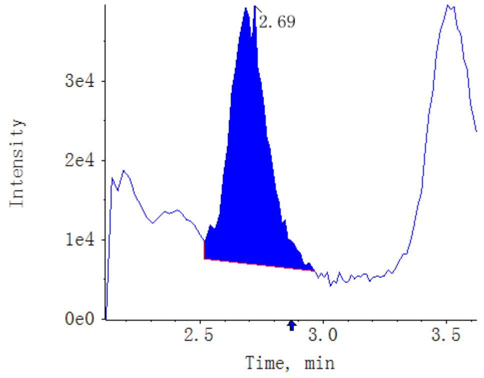

A20024808a_a
C02 AREA:1.544e6 S/N:62.6

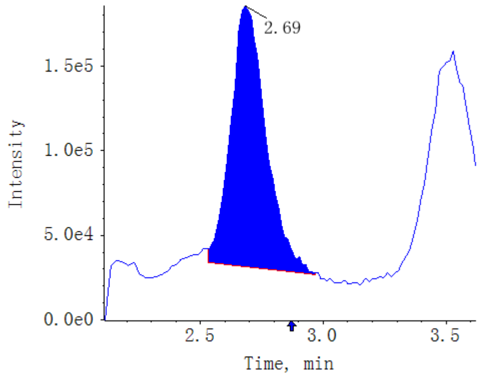

A20024808a_b
C02 AREA:1.809e6 S/N:73.0

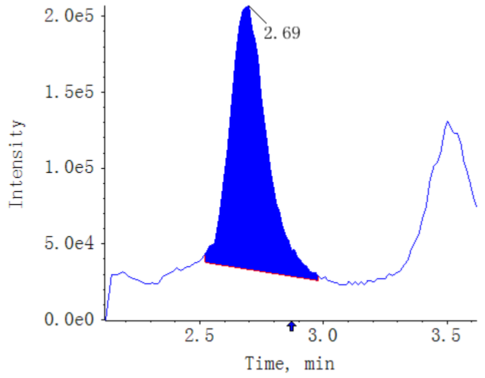

A20024811a_a
C02 AREA:1.368e6 S/N:90.4

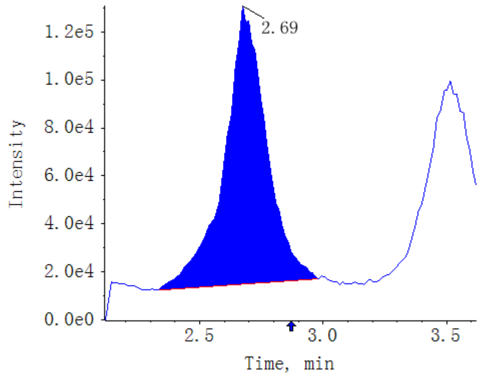

A20024811a_b
C02 AREA:1.196e6 S/N:75.9

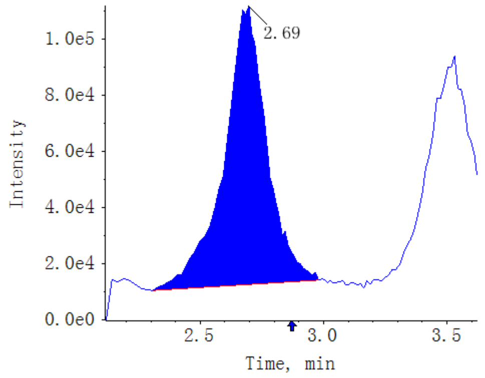

Compound name: Lycopene

Regression Equation: y = 0.16141 x + 8.27726e-4 (r = 0.99938) (weighting: 1 / x)

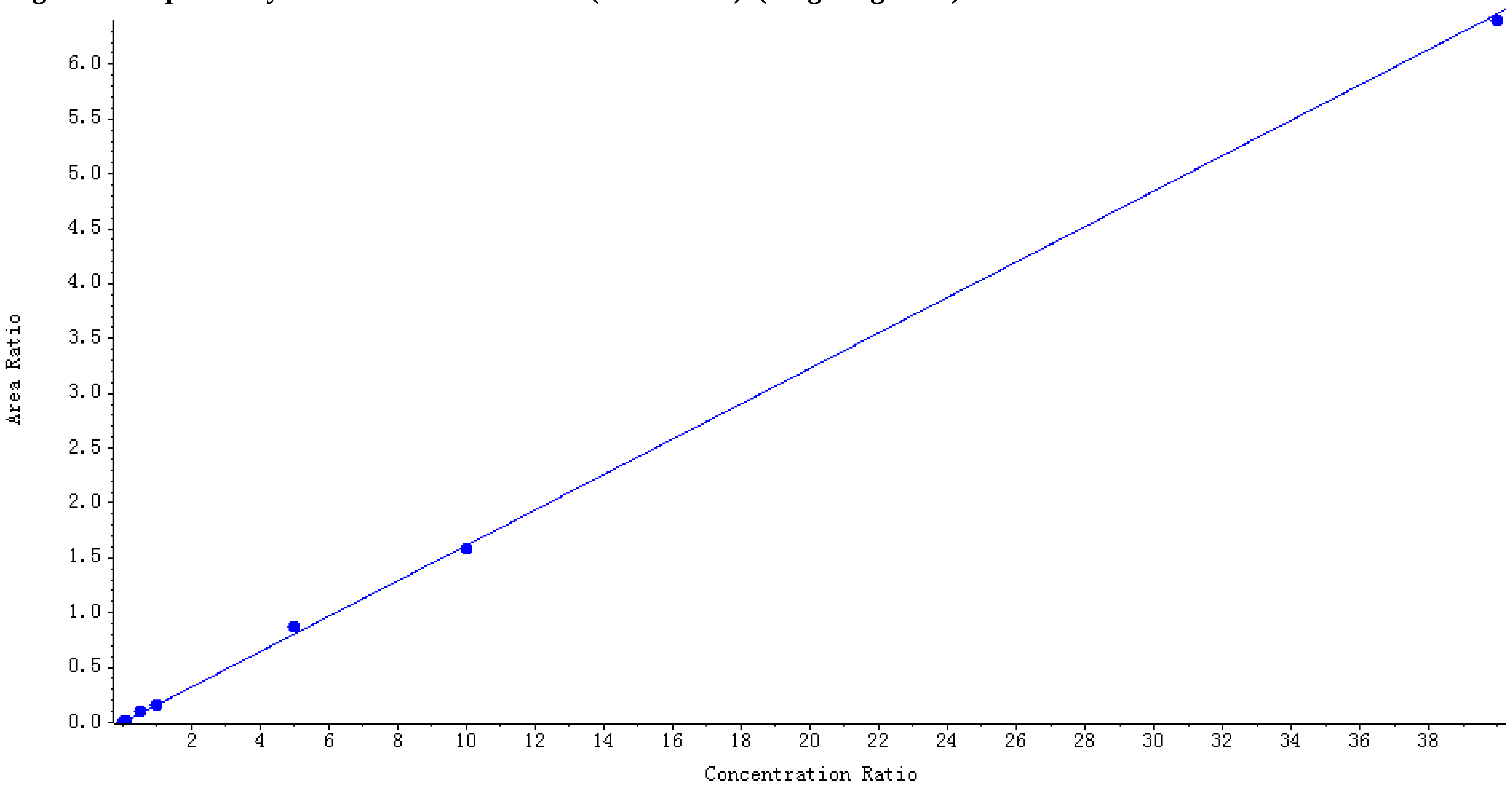

Peak Review

BLANK
C03 AREA:N/A S/N:N/A

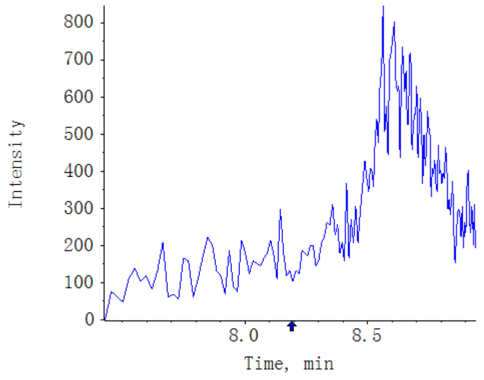

MWMS_20200904_1
C03 AREA:2.879e5 S/N:520.1

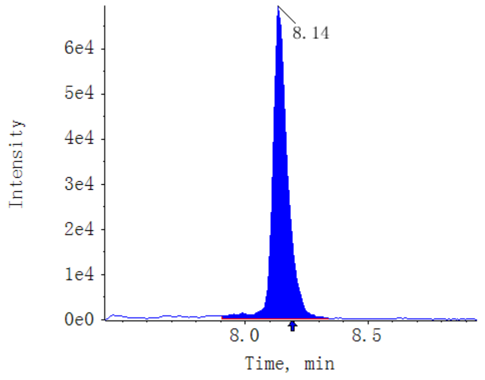

A20024797a_a
C03 AREA:N/A S/N:N/A

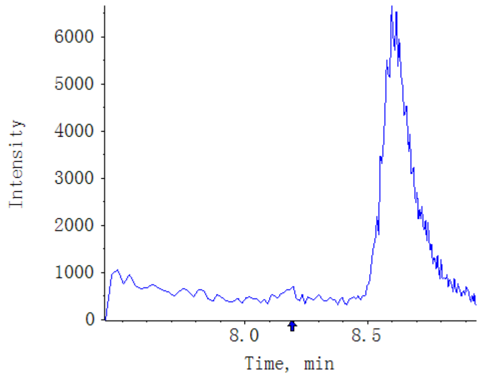

A20024797a_b
C03 AREA:N/A S/N:N/A

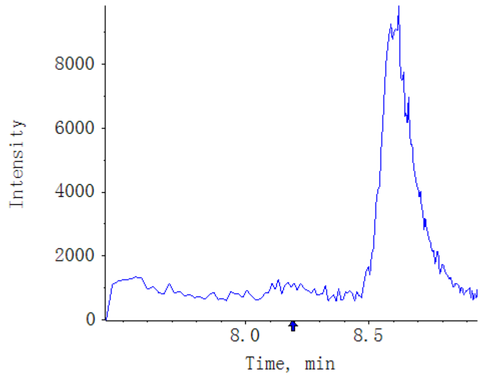

A20024800a_a
C03 AREA:N/A S/N:N/A

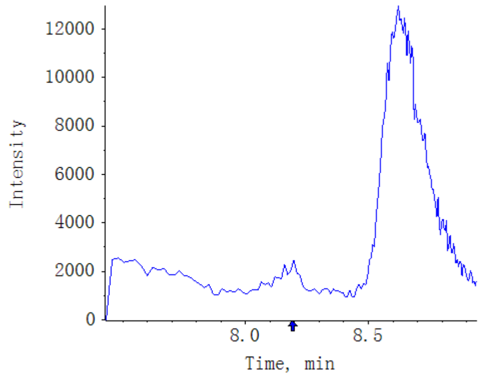

A20024800a_b
C03 AREA:N/A S/N:N/A

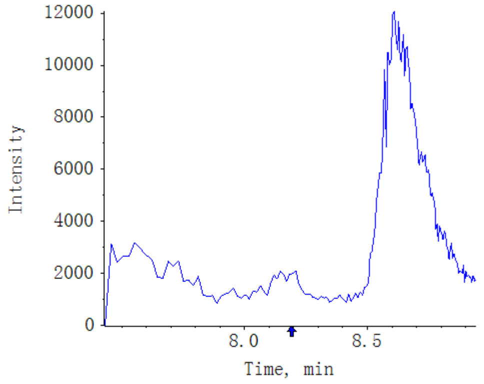

A20024802a_a
C03 AREA:1.366e4 S/N:9.3

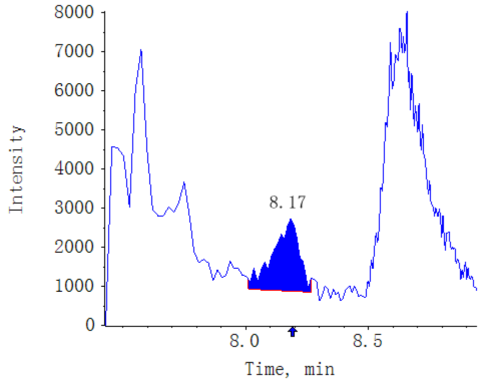

A20024802a_b
C03 AREA:1.003e4 S/N:10.3

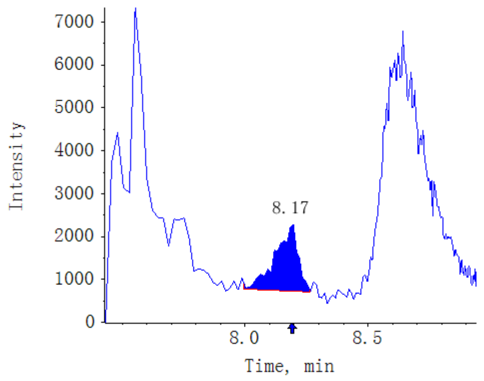

A20024805a_a
C03 AREA:N/A S/N:N/A

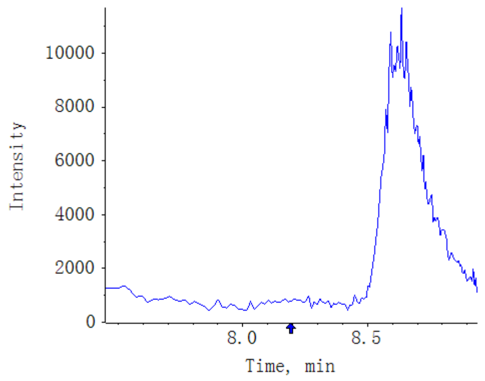

A20024805a_b
C03 AREA:N/A S/N:N/A

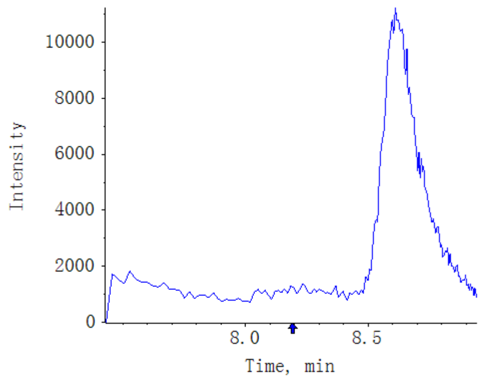

A20024808a_a
C03 AREA:N/A S/N:N/A

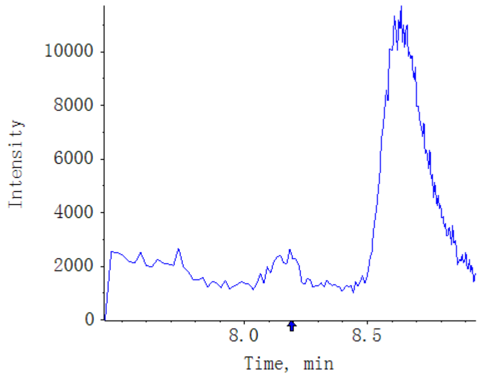

A20024808a_b
C03 AREA:N/A S/N:N/A

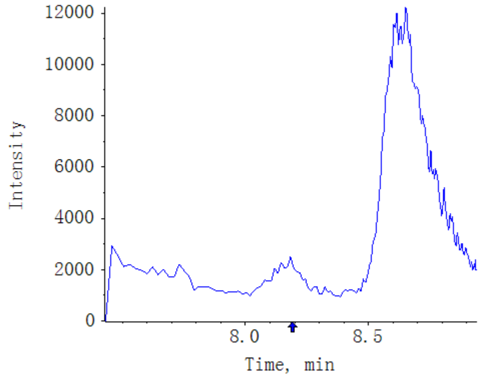

A20024811a_a
C03 AREA:3.403e4 S/N:24.8

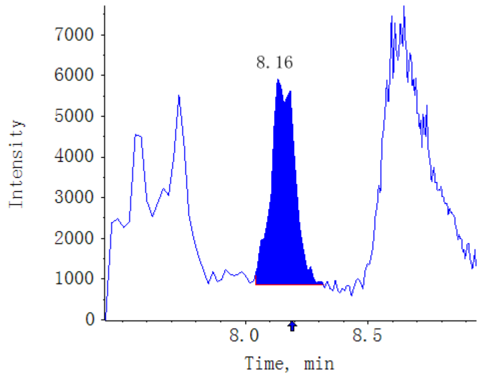

A20024811a_b
C03 AREA:2.853e4 S/N:20.5

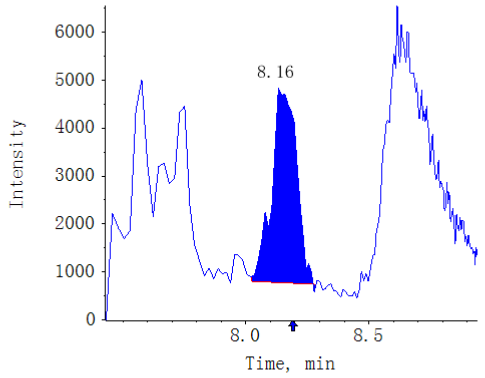

Compound name: Zeaxanthin

Regression Equation: y = 0.19627 x + 0.00647 (r = 0.99741) (weighting: 1 / x)

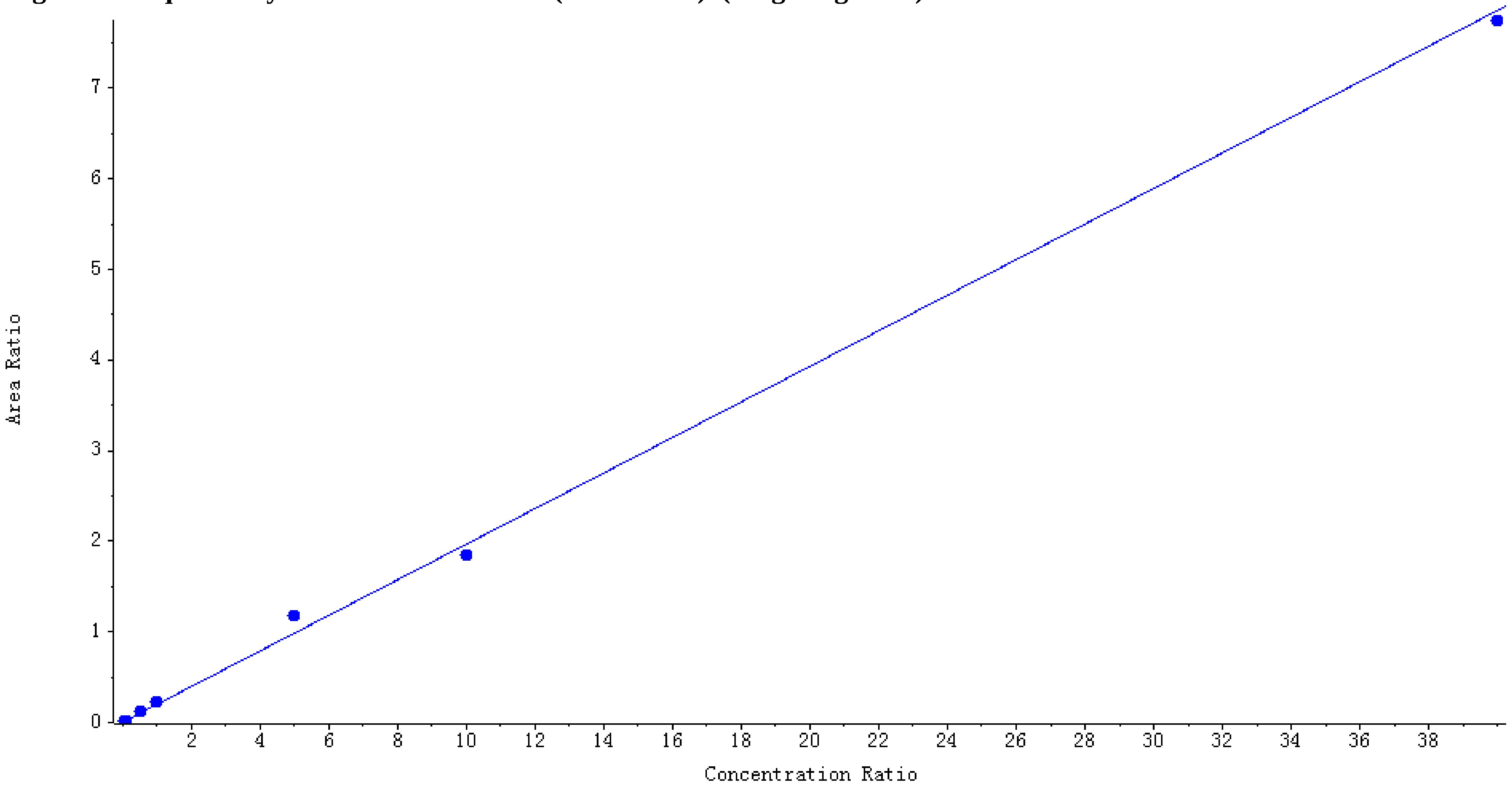

Peak Review

BLANK
C04 AREA:N/A S/N:N/A

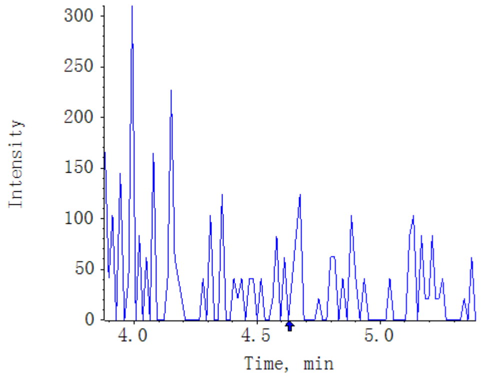

MWMS_20200904_1
C04 AREA:3.929e5 S/N:200.6

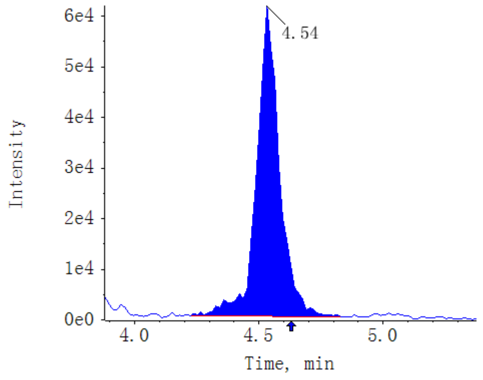

A20024797a_a
C04 AREA:3.897e5 S/N:86.2

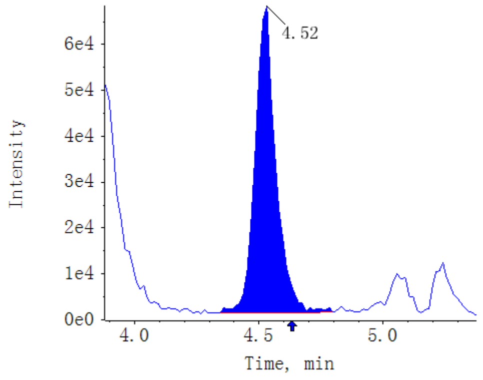

A20024797a_b
C04 AREA:4.373e5 S/N:93.3

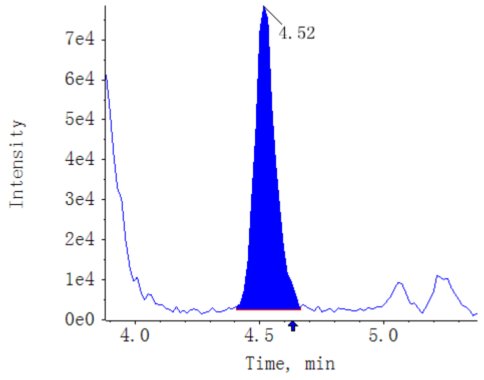

A20024800a_a
C04 AREA:5.242e6 S/N:354.8

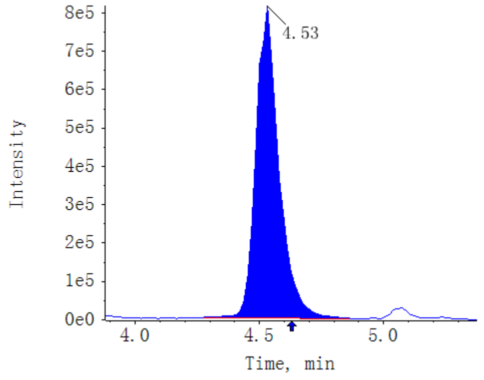

A20024800a_b
C04 AREA:5.592e6 S/N:357.3

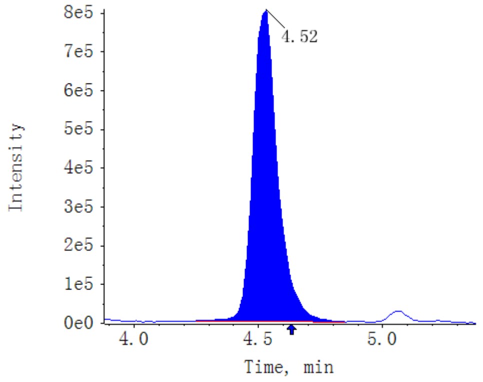

A20024802a_a
C04 AREA:1.952e6 S/N:338.1

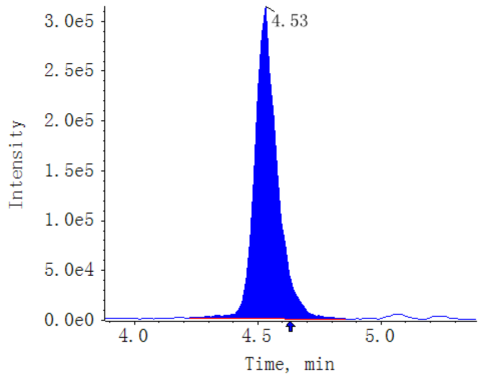

A20024802a_b
C04 AREA:1.255e6 S/N:287.4

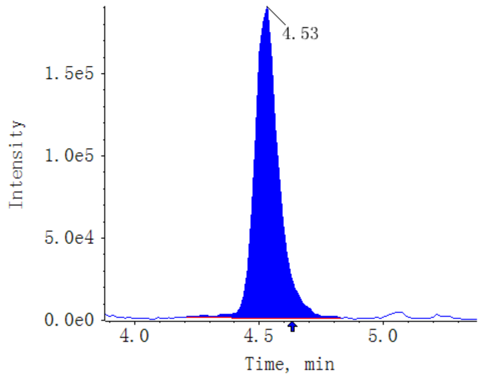

A20024805a_a
C04 AREA:4.229e5 S/N:72.2

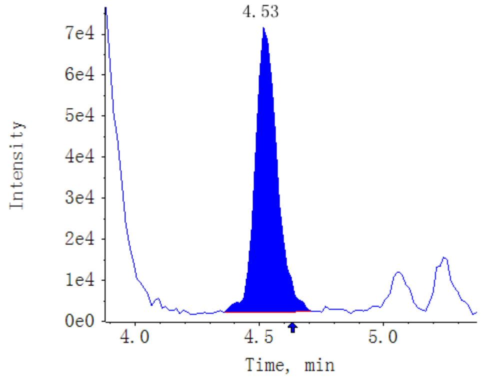

A20024805a_b
C04 AREA:4.573e5 S/N:76.1

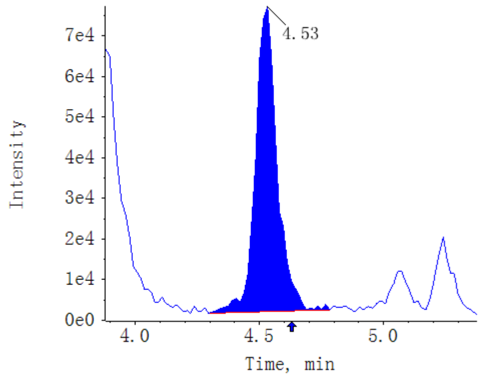

A20024808a_a
C04 AREA:1.271e7 S/N:391.3

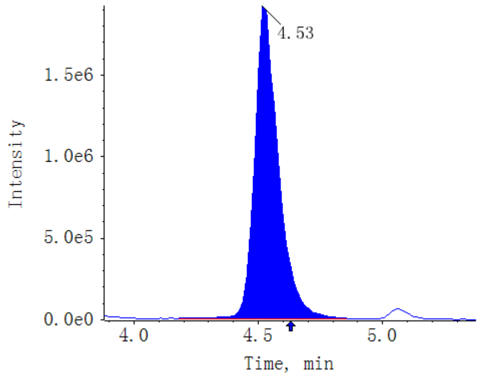

A20024808a_b
C04 AREA:1.161e7 S/N:421.3

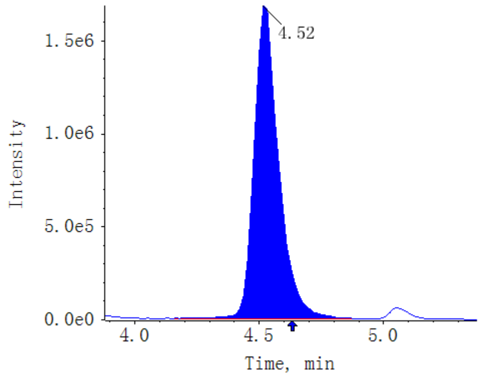

A20024811a_a
C04 AREA:1.131e7 S/N:463.8

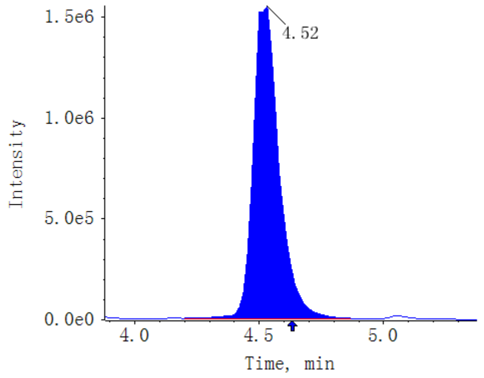

A20024811a_b
C04 AREA:1.054e7 S/N:452.1

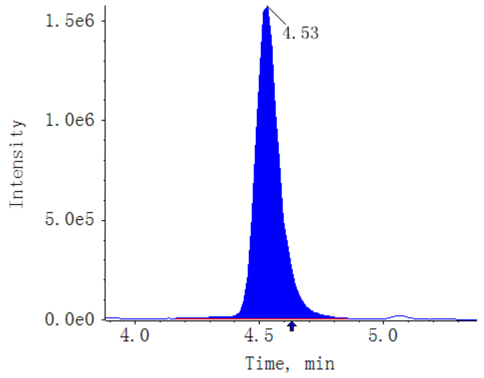

Compound name: Violaxanthin

Regression Equation: y = 3.55083 x + 9.23931e-4 (r = 0.99411) (weighting: 1 / x)

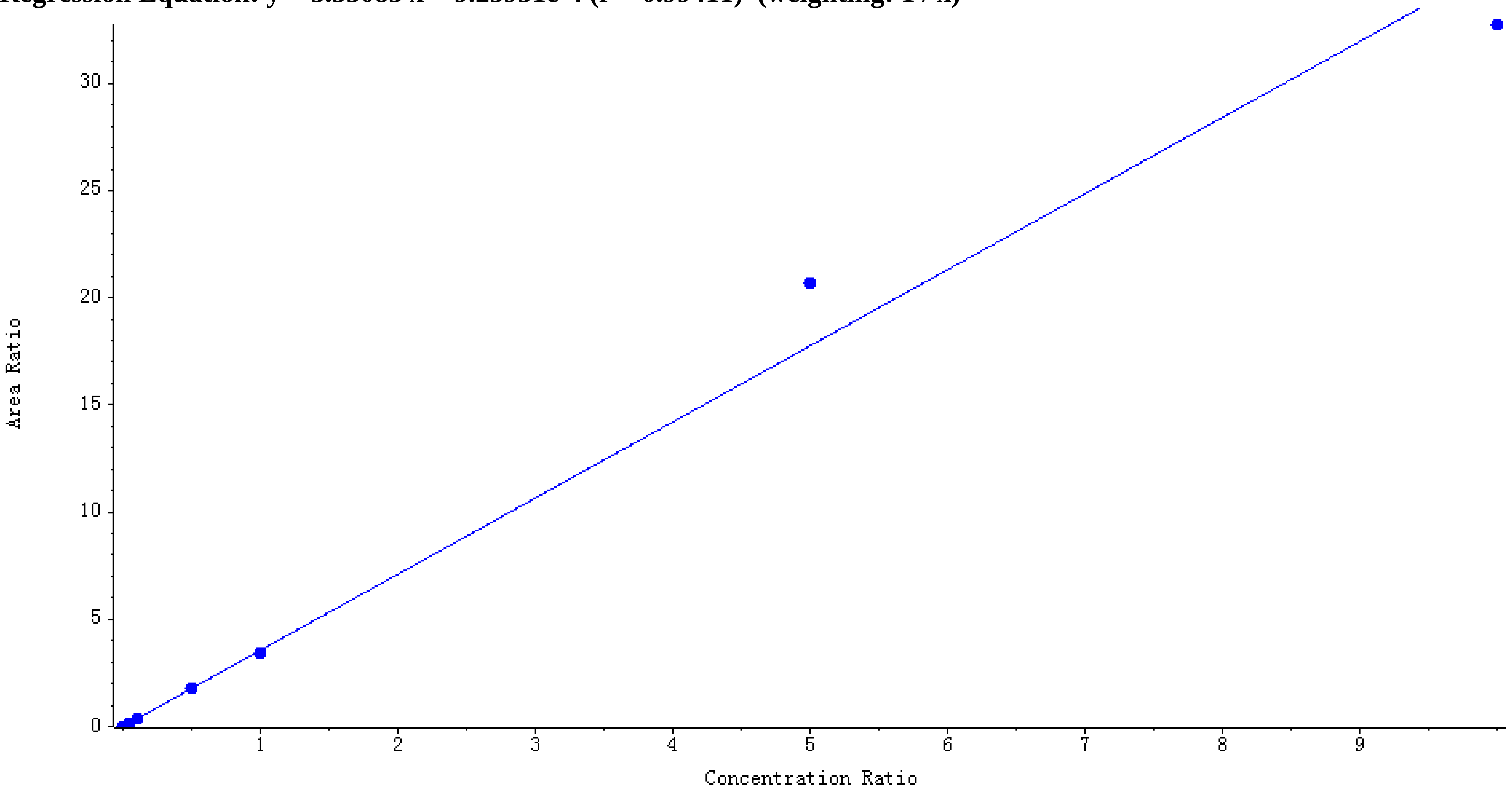

Peak Review

BLANK
C05 AREA:N/A S/N:N/A

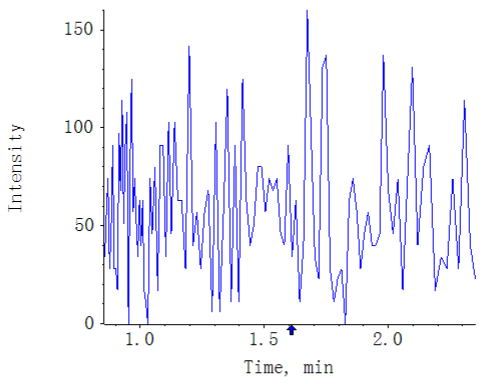

MWMS_20200904_1
C05 AREA:3.279e5 S/N:21.8

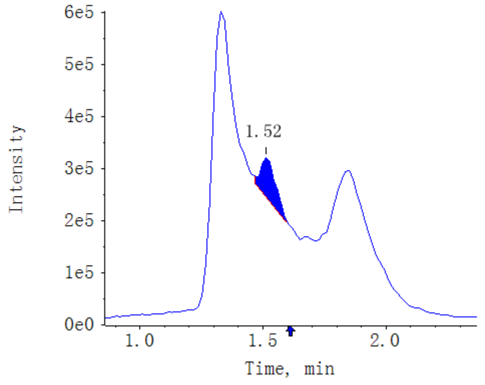

A20024797a_a
C05 AREA:3.276e6 S/N:15.0

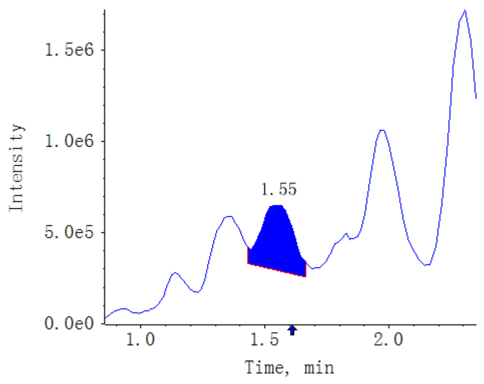

A20024797a_b
C05 AREA:2.626e6 S/N:13.6

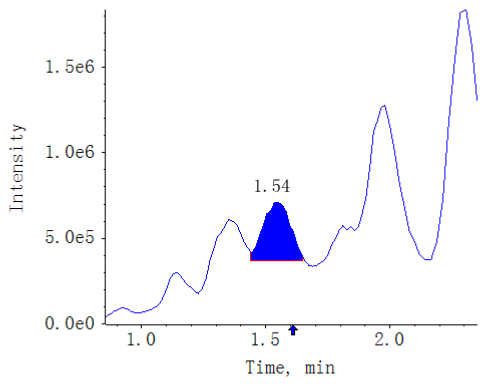

A20024800a_a
C05 AREA:3.763e5 S/N:12.8

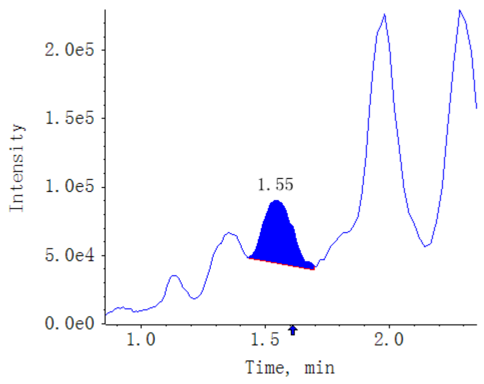

A20024800a_b
C05 AREA:3.756e5 S/N:13.3

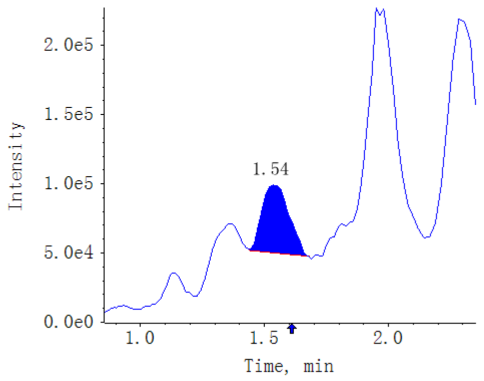

A20024802a_a
C05 AREA:6.818e4 S/N:16.5

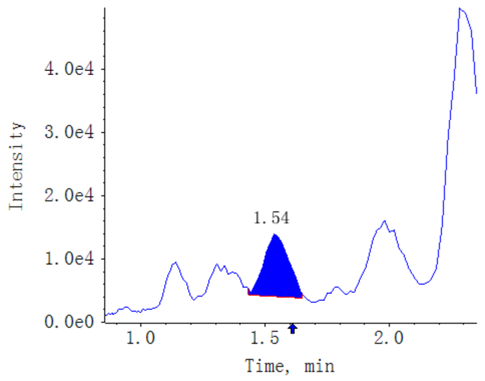

A20024802a_b
C05 AREA:6.355e4 S/N:15.9

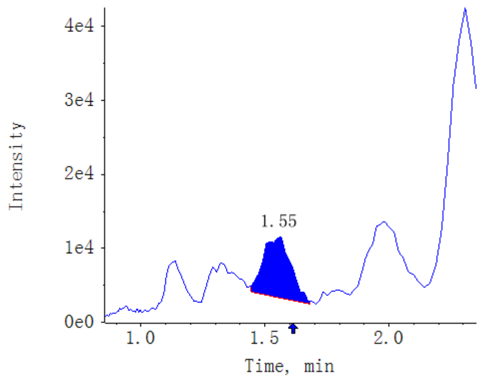

A20024805a_a
C05 AREA:2.539e6 S/N:12.7

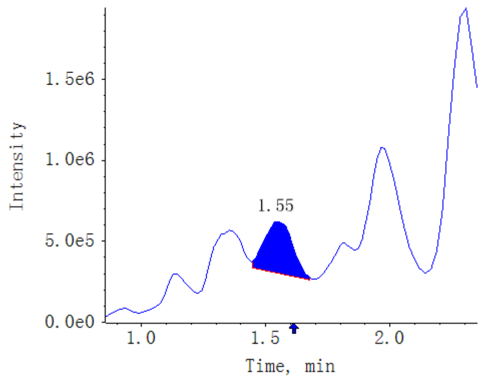

A20024805a_b
C05 AREA:2.441e6 S/N:11.8

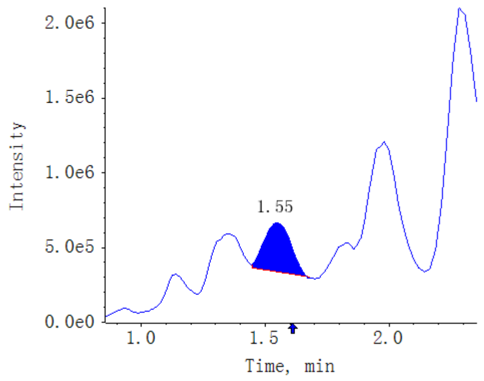

A20024808a_a
C05 AREA:4.809e5 S/N:13.9

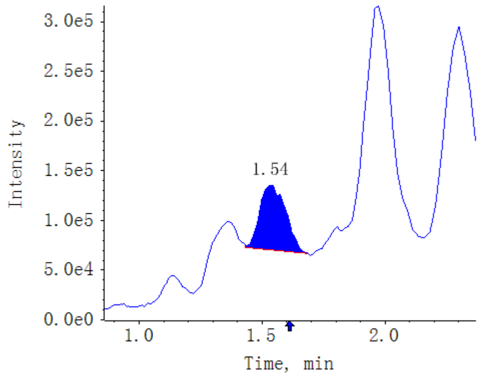

A20024808a_b
C05 AREA:5.632e5 S/N:17.8

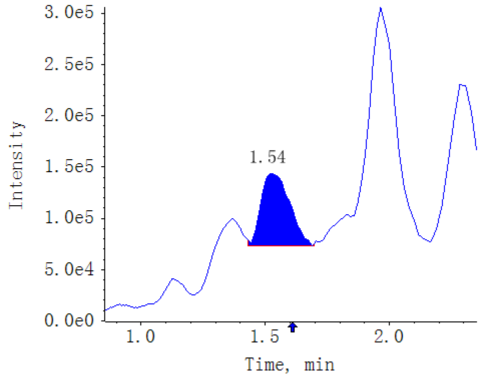

A20024811a_a
C05 AREA:1.850e5 S/N:12.6

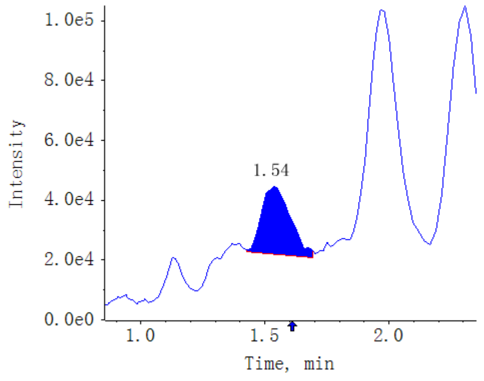

A20024811a_b
C05 AREA:1.825e5 S/N:14.3

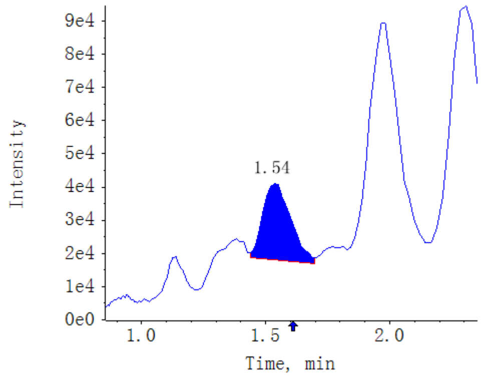

Compound name: γ-Carotene
Regression Equation: y = 0.51678 x + -0.01709 (r = 0.99830) (weighting: 1 / x)

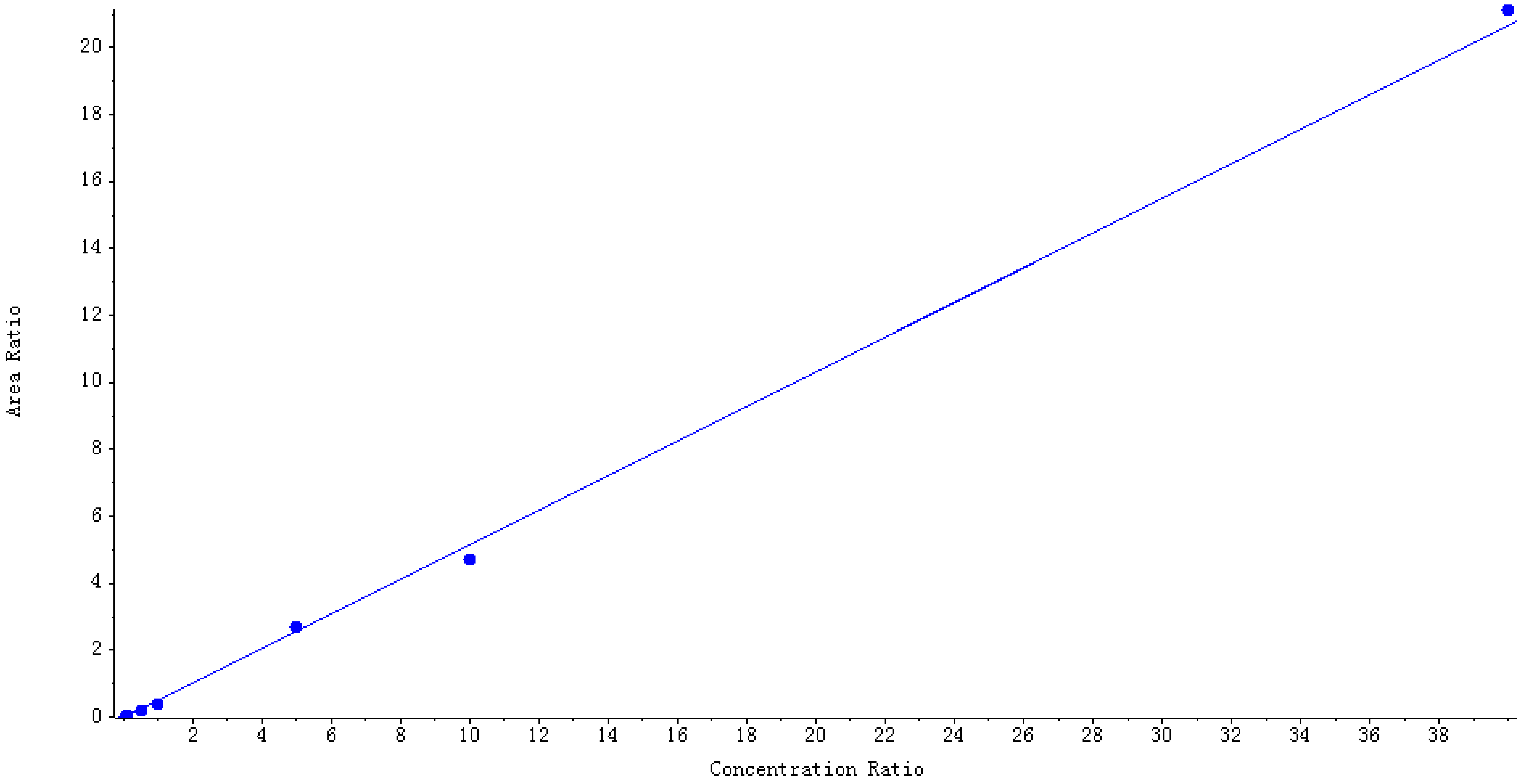

Peak Review

BLANK
C06 AREA:N/A S/N:N/A

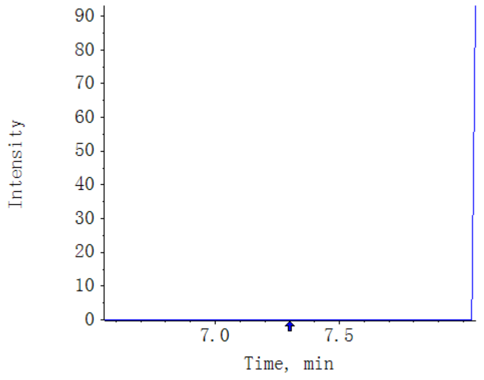

MWMS_20200904_1
C06 AREA:5.281e5 S/N:165.3

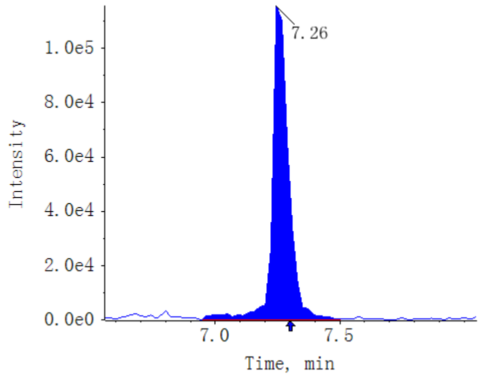

A20024797a_a
C06 AREA:N/A S/N:N/A

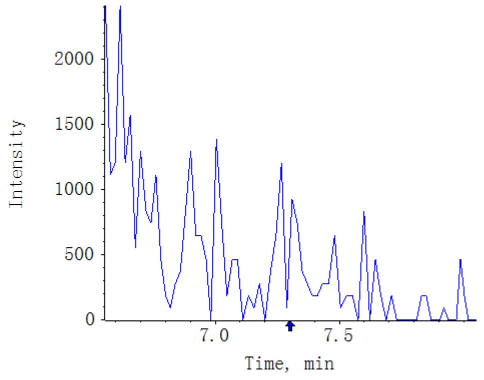

A20024797a_b
C06 AREA:N/A S/N:N/A

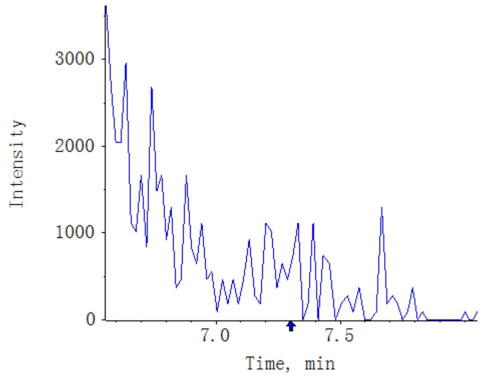

A20024800a_a
C06 AREA:1.870e4 S/N:16.2

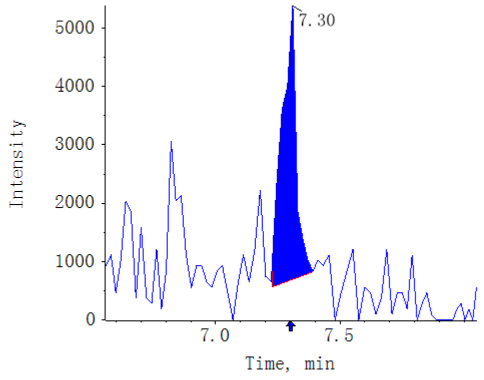

A20024800a_b
C06 AREA:2.704e4 S/N:21.8

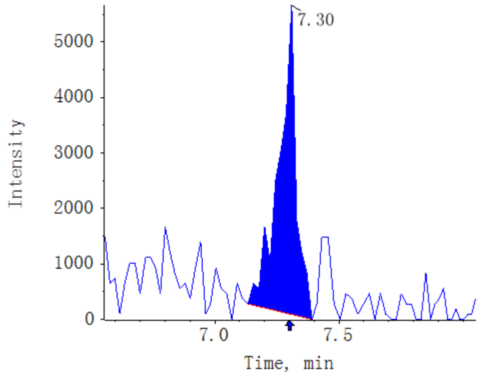

A20024802a_a
C06 AREA:9.137e4 S/N:28.0

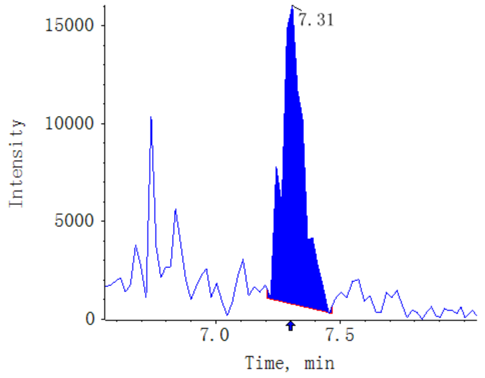

A20024802a_b
C06 AREA:7.250e4 S/N:38.2

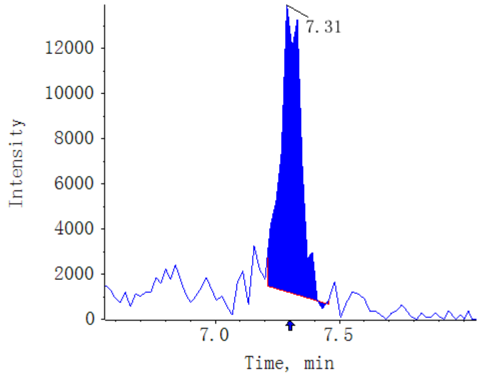

A20024805a_a
C06 AREA:N/A S/N:N/A

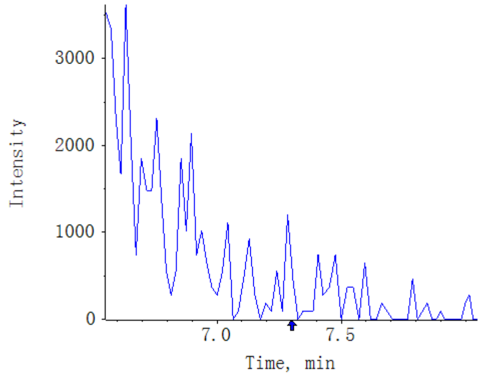

A20024805a_b
C06 AREA:N/A S/N:N/A

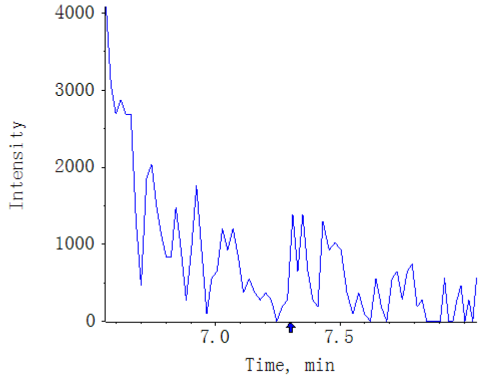

A20024808a_a
C06 AREA:1.570e4 S/N:11.2

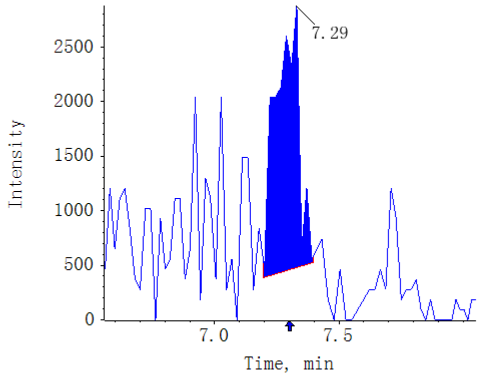

A20024808a_b
C06 AREA:1.276e4 S/N:27.8

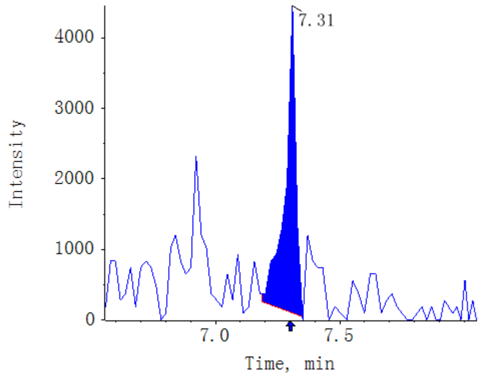

A20024811a_a
C06 AREA:8.199e4 S/N:46.3

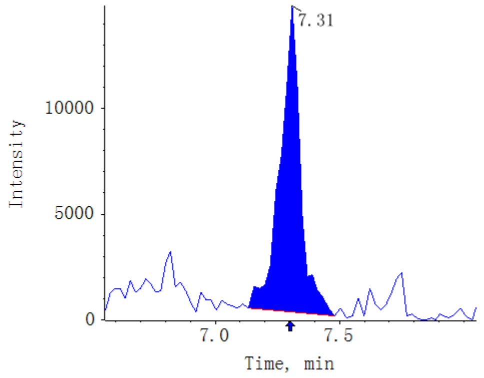

A20024811a_b
C06 AREA:7.030e4 S/N:21.2

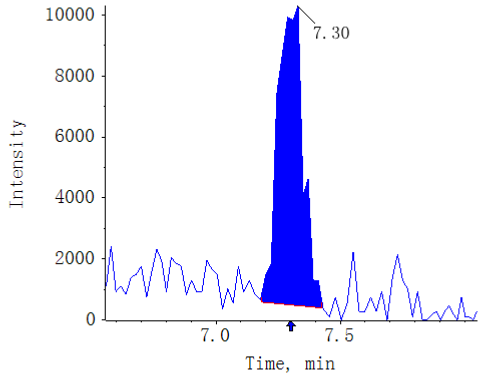

Compound name: Neoxanthin
Regression Equation: y = 2.48212 x + -0.00832 (r = 0.99810) (weighting: 1 / x)

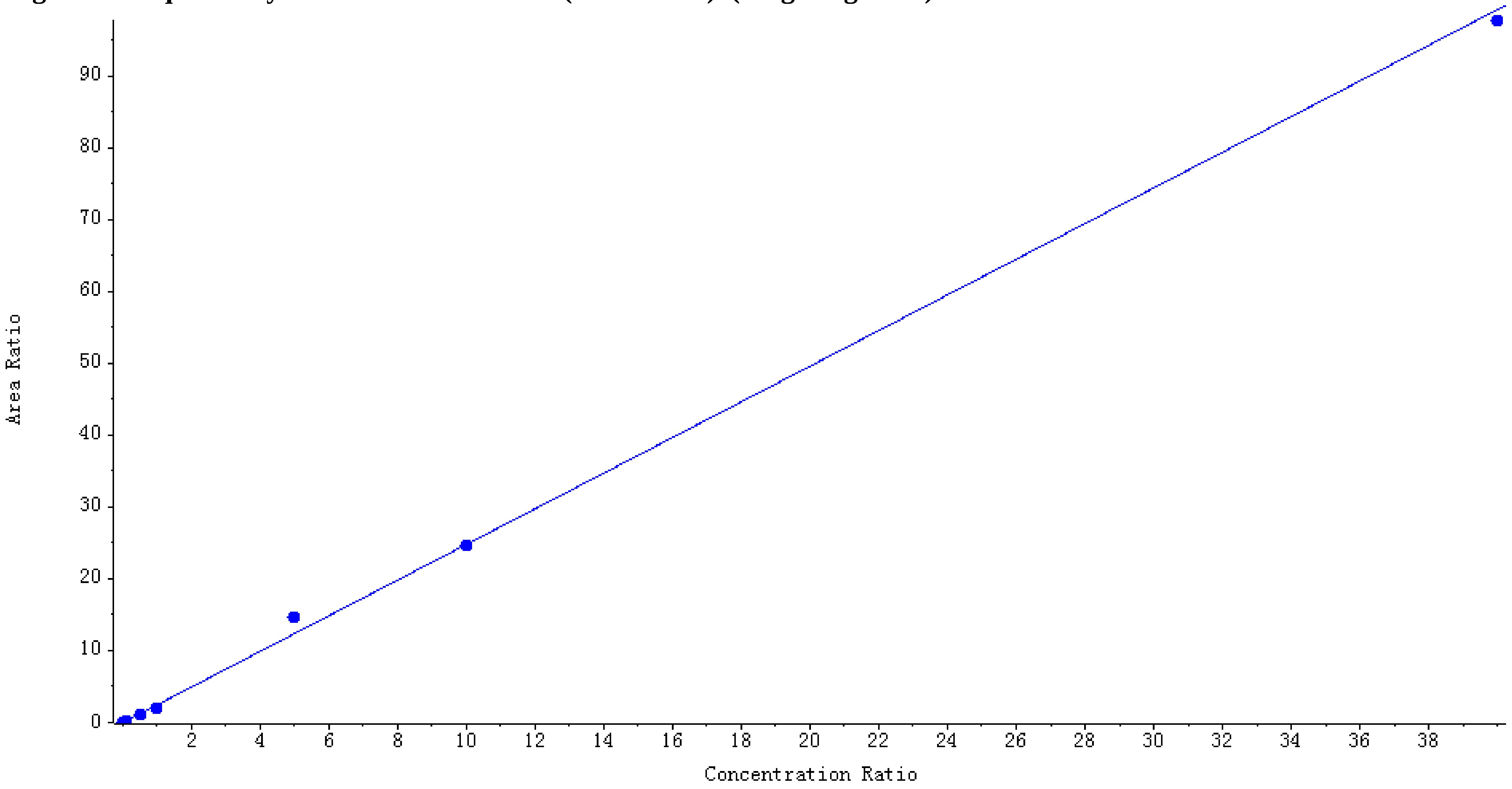

Peak Review

BLANK
C07 AREA:N/A S/N:N/A

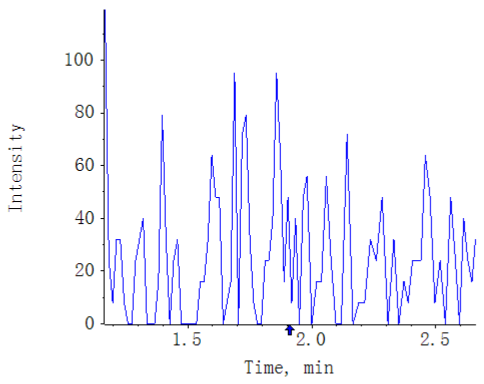

MWMS_20200904_1
C07 AREA:4.597e6 S/N:154.0

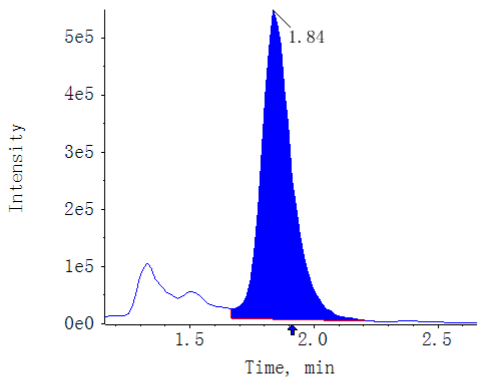

A20024797a_a
C07 AREA:7.368e6 S/N:81.0

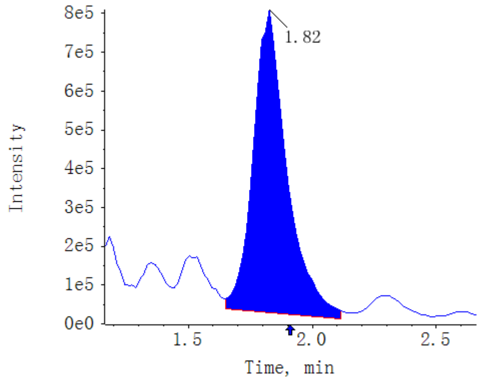

A20024797a_b
C07 AREA:8.412e6 S/N:85.4

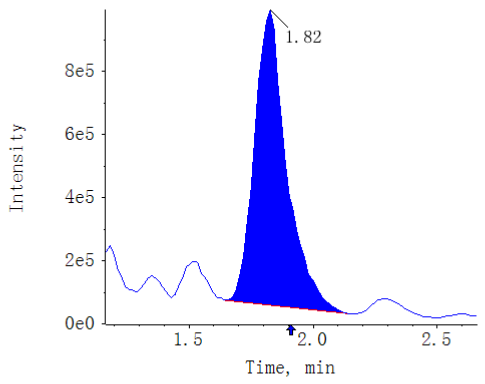

A20024800a_a
C07 AREA:8.183e5 S/N:74.9

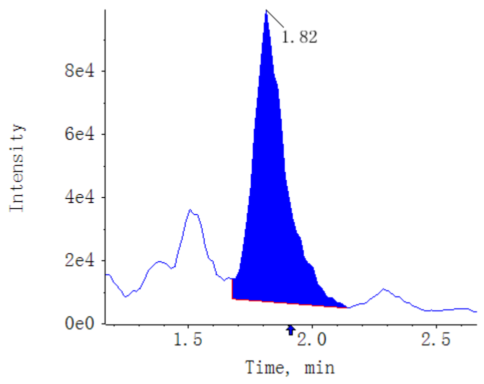

A20024800a_b
C07 AREA:8.364e5 S/N:61.5

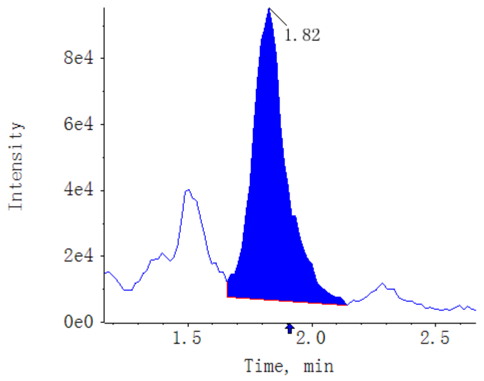

A20024802a_a
C07 AREA:6.127e4 S/N:25.9

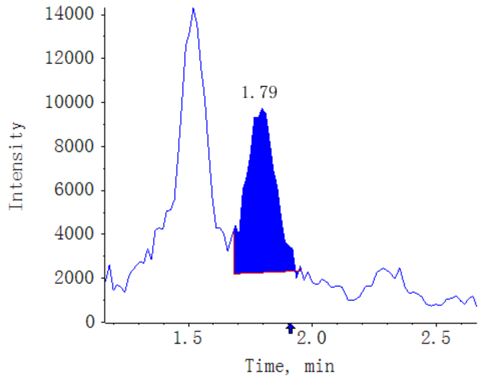

A20024802a_b
C07 AREA:5.937e4 S/N:30.9

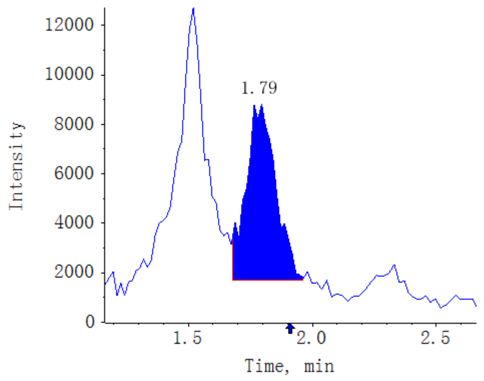

A20024805a_a
C07 AREA:7.998e6 S/N:84.5

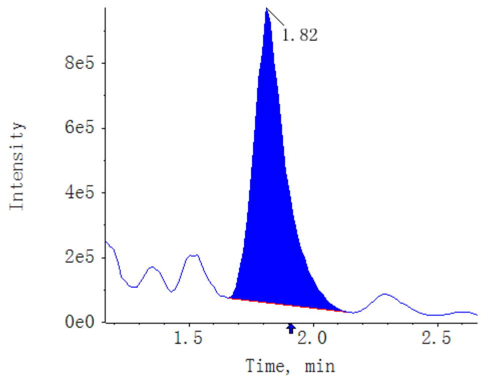

A20024805a_b
C07 AREA:8.711e6 S/N:78.3

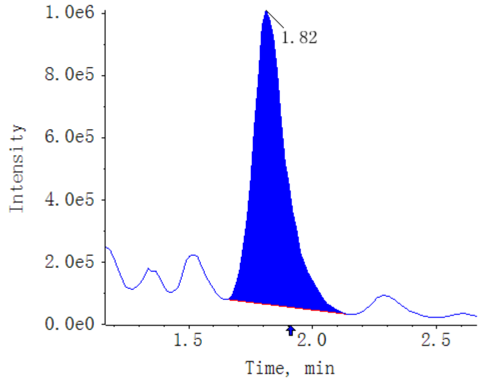

A20024808a_a
C07 AREA:8.762e5 S/N:62.9

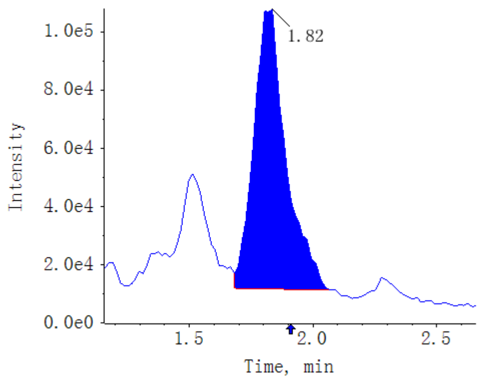

A20024808a_b
C07 AREA:8.875e5 S/N:77.1

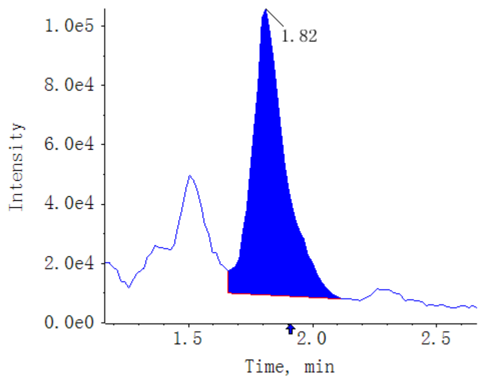

A20024811a_a
C07 AREA:5.235e4 S/N:14.6

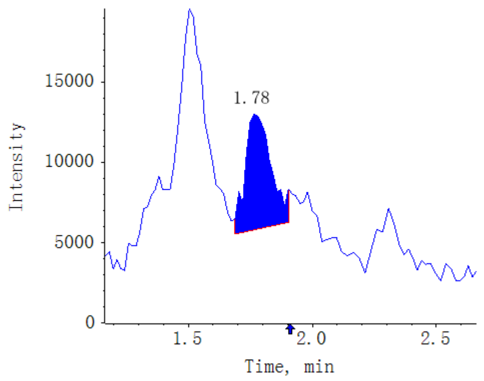

A20024811a_b
C07 AREA:5.623e4 S/N:17.7

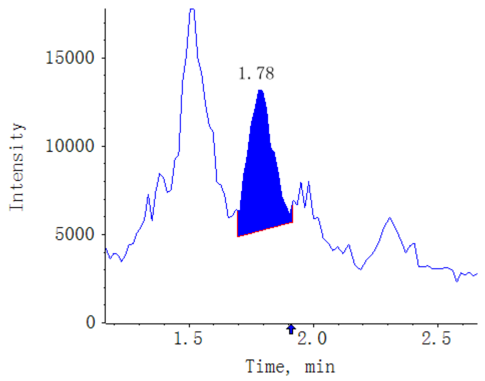

Compound name: β-Carotene
Regression Equation: y = 0.62745 x + 0.00632 (r = 0.99819) (weighting: 1 / x)

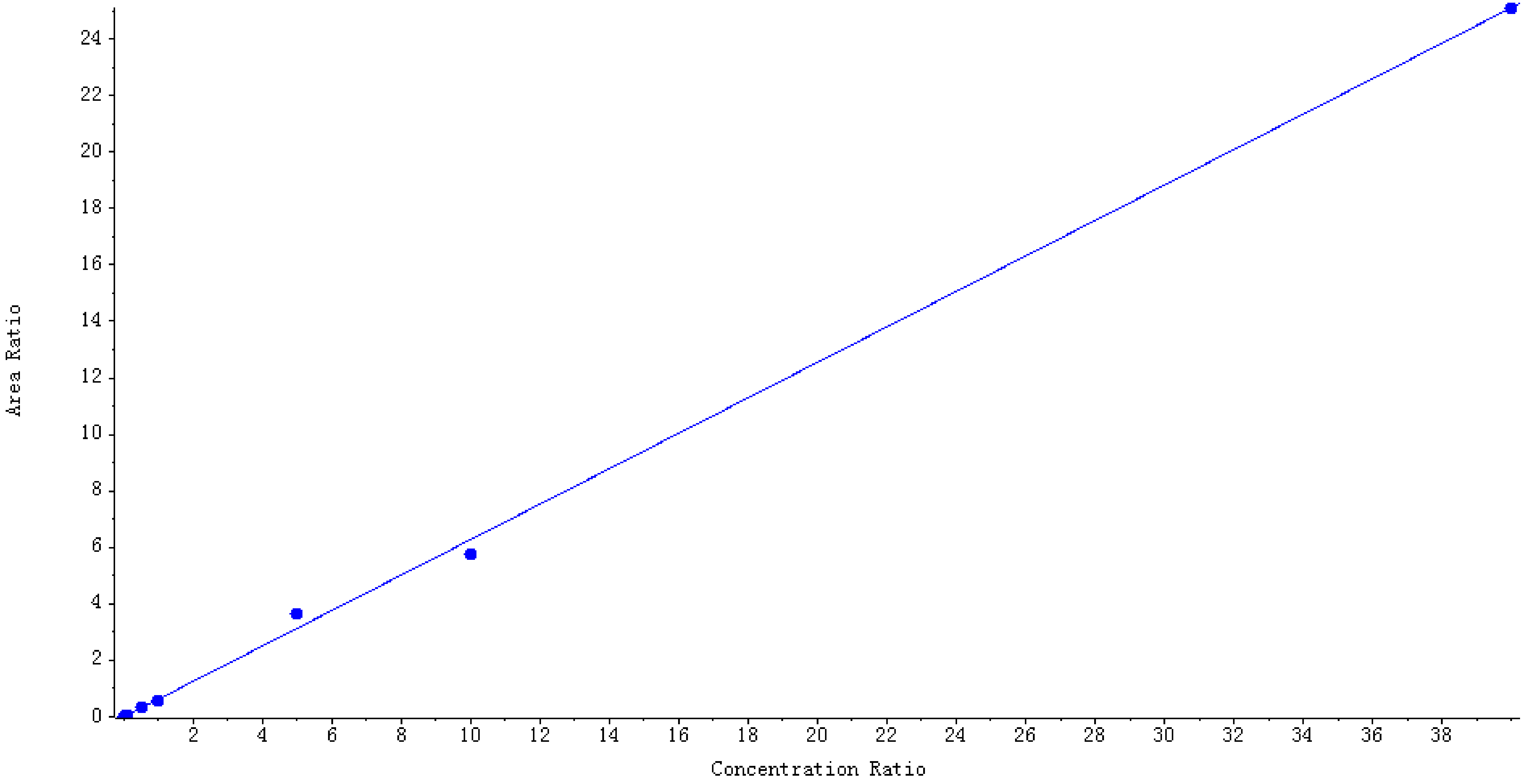

Peak Review

BLANK
C08 AREA:N/A S/N:N/A

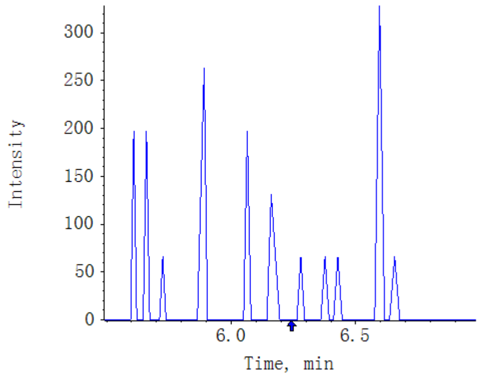

MWMS_20200904_1
C08 AREA:3.014e5 S/N:56.7

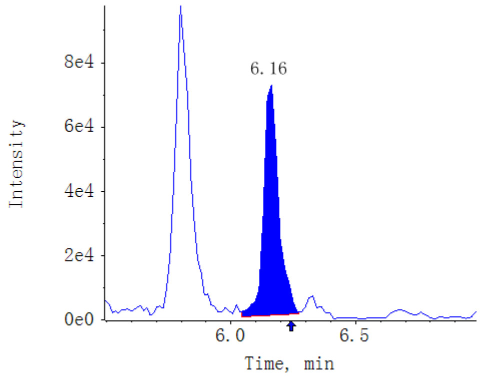

A20024797a_a
C08 AREA:5.318e6 S/N:177.5

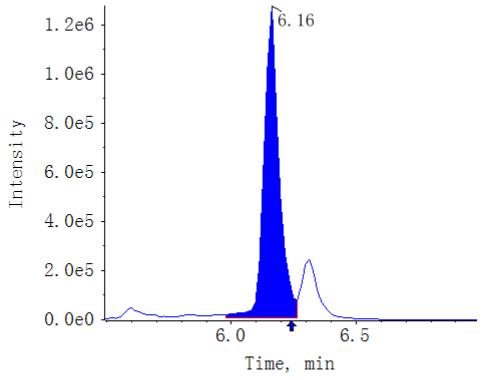

A20024797a_b
C08 AREA:6.792e6 S/N:177.7

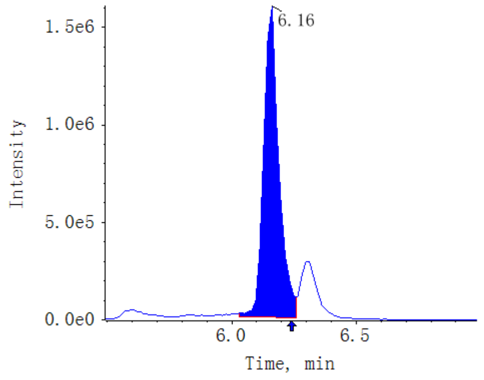

A20024800a_a
C08 AREA:1.879e6 S/N:224.4

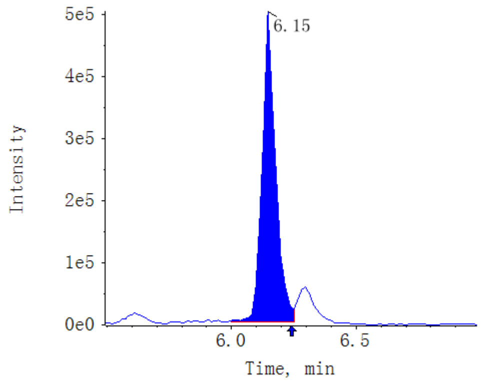

A20024800a_b
C08 AREA:1.817e6 S/N:275.4

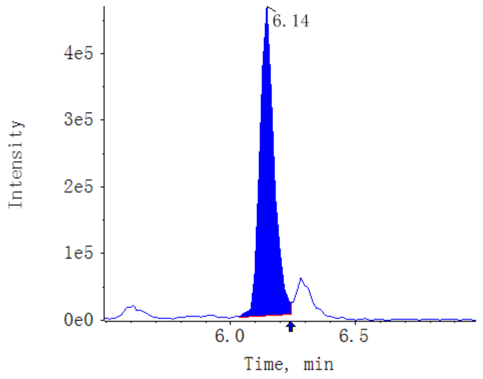

A20024802a_a
C08 AREA:2.473e6 S/N:159.4

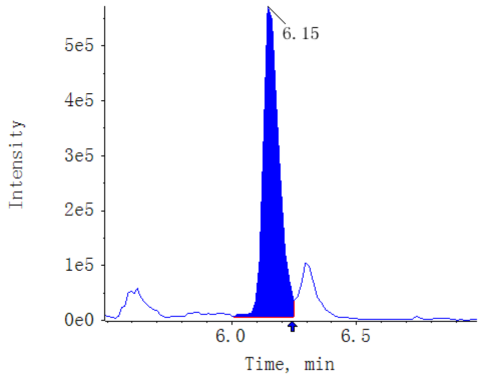

A20024802a_b
C08 AREA:1.897e6 S/N:191.8

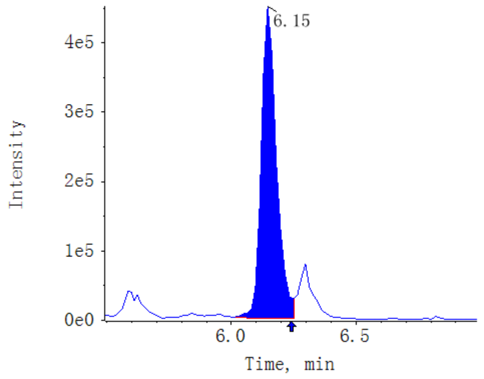

A20024805a_a
C08 AREA:6.827e6 S/N:172.7

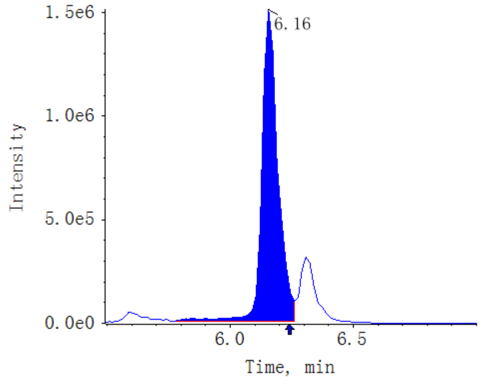

A20024805a_b
C08 AREA:7.303e6 S/N:176.6

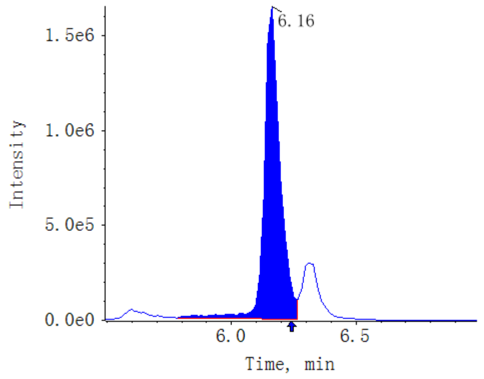

A20024808a_a
C08 AREA:1.043e6 S/N:241.1

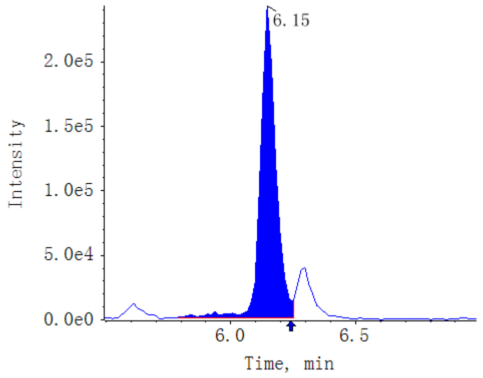

A20024808a_b
C08 AREA:9.574e5 S/N:229.9

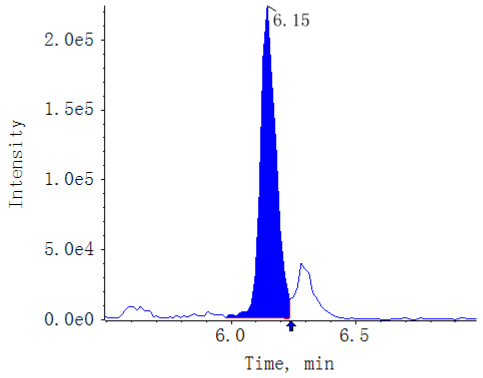

A20024811a_a
C08 AREA:1.652e6 S/N:171.5

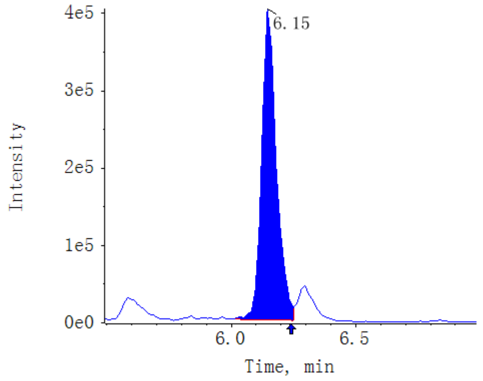

A20024811a_b
C08 AREA:1.665e6 S/N:156.2

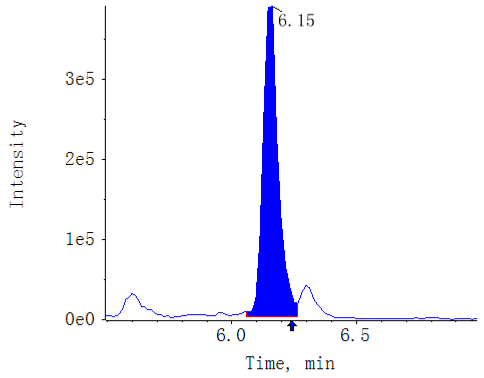

Compound name: Xanthophyll
Regression Equation: y = 0.93327 x + 9.41093e-4 (r = 0.99894) (weighting: 1 / x)

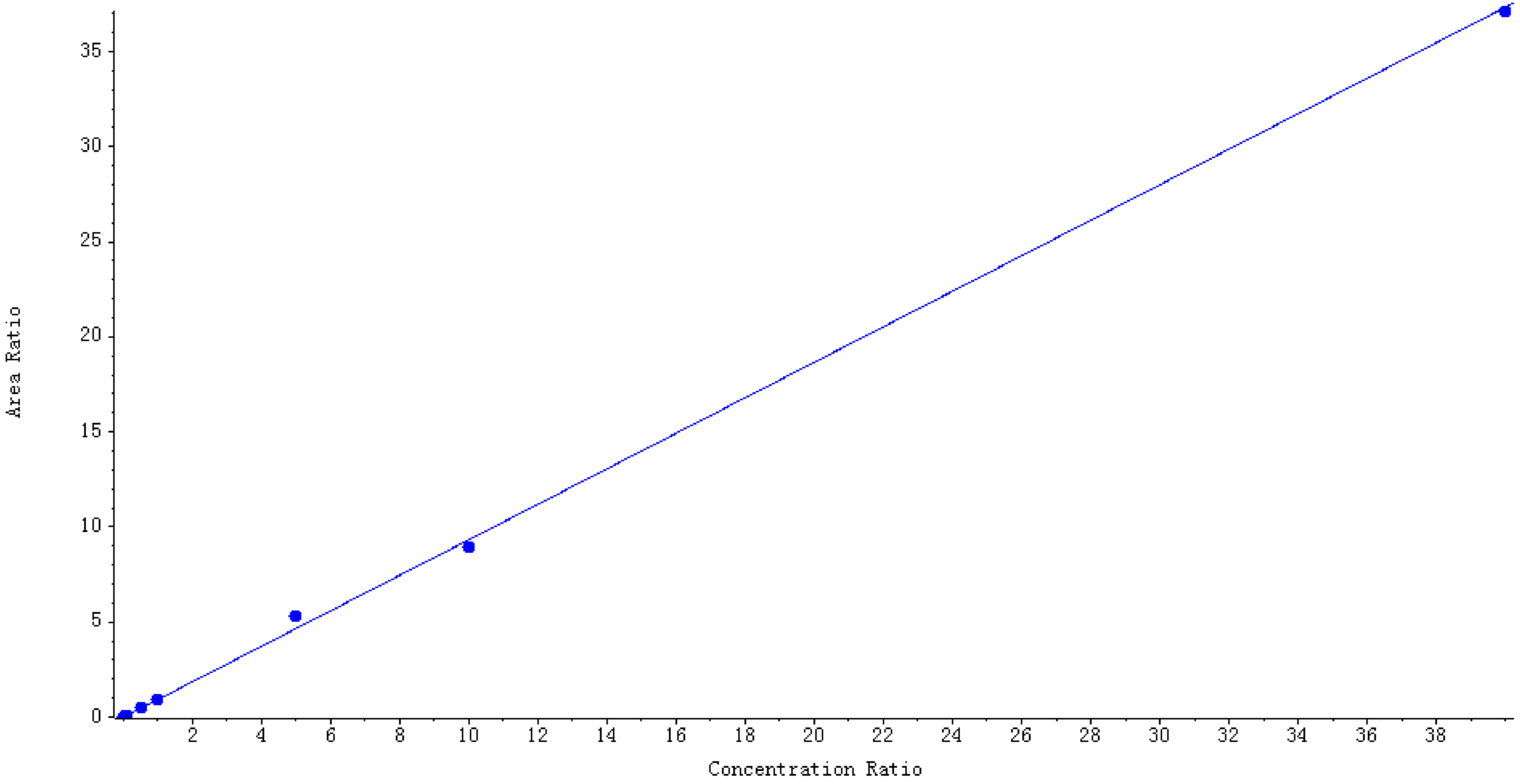

Peak Review

BLANK
C09 AREA:N/A S/N:N/A

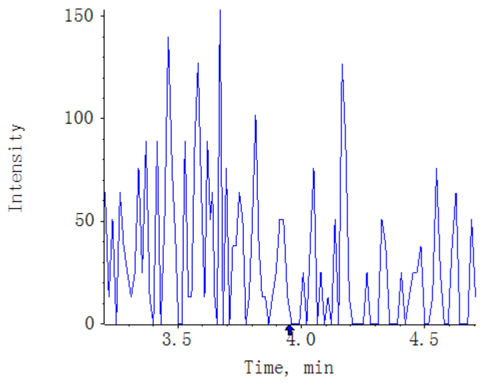

MWMS_20200904_1
C09 AREA:1.254e6 S/N:271.0

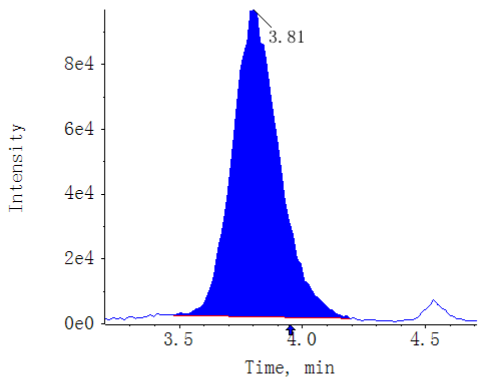

A20024797a_a
C09 AREA:3.104e7 S/N:449.4

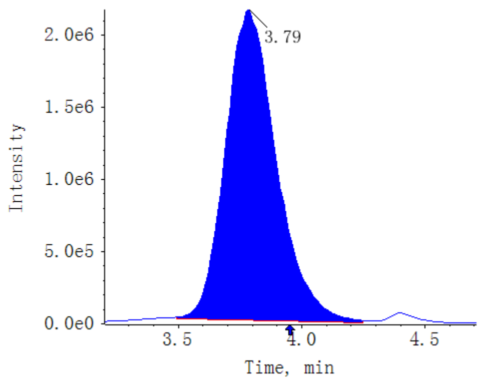

A20024797a_b
C09 AREA:3.638e7 S/N:503.5

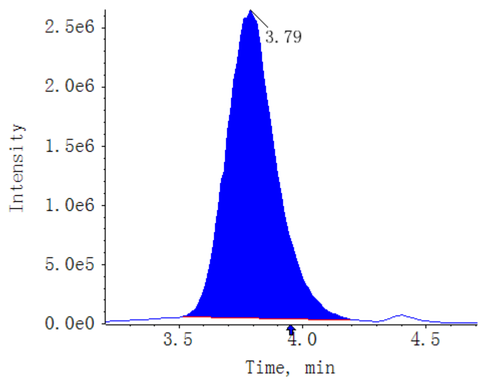

A20024800a_a
C09 AREA:3.376e6 S/N:188.5

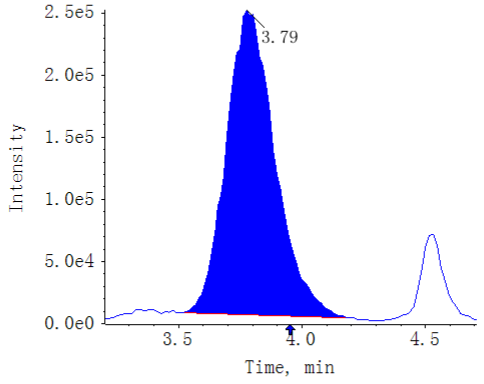

A20024800a_b
C09 AREA:3.435e6 S/N:190.7

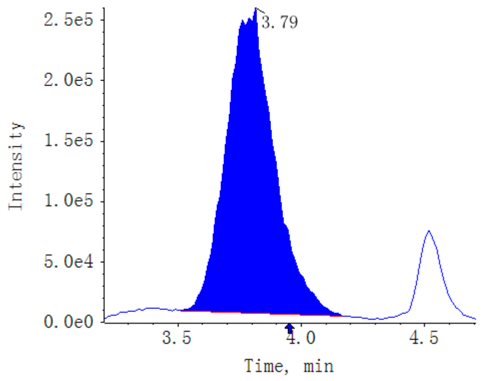

A20024802a_a
C09 AREA:2.487e5 S/N:52.4

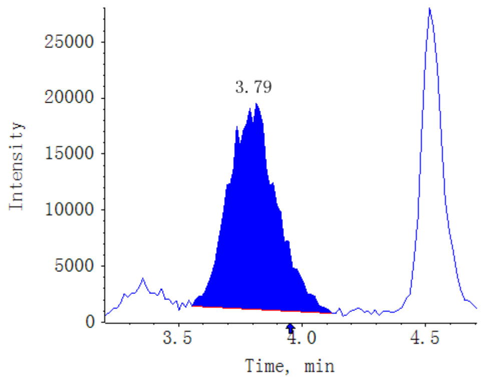

A20024802a_b
C09 AREA:2.124e5 S/N:67.0

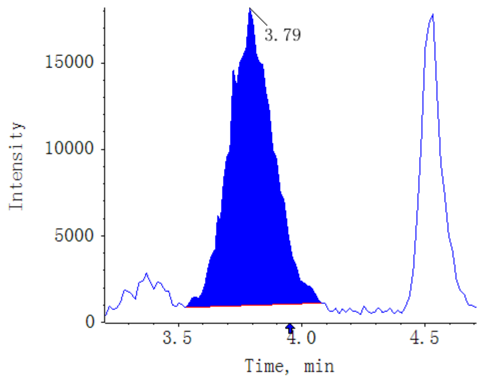

A20024805a_a
C09 AREA:3.951e7 S/N:444.8

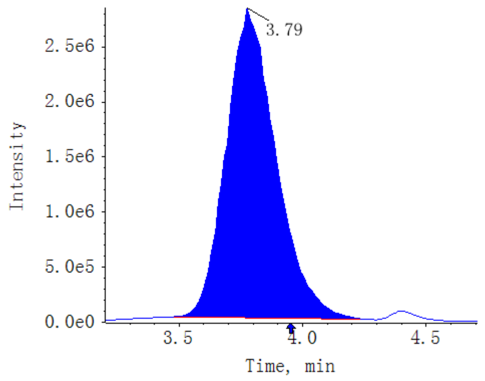

A20024805a_b
C09 AREA:4.042e7 S/N:438.4

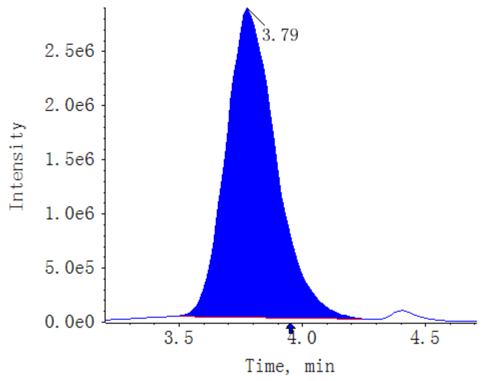

A20024808a_a
C09 AREA:6.987e6 S/N:187.1

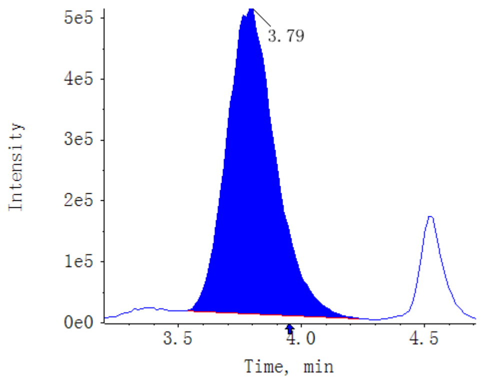

A20024808a_b
C09 AREA:6.293e6 S/N:207.4

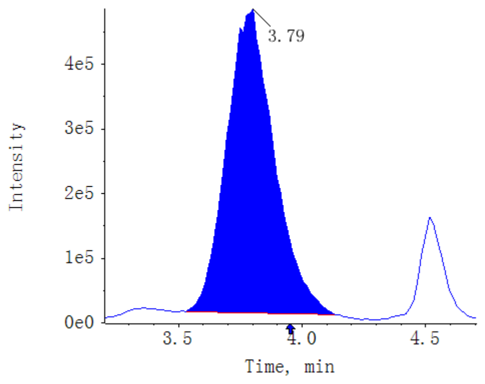

A20024811a_a
C09 AREA:1.576e6 S/N:99.3

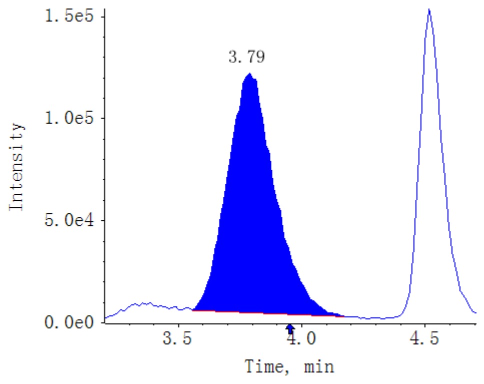

A20024811a_b
C09 AREA:1.500e6 S/N:94.2

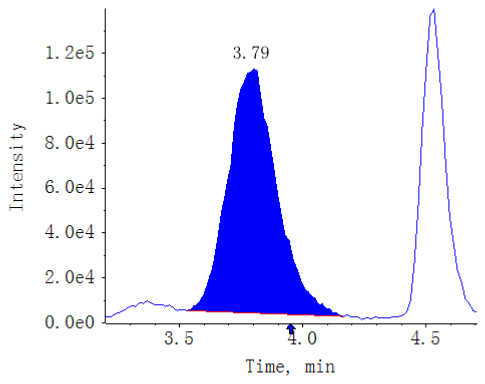

Compound name: β-Cryptoxanthin
Regression Equation: y = 0.34449 x + 0.00821 (r = 0.99717) (weighting: 1 / x)

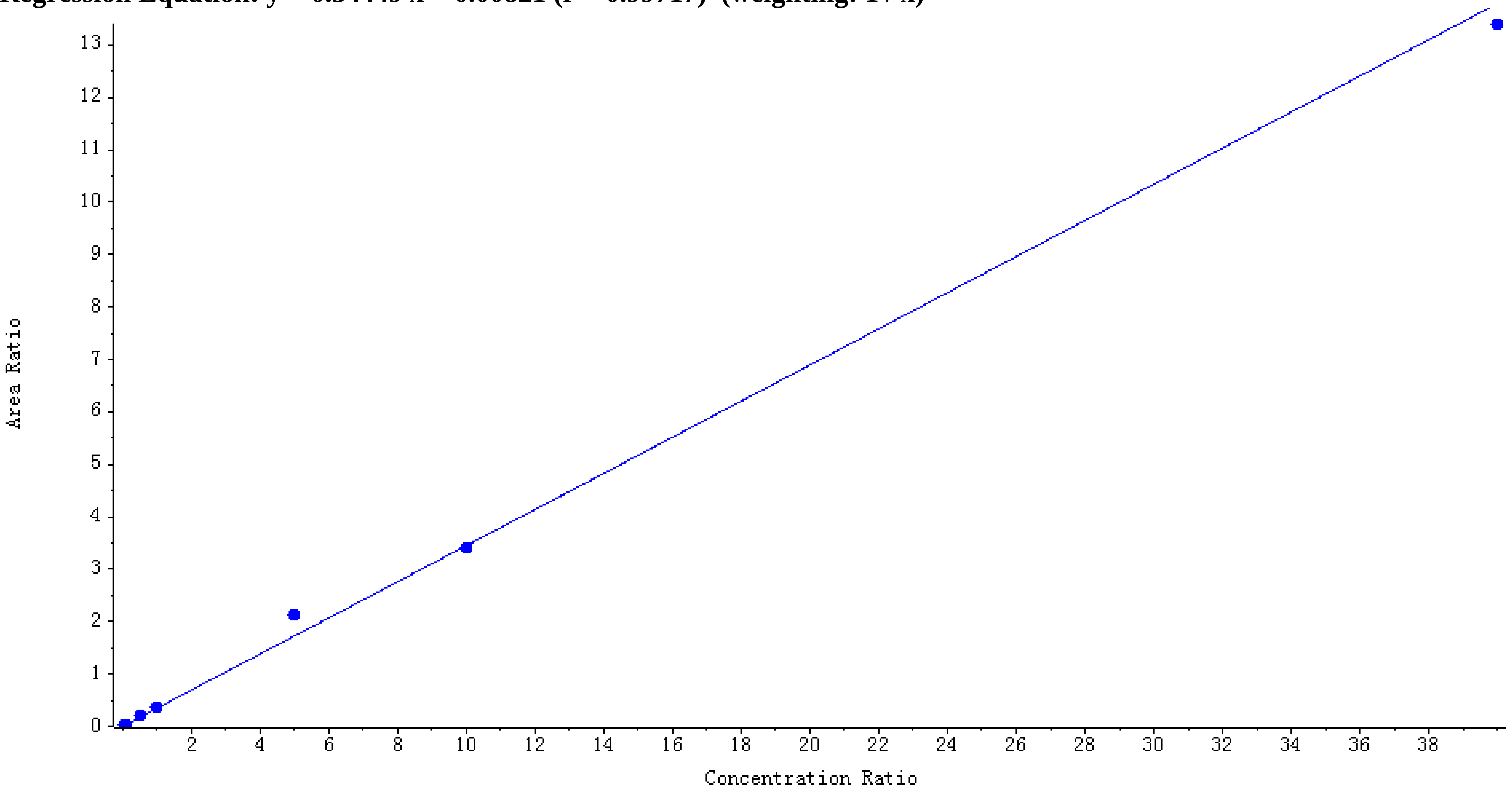

Peak Review

BLANK
C10 AREA:N/A S/N:N/A

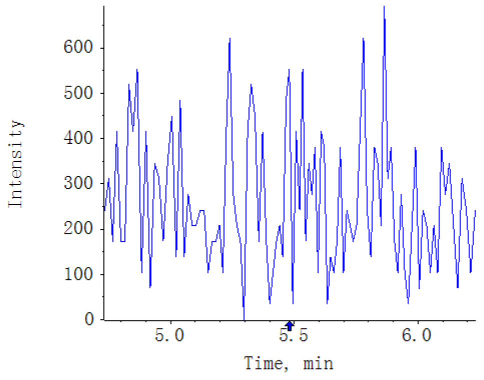

MWMS_20200904_1
C10 AREA:2.710e5 S/N:74.3

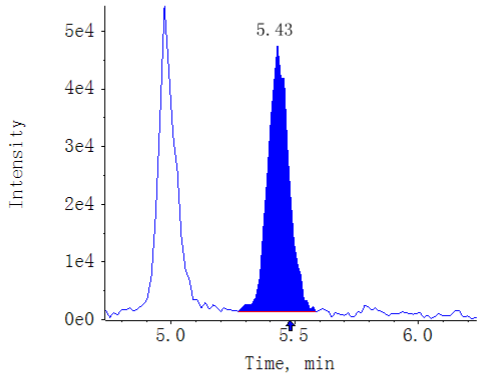

A20024797a_a
C10 AREA:2.200e5 S/N:28.9

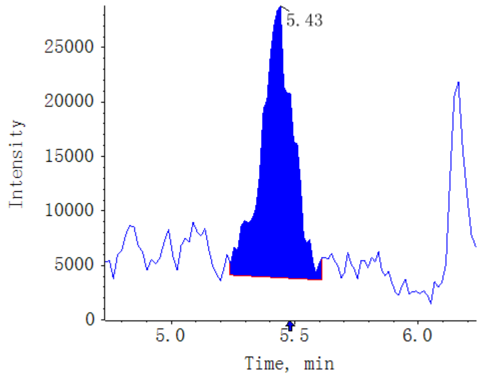

A20024797a_b
C10 AREA:2.482e5 S/N:36.5

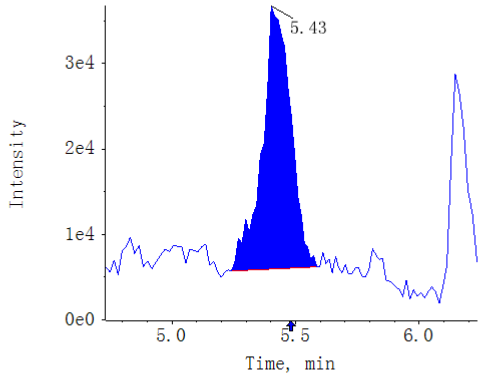

A20024800a_a
C10 AREA:1.176e5 S/N:44.2

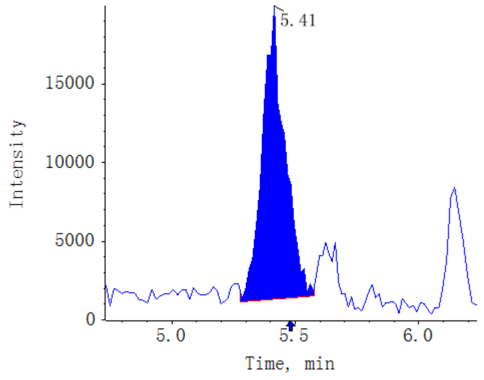

A20024800a_b
C10 AREA:1.180e5 S/N:37.0

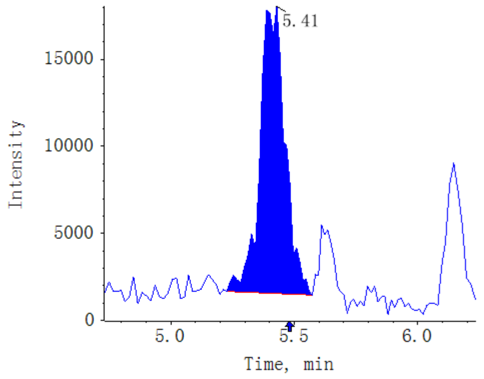

A20024802a_a
C10 AREA:1.825e5 S/N:65.1

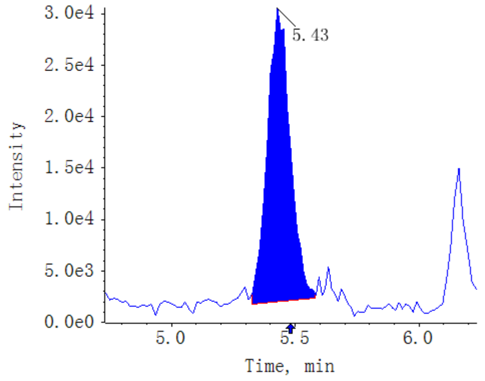

A20024802a_b
C10 AREA:1.183e5 S/N:45.9

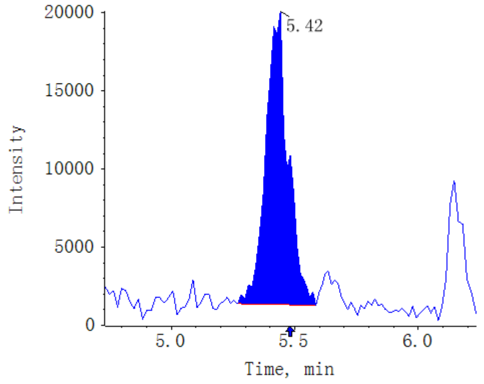

A20024805a_a
C10 AREA:3.038e5 S/N:35.7

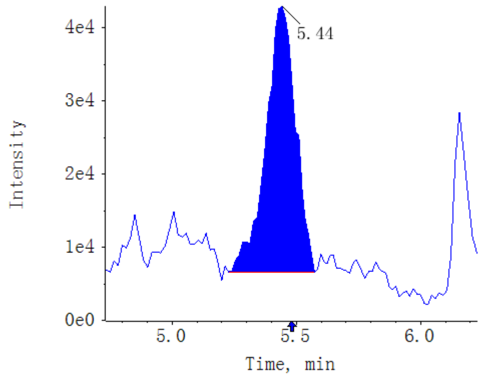

A20024805a_b
C10 AREA:3.617e5 S/N:31.7

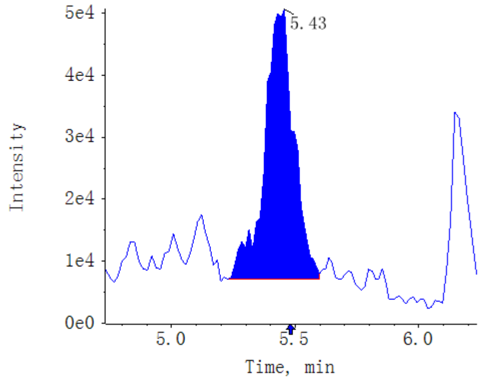

A20024808a_a
C10 AREA:7.289e4 S/N:38.2

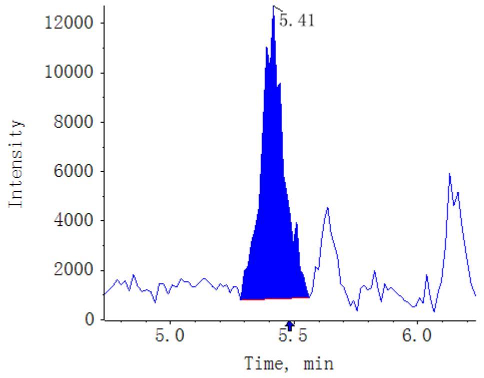

A20024808a_b
C10 AREA:6.896e4 S/N:32.4

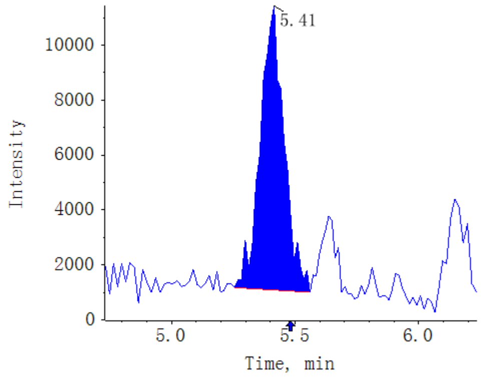

A20024811a_a
C10 AREA:1.946e5 S/N:66.7

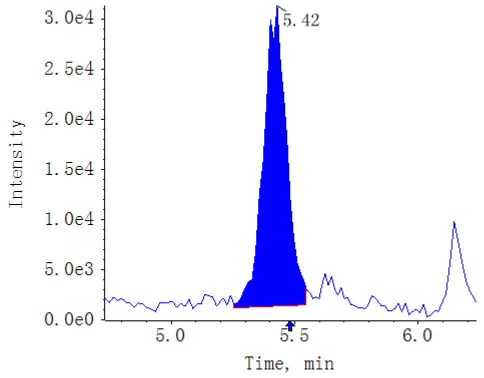

A20024811a_b
C10 AREA:1.849e5 S/N:70.1

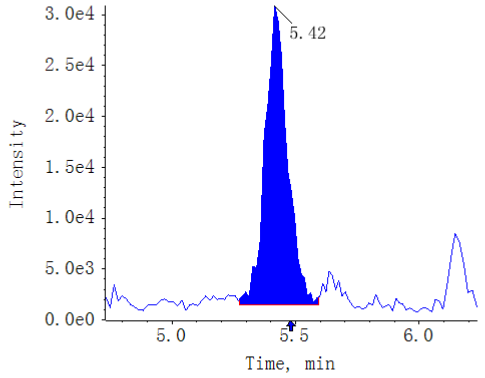

Compound name: Apocarote-l

Regression Equation: y = 9.04559 x + -1.24542 (r = 0.99911) (weighting: 1 / x)

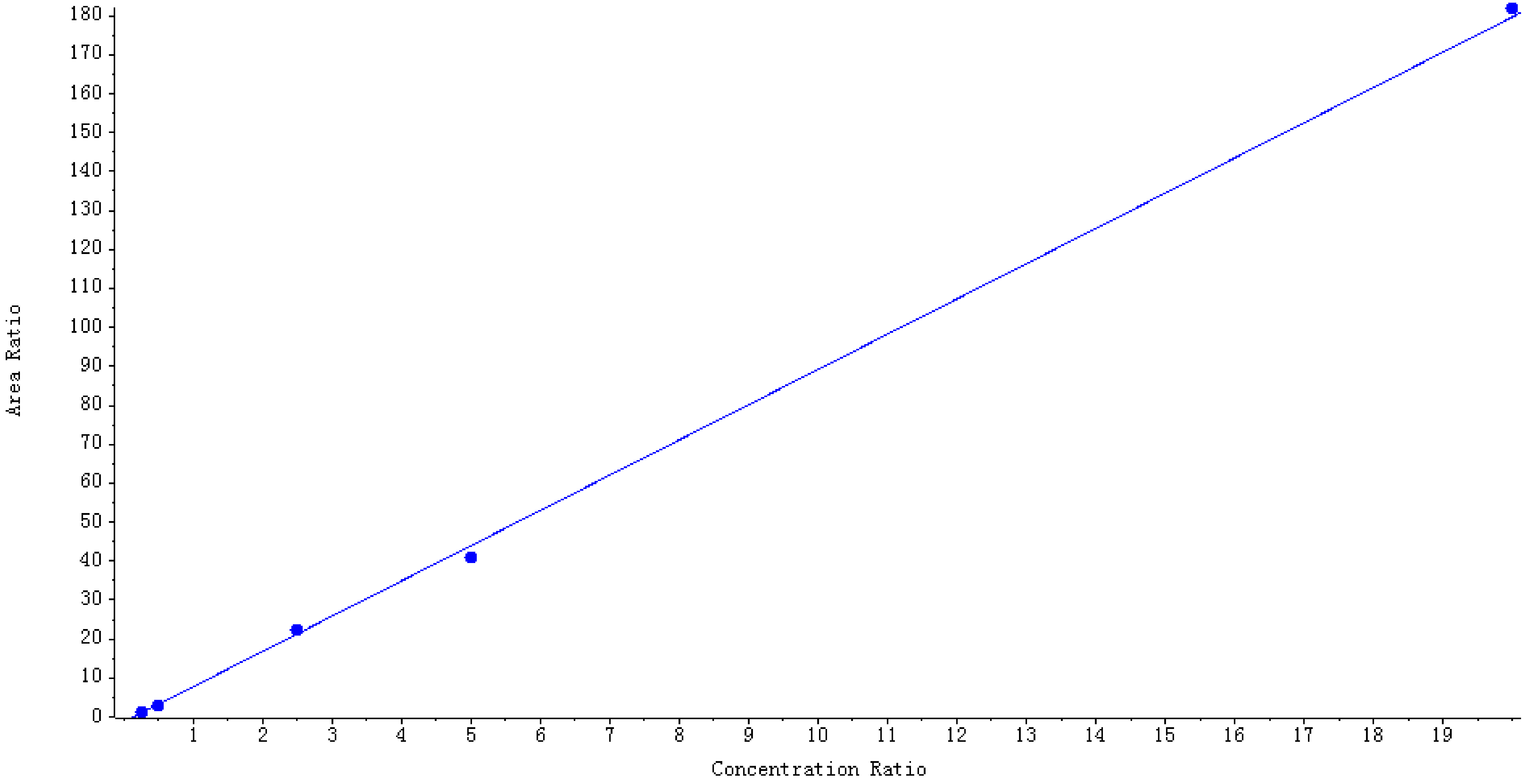

Peak Review

BLANK
C12 AREA:N/A S/N:N/A

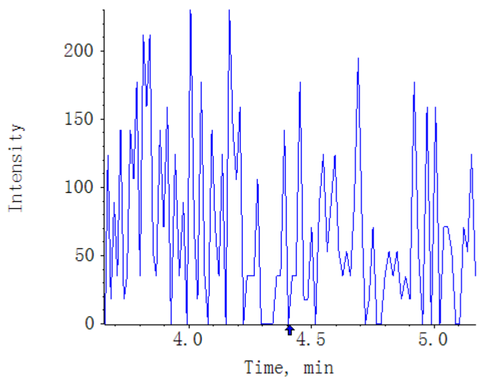

MWMS_20200904_1
C12 AREA:4.450e6 S/N:521.0

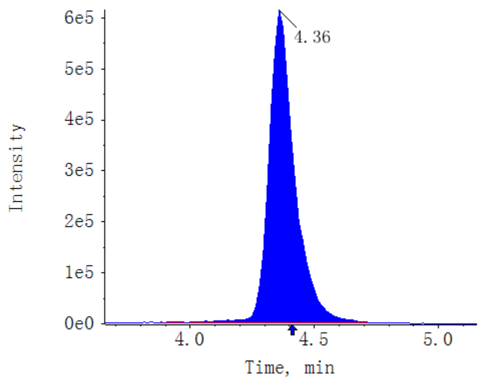

A20024797a_a
C12 AREA:2.760e4 S/N:53.1

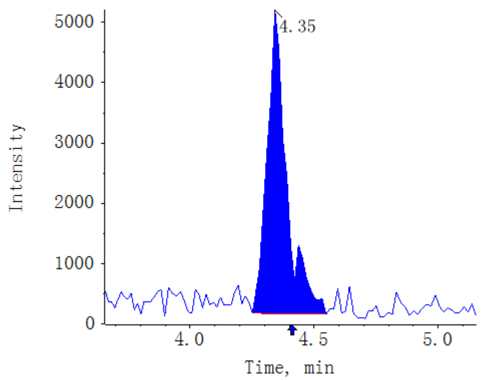

A20024797a_b
C12 AREA:3.368e4 S/N:39.6

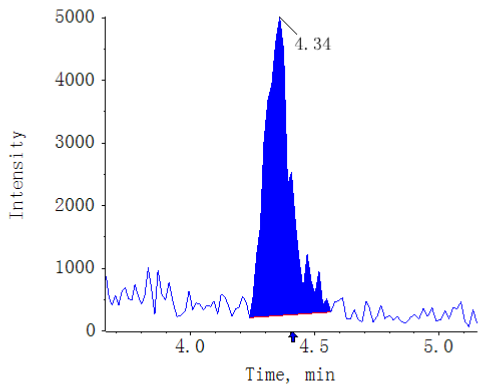

A20024800a_a
C12 AREA:1.006e4 S/N:17.6

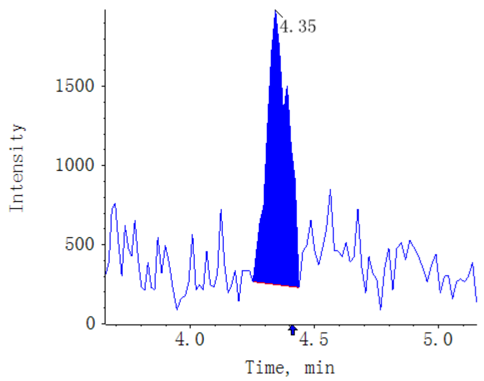

A20024800a_b
C12 AREA:8.674e3 S/N:12.5

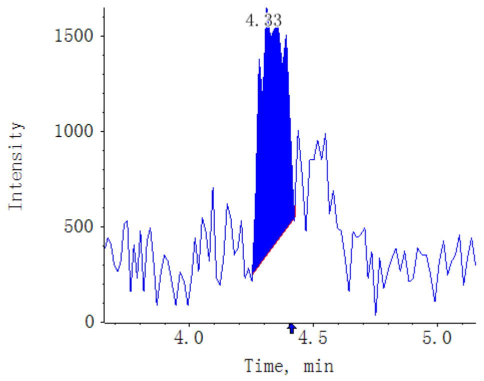

A20024802a_a
C12 AREA:2.613e4 S/N:34.0

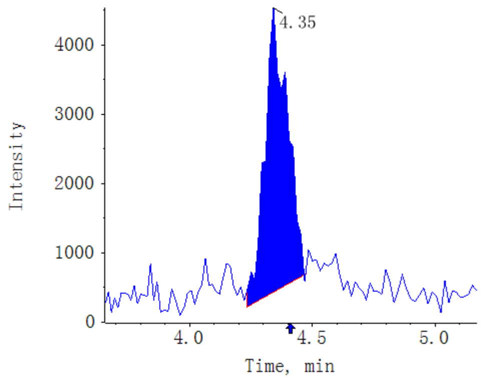

A20024802a_b
C12 AREA:1.577e4 S/N:18.3

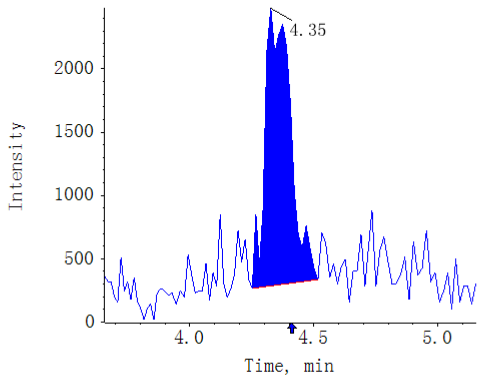

A20024805a_a
C12 AREA:4.444e4 S/N:46.1

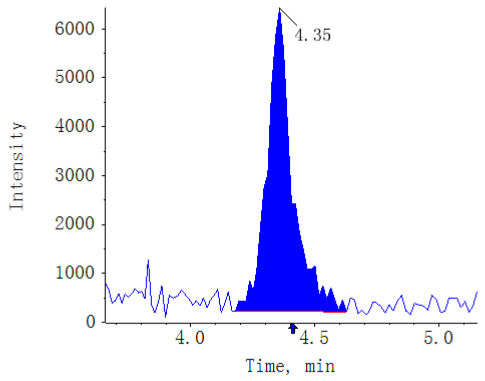

A20024805a_b
C12 AREA:3.926e4 S/N:44.3

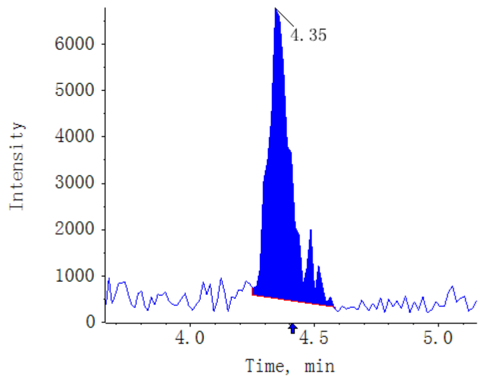

A20024808a_a
C12 AREA:5.348e3 S/N:10.9

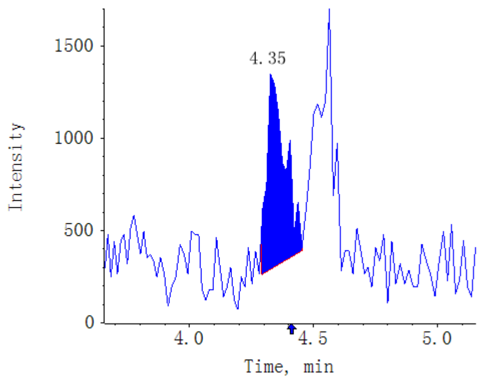

A20024808a_b
C12 AREA:4.042e3 S/N:9.4

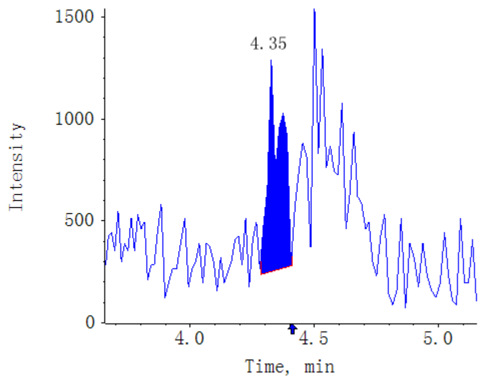

A20024811a_a
C12 AREA:2.849e4 S/N:47.1

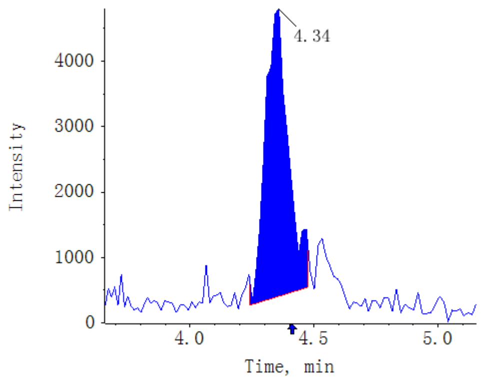

A20024811a_b
C12 AREA:2.772e4 S/N:41.7

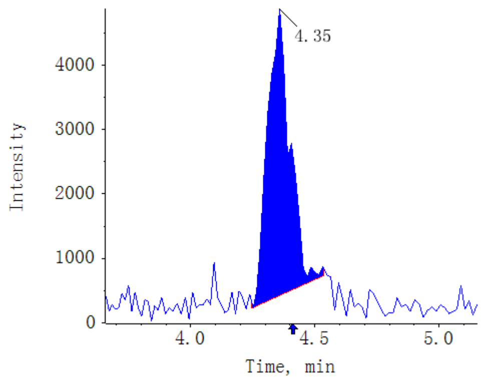

Compound name: (E/Z)-Phytoene
Regression Equation: y = 0.02355 x + 0.00243 (r = 0.99950) (weighting: 1 / x)

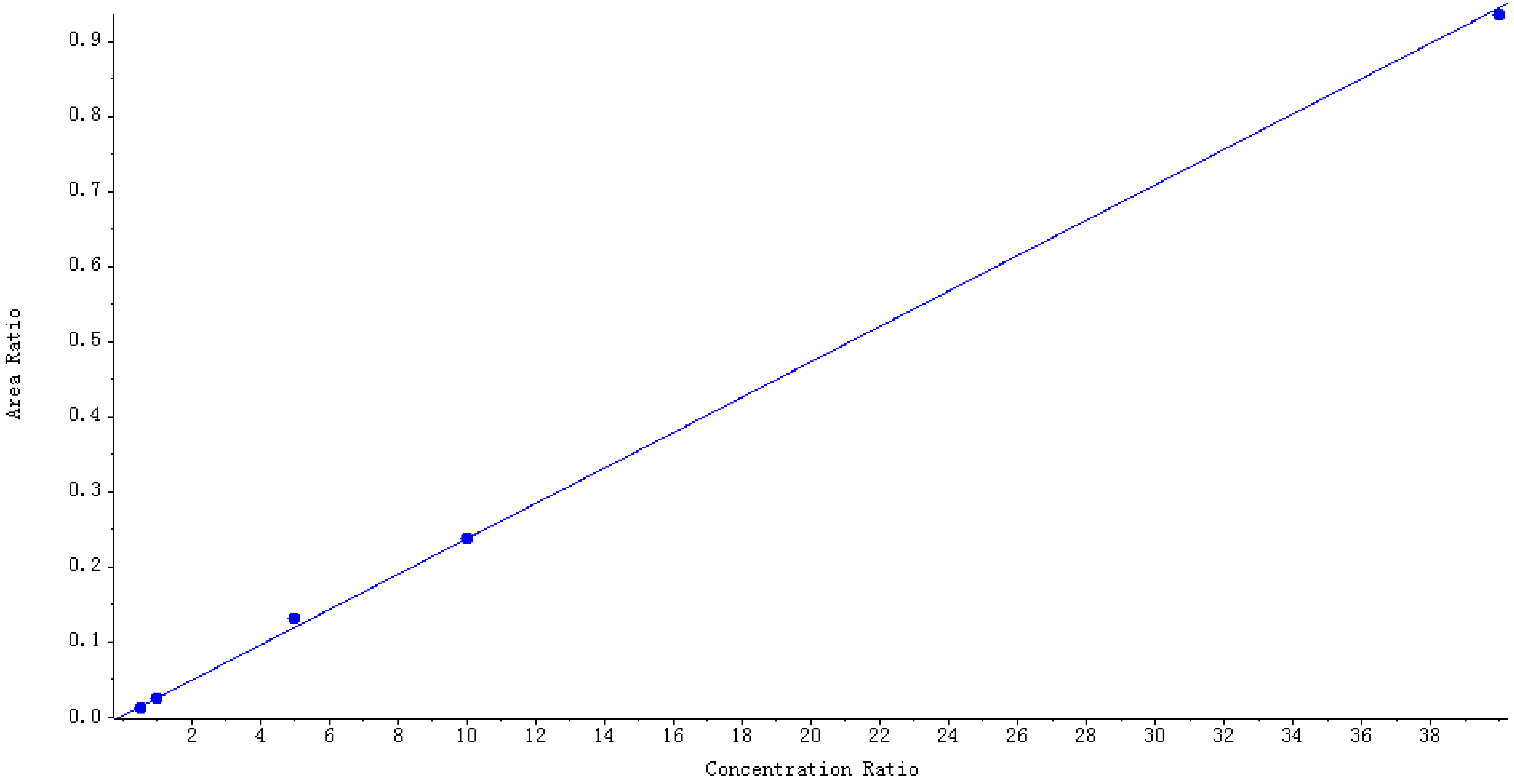

Peak Review

BLANK
C15 AREA:N/A S/N:N/A

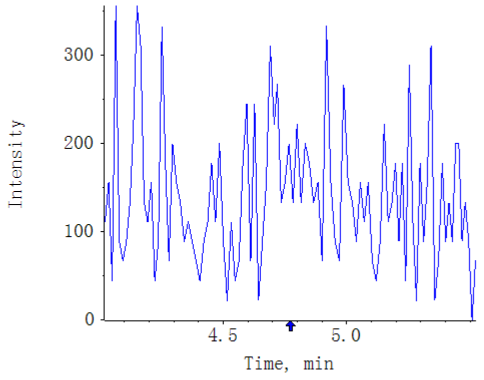

MWMS_20200904_1
C15 AREA:4.880e4 S/N:64.0

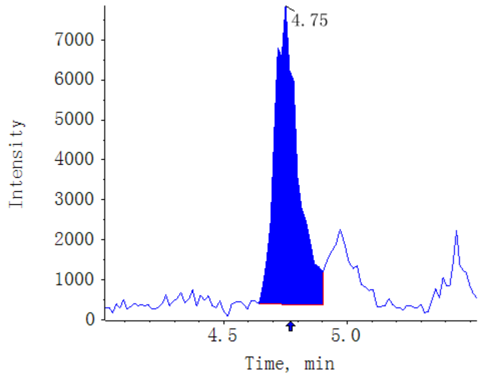

A20024797a_a
C15 AREA:1.999e5 S/N:50.6

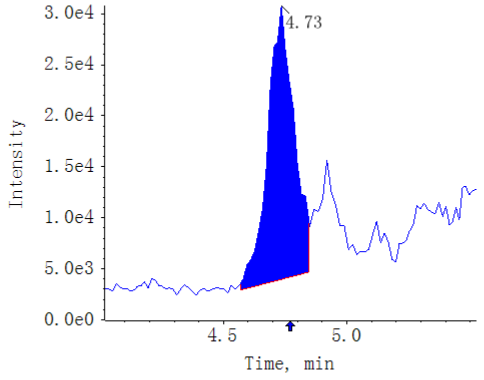

A20024797a_b
C15 AREA:2.326e5 S/N:36.8

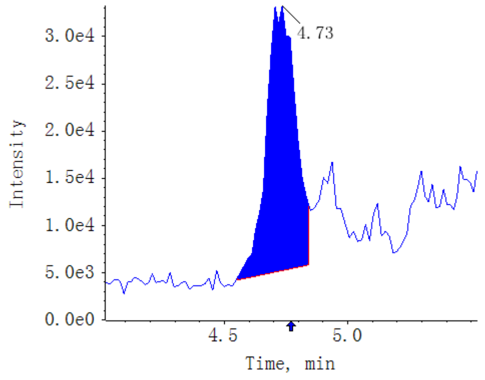

A20024800a_a
C15 AREA:9.158e4 S/N:25.4

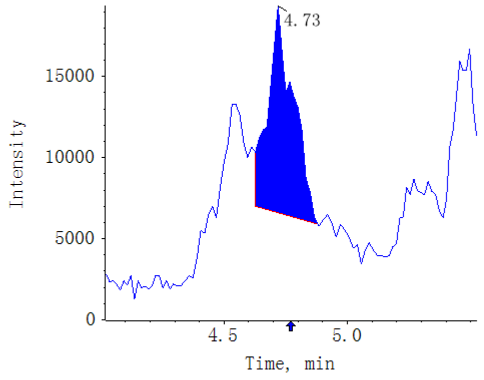

A20024800a_b
C15 AREA:1.089e5 S/N:30.2

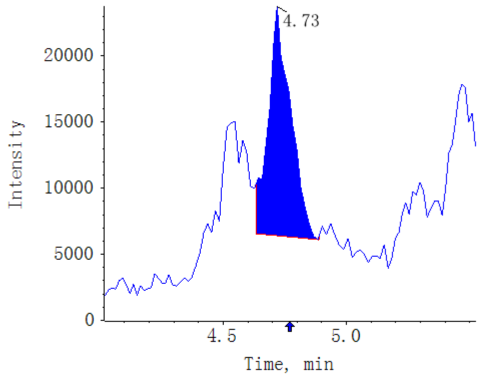

A20024802a_a
C15 AREA:6.122e5 S/N:128.9

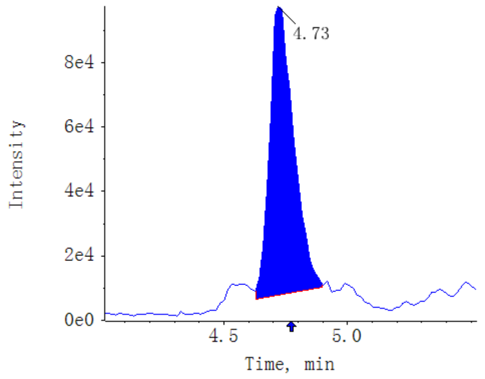

A20024802a_b
C15 AREA:6.112e5 S/N:133.4

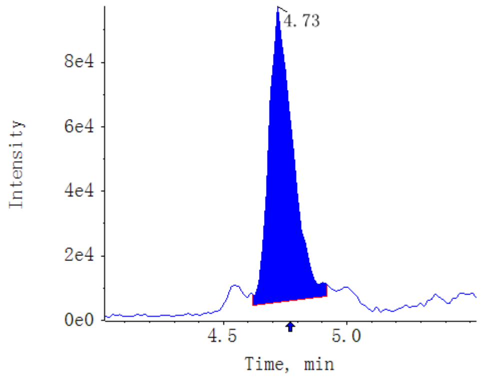

A20024805a_a
C15 AREA:2.258e5 S/N:31.1

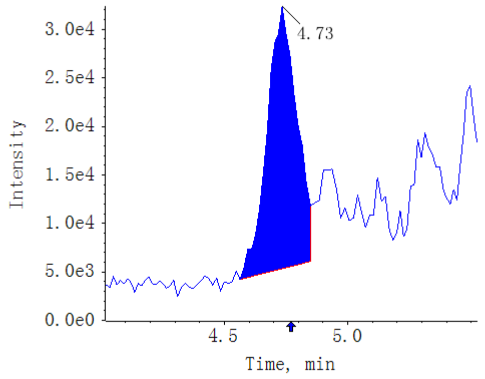

A20024805a_b
C15 AREA:1.930e5 S/N:31.5

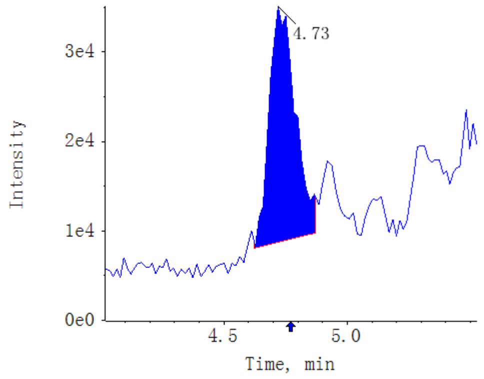

A20024808a_a
C15 AREA:9.490e4 S/N:21.3

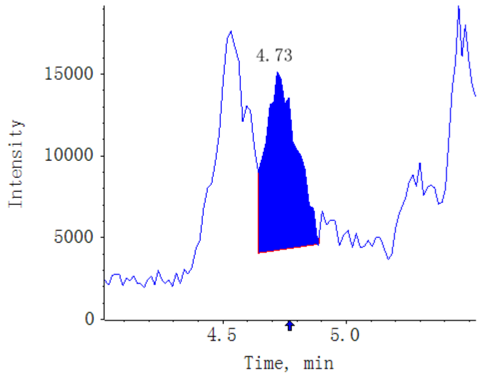

A20024808a_b
C15 AREA:9.330e4 S/N:22.7

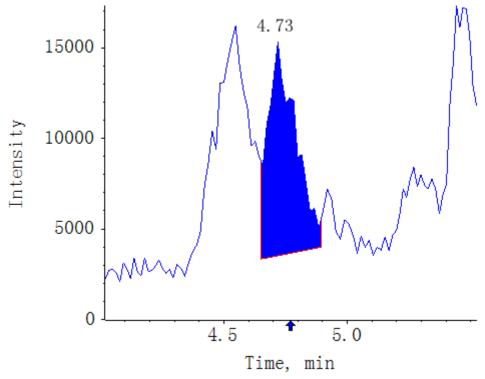

A20024811a_a
C15 AREA:1.314e6 S/N:216.1

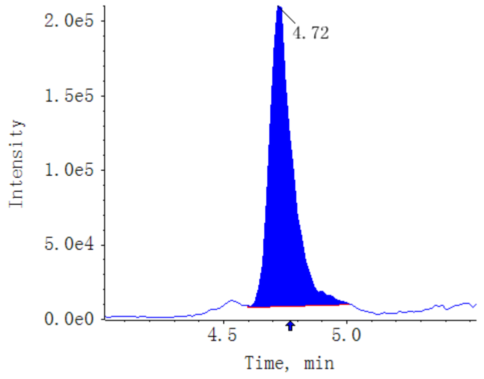

A20024811a_b
C15 AREA:1.251e6 S/N:186.5

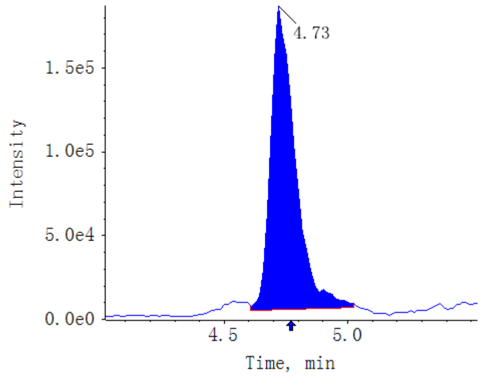

Compound name: lutein palmitate
Regression Equation: y = 0.93327 x + 9.41093e-4 (r = 0.99894) (weighting: 1 / x)

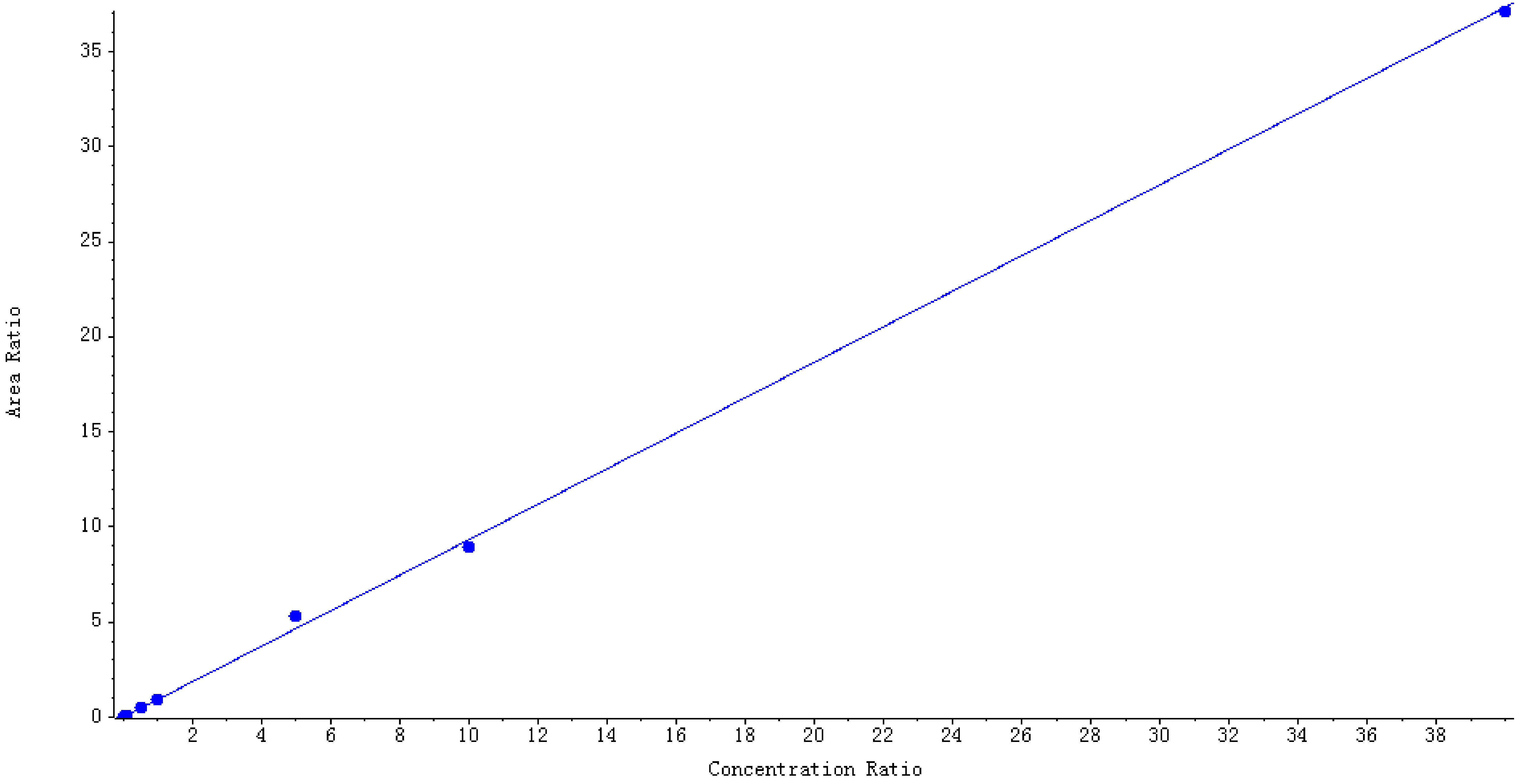

Peak Review

BLANK
lut(C16:0) AREA:N/A S/N:N/A

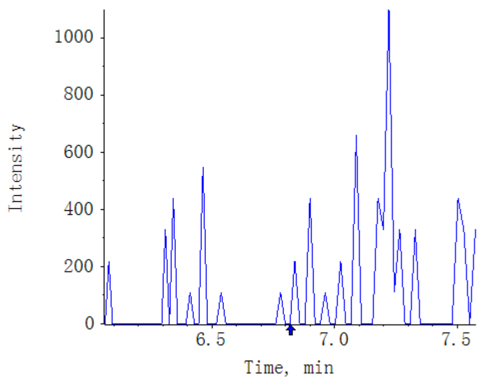

MWMS_20200904_1
lut(C16:0) AREA:N/A S/N:N/A

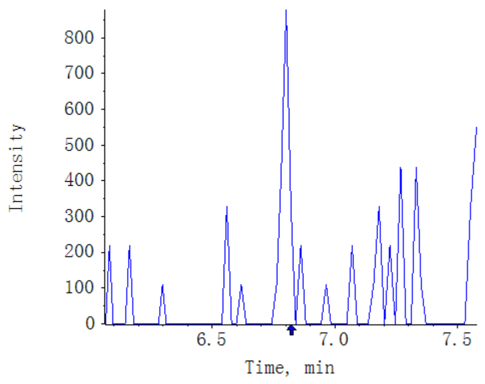

A20024797a_a
lut(C16:0) AREA:6.374e4
S/N:20.4

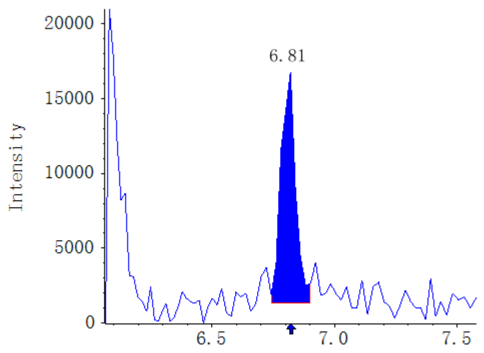

A20024797a_b
lut(C16:0) AREA:6.686e4
S/N:23.2

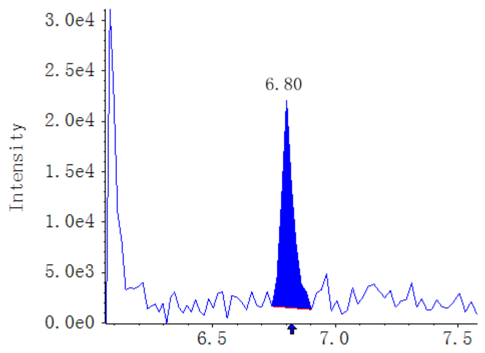

A20024800a_a
lut(C16:0) AREA:3.519e6
S/N:93.8

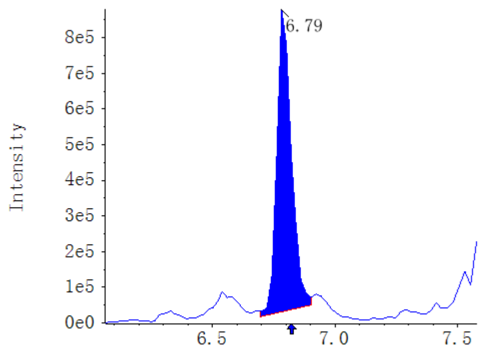

A20024800a_b
lut(C16:0) AREA:3.269e6
S/N:95.7

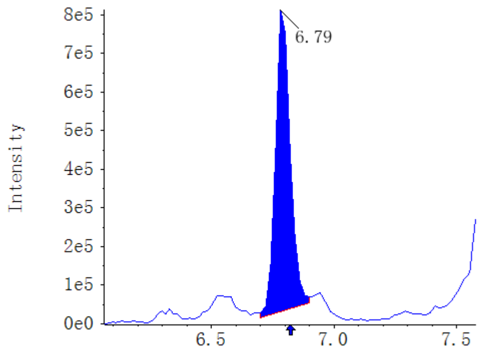

A20024802a_a
lut(C16:0) AREA:3.364e6
S/N:102.3

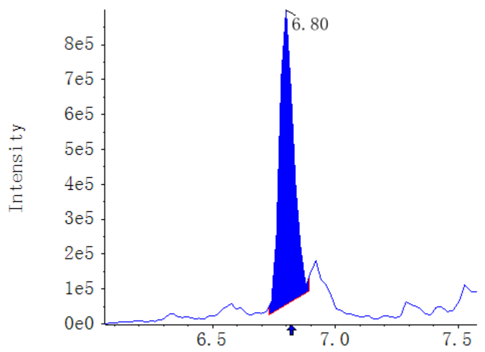

A20024802a_b
lut(C16:0) AREA:2.079e6
S/N:75.0

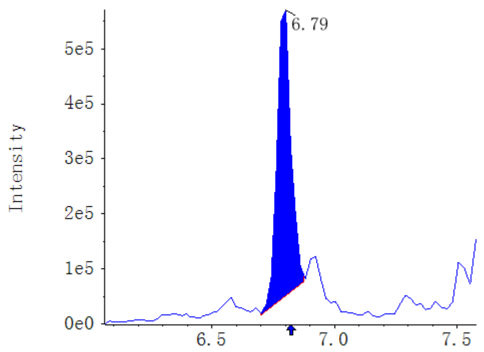

A20024805a_a
lut(C16:0) AREA:7.620e4
S/N:20.9

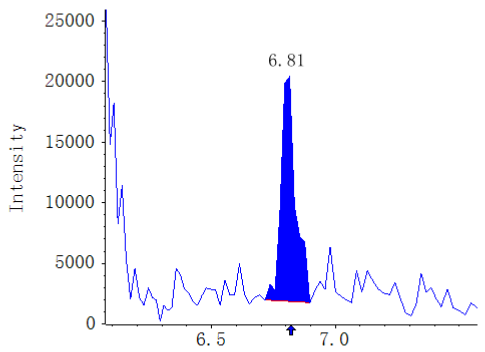

A20024805a_b
lut(C16:0) AREA:7.313e4
S/N:13.4

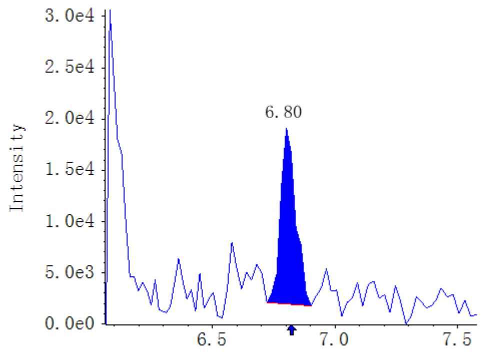

A20024808a_a
lut(C16:0) AREA:1.055e6
S/N:46.7

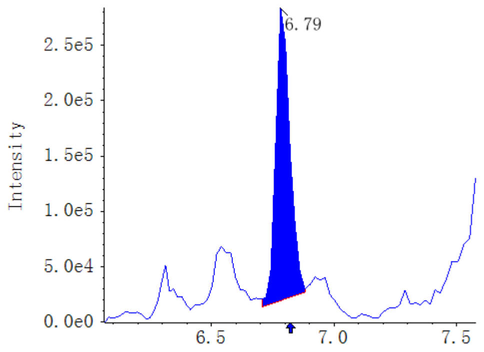

A20024808a_b
lut(C16:0) AREA:9.260e5
S/N:35.8

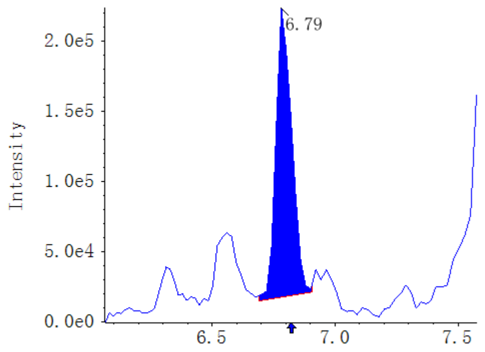

A20024811a_a
lut(C16:0) AREA:6.790e5
S/N:42.5

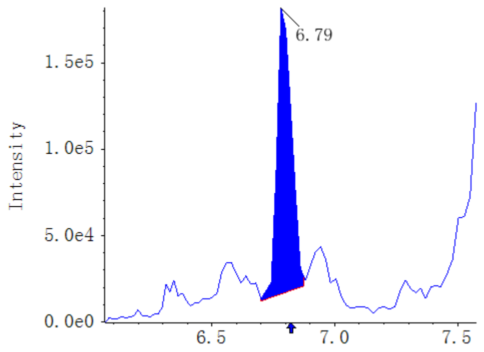

A20024811a_b
lut(C16:0) AREA:6.081e5
S/N:44.0

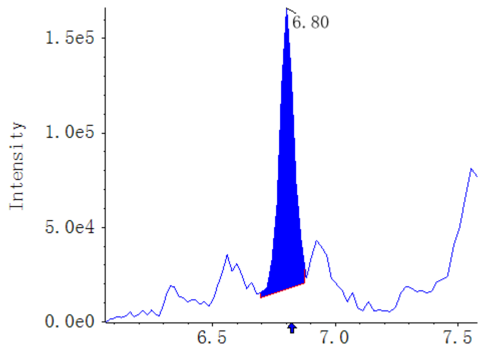

Compound name: lutein oleate
Regression Equation: y = 0.93327 x + 9.41093e-4 (r = 0.99894) (weighting: 1 / x)

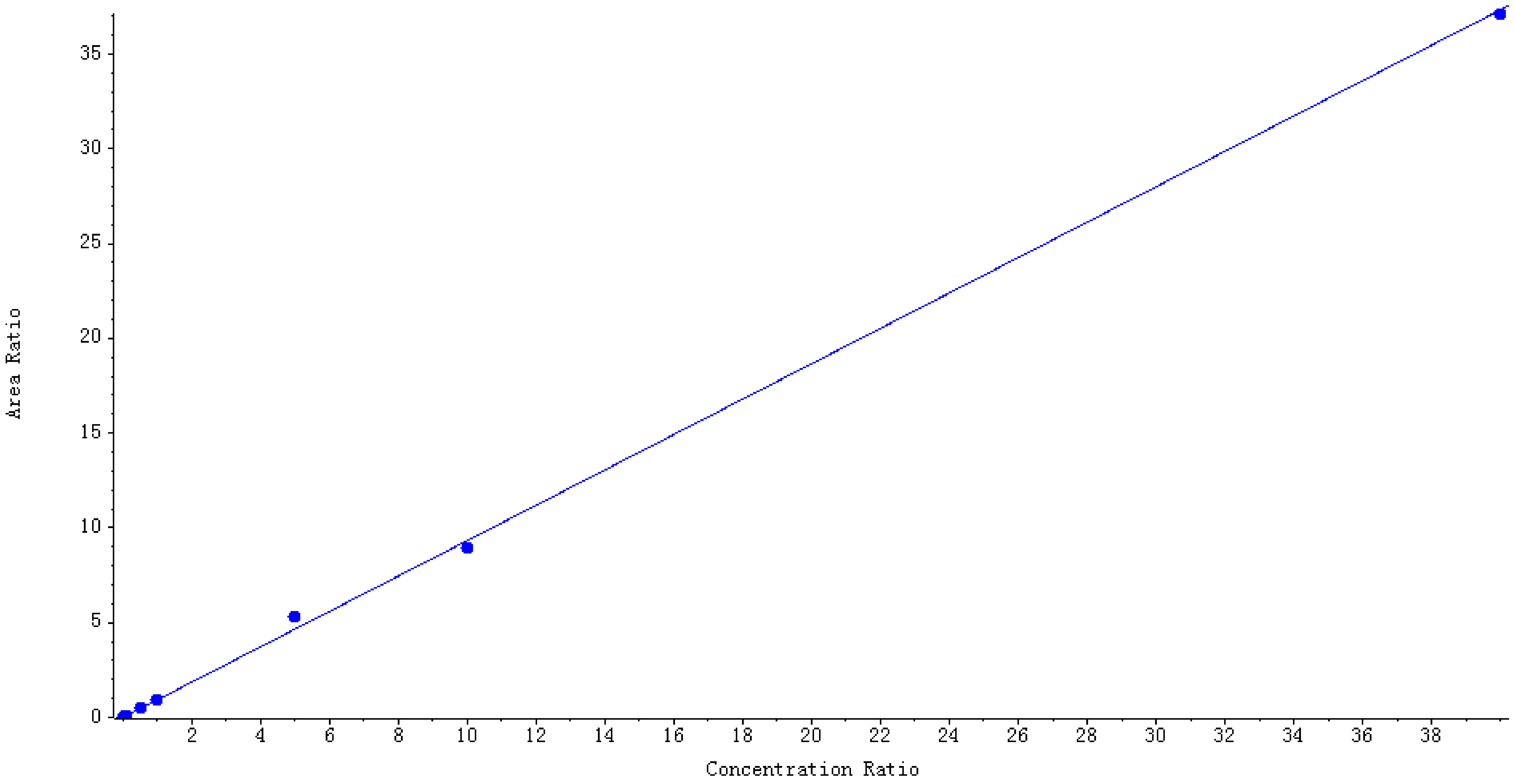

Peak Review

BLANK
lut(C18:1) AREA:N/A S/N:N/A

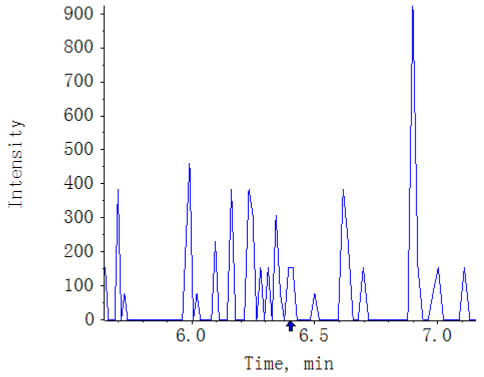

MWMS_20200904_1
lut(C18:1) AREA:N/A S/N:N/A

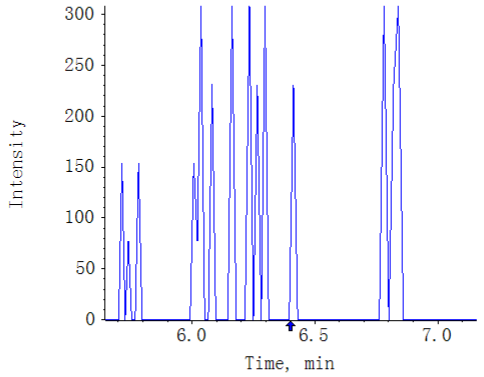

A20024797a_a
lut(C18:1) AREA:4.851e4 S/N:27.7

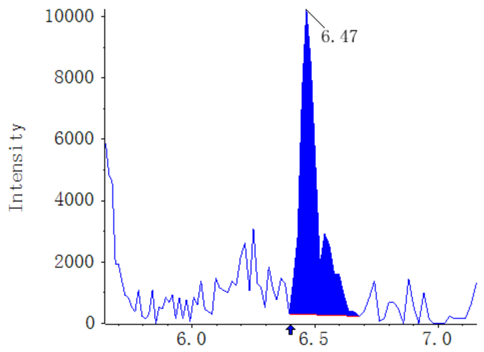

A20024797a_b
lut(C18:1) AREA:5.481e4 S/N:29.9

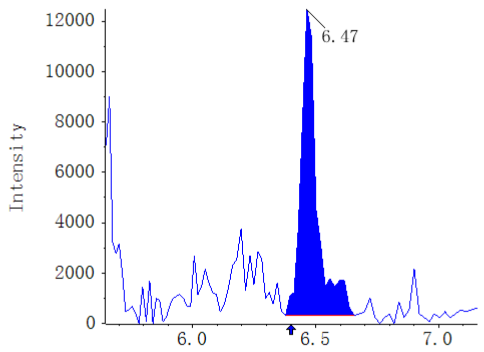

A20024800a_a
lut(C18:1) AREA:1.653e5 S/N:8.3

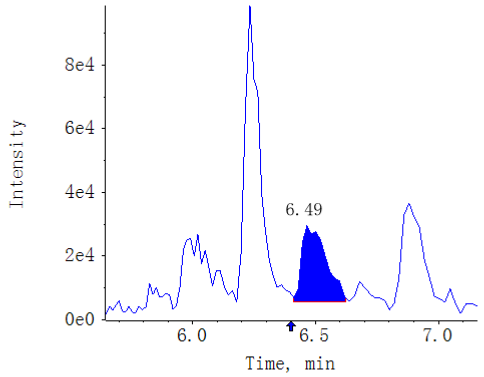

A20024800a_b
lut(C18:1) AREA:1.523e5 S/N:10.3

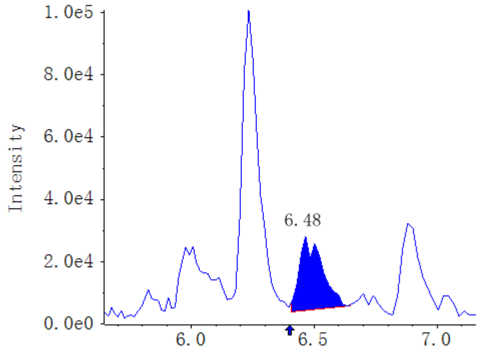

A20024802a_a
lut(C18:1) AREA:1.107e5 S/N:9.2

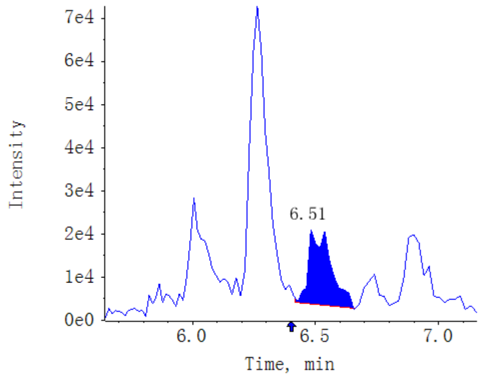

A20024802a_b
lut(C18:1) AREA:7.511e4 S/N:8.8

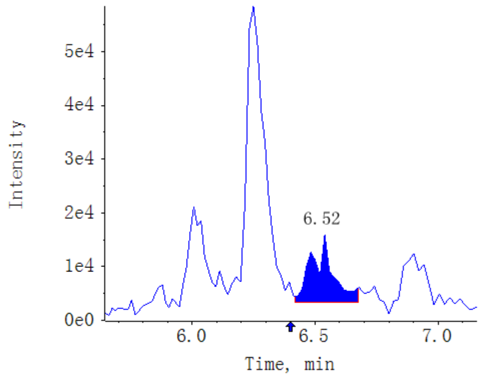

A20024805a_a
lut(C18:1) AREA:5.661e4 S/N:20.2

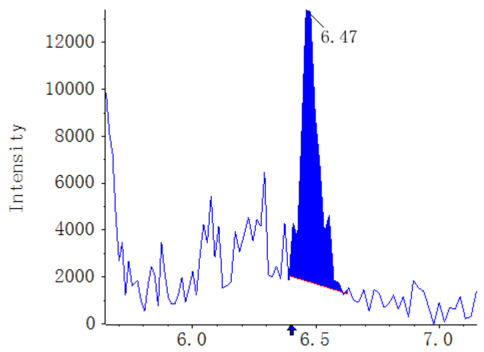

A20024805a_b
lut(C18:1) AREA:6.540e4 S/N:24.4

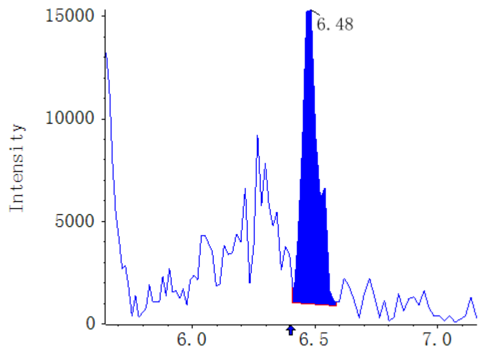

A20024808a_a
lut(C18:1) AREA:1.919e5 S/N:9.5

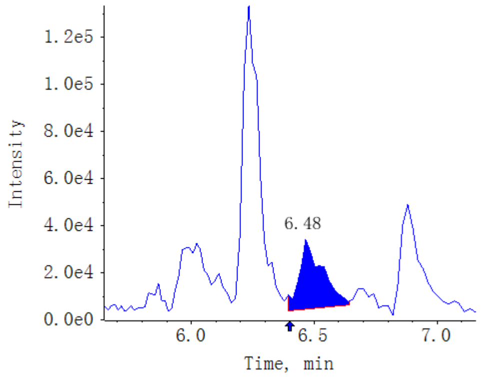

A20024808a_b
lut(C18:1) AREA:1.693e5 S/N:8.0

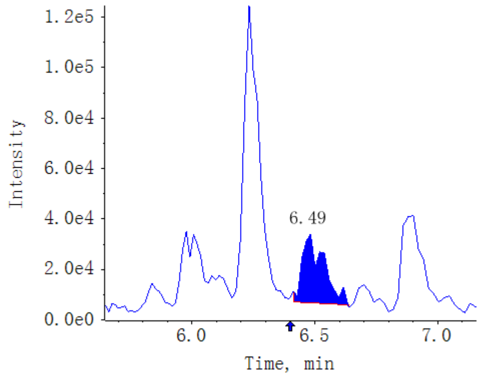

A20024811a_a
lut(C18:1) AREA:9.099e4 S/N:7.5

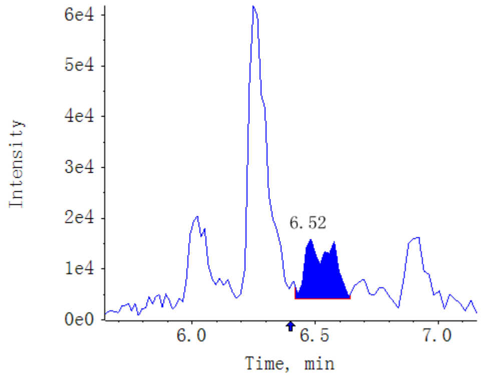

A20024811a_b
lut(C18:1) AREA:8.083e4 S/N:8.0

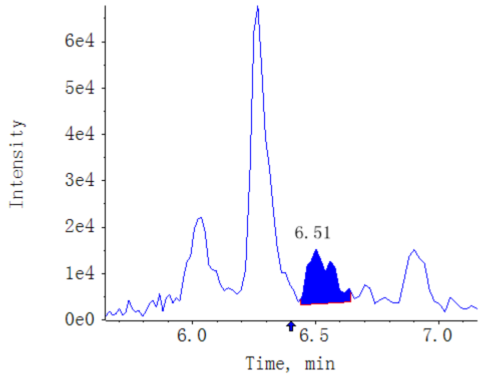

Compound name: neochrome palmitate
Regression Equation: y = 2.48212 x + -0.00832 (r = 0.99810) (weighting: 1 / x)

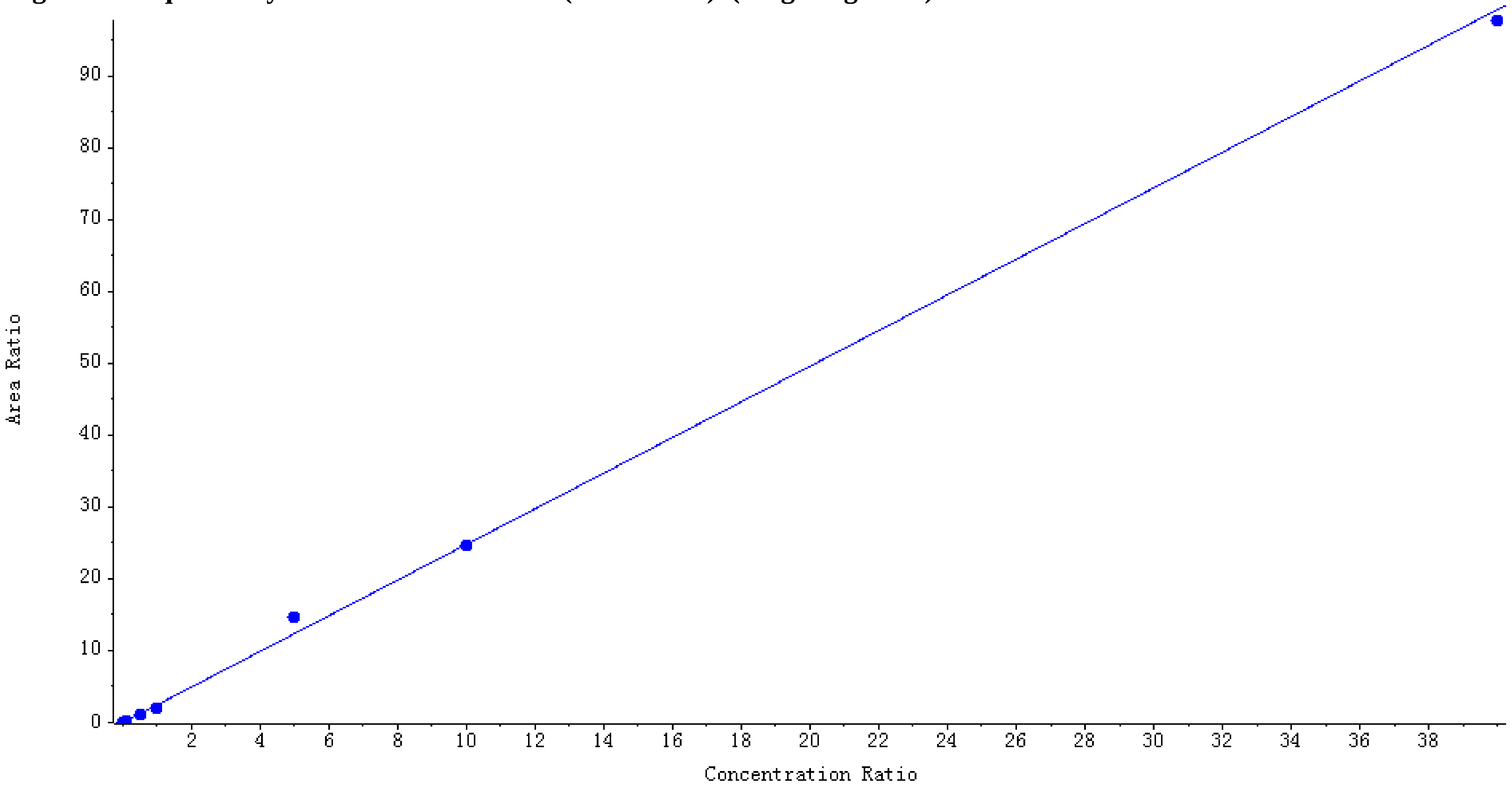

Peak Review

BLANK
neoc(C16:0) AREA:N/A S/N:N/A

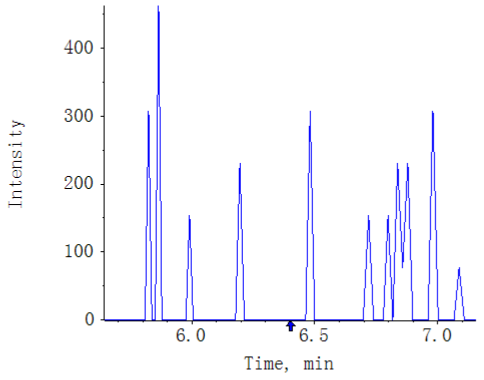

MWMS_20200904_1
neoc(C16:0) AREA:N/A S/N:N/A

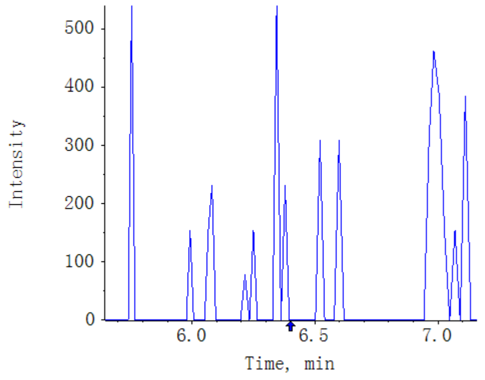

A20024797a_a
neoc(C16:0) AREA:N/A S/N:N/A

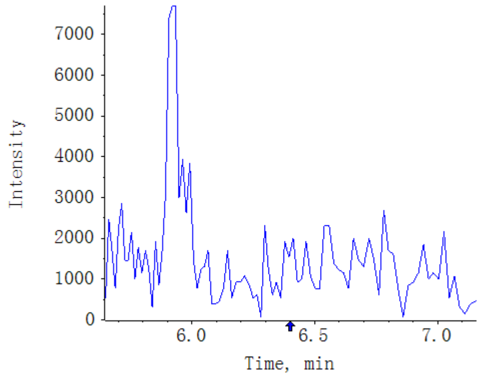

A20024797a_b
neoc(C16:0) AREA:N/A S/N:N/A

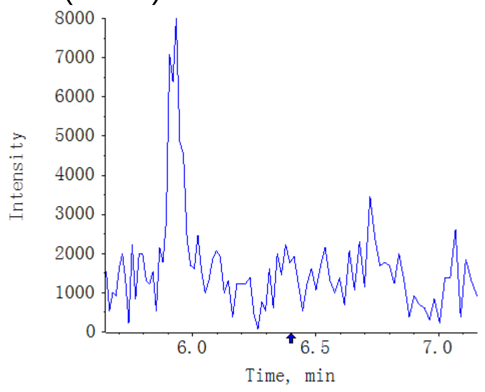

A20024800a_a
neoc(C16:0) AREA:1.530e5
S/N:15.5

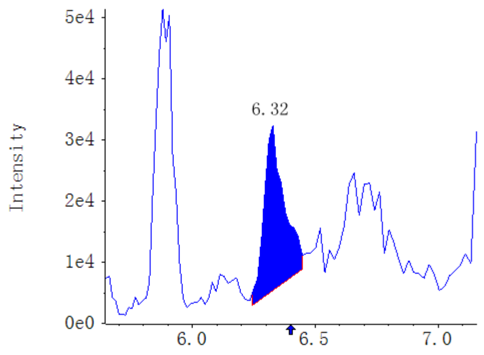

A20024800a_b
neoc(C16:0) AREA:1.334e5
S/N:14.7

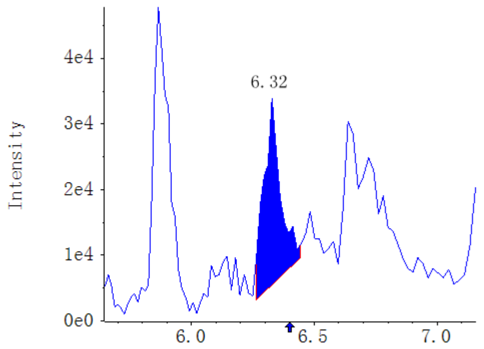

A20024802a_a
neoc(C16:0) AREA:2.308e5
S/N:21.2

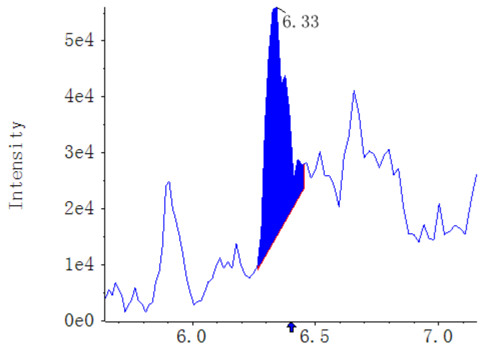

A20024802a_b
neoc(C16:0) AREA:2.274e5
S/N:18.1

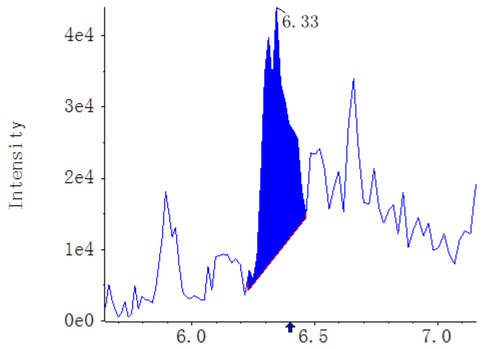

A20024805a_a
neoc(C16:0) AREA:N/A S/N:N/A

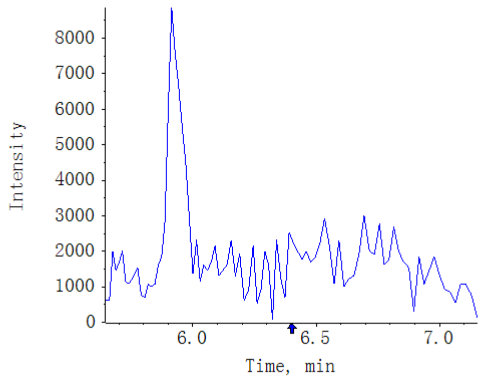

A20024805a_b
neoc(C16:0) AREA:N/A S/N:N/A

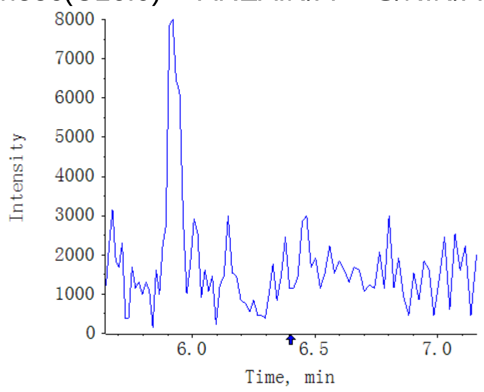

A20024808a_a
neoc(C16:0) AREA:1.049e5
S/N:9.4

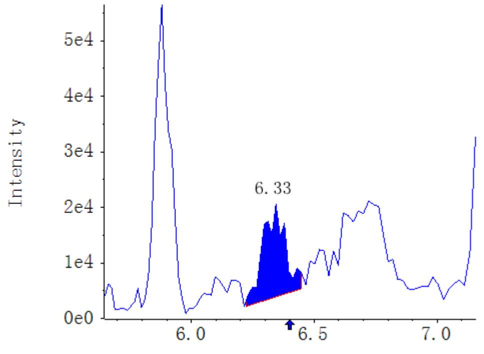

A20024808a_b
neoc(C16:0) AREA:9.580e4
S/N:8.2

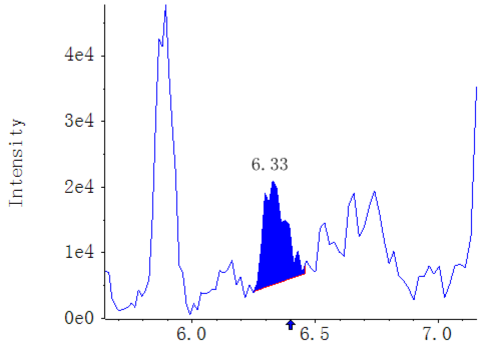

A20024811a_a
neoc(C16:0) AREA:1.086e5
S/N:18.4

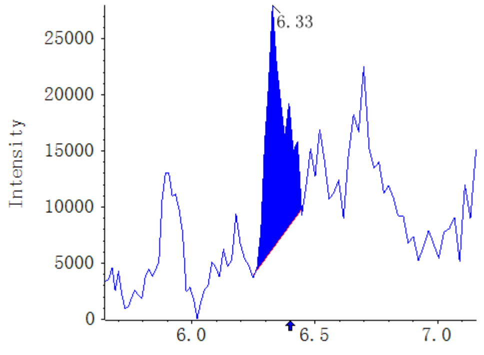

A20024811a_b
neoc(C16:0) AREA:1.004e5
S/N:14.4

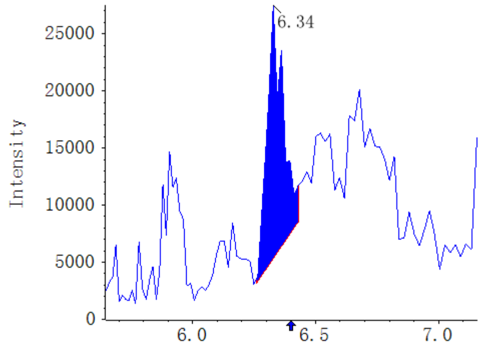

Compound name: lutein palmitate
Regression Equation: y = 0.62745 x + 0.00632 (r = 0.99819) (weighting: 1 / x)

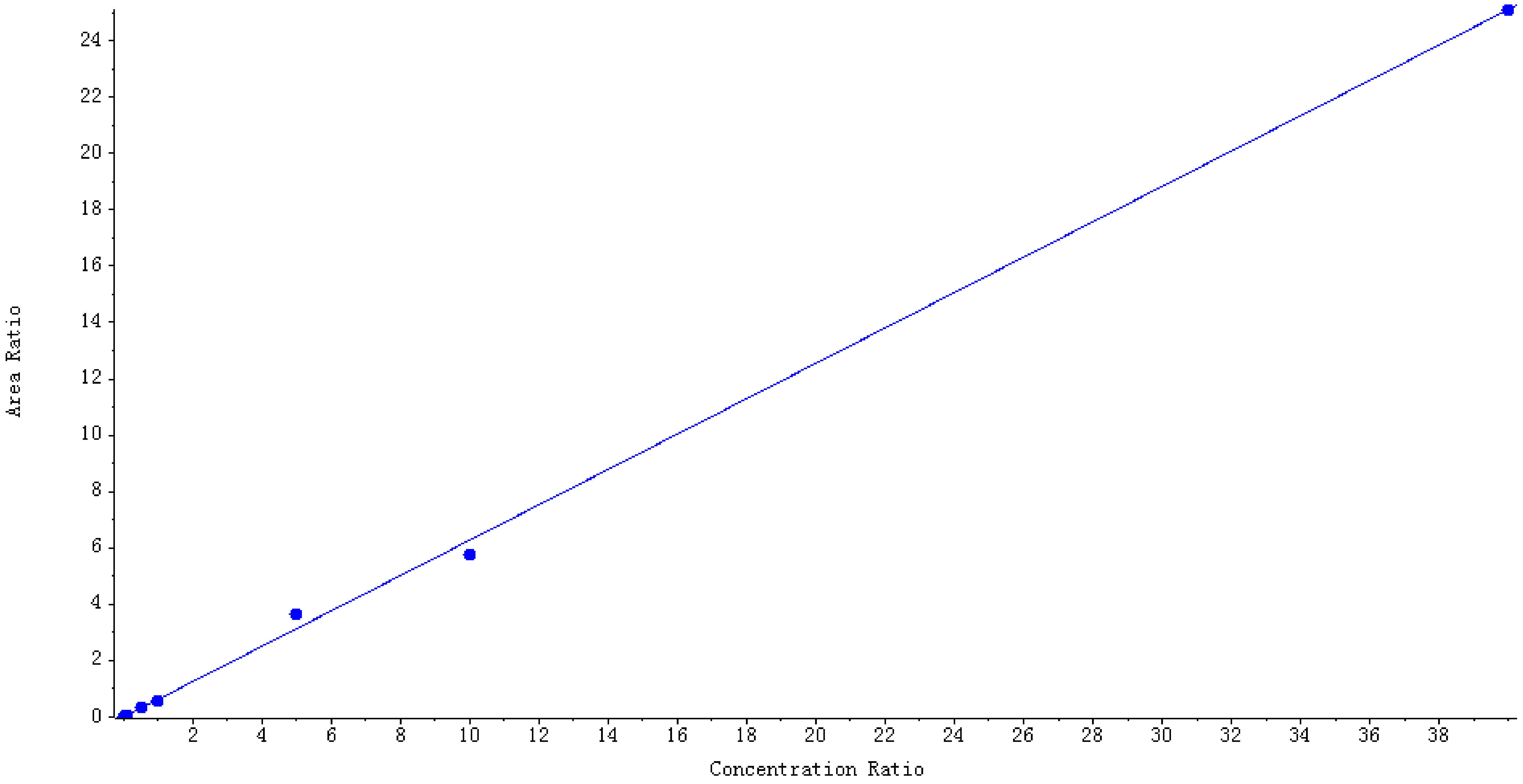

Peak Review

BLANK
rub(C16:0) AREA:N/A S/N:N/A

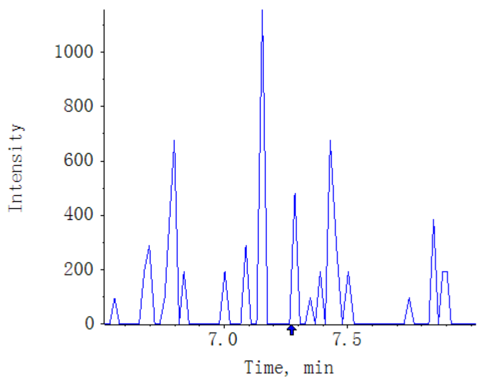

MWMS_20200904_1
rub(C16:0) AREA:N/A S/N:N/A

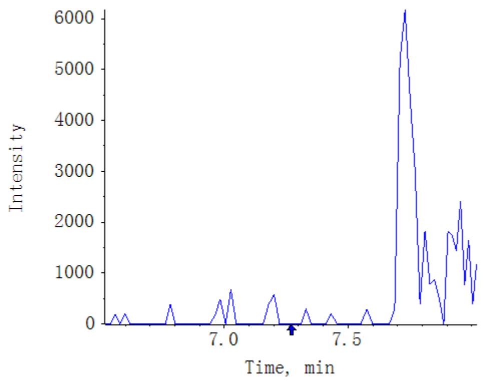

A20024797a_a
rub(C16:0) AREA:5.481e4 S/N:9.4

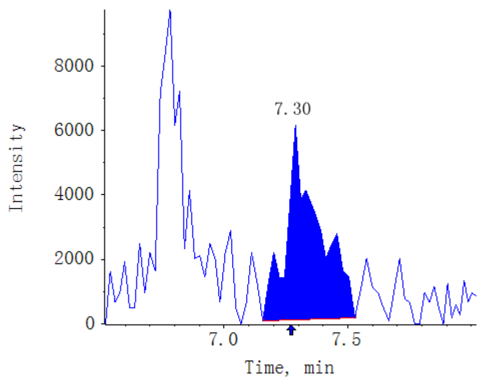

A20024797a_b
rub(C16:0) AREA:7.037e4
S/N:11.5

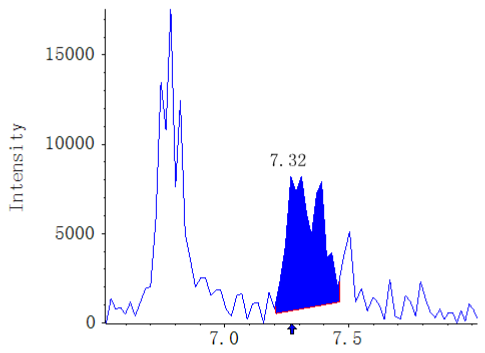

A20024800a_a
rub(C16:0) AREA:1.608e6
S/N:54.7

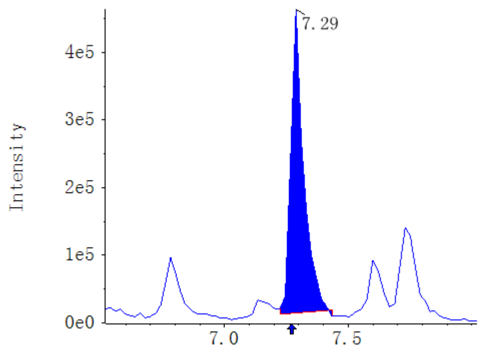

A20024800a_b
rub(C16:0) AREA:1.378e6
S/N:45.1

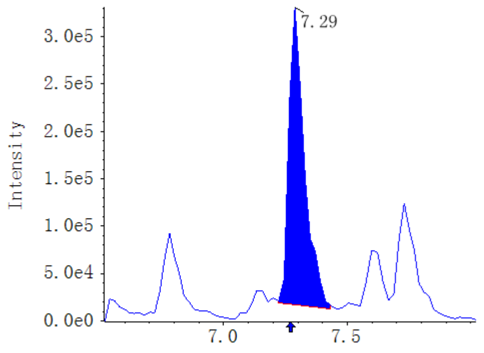

A20024802a_a
rub(C16:0) AREA:6.480e6
S/N:116.3

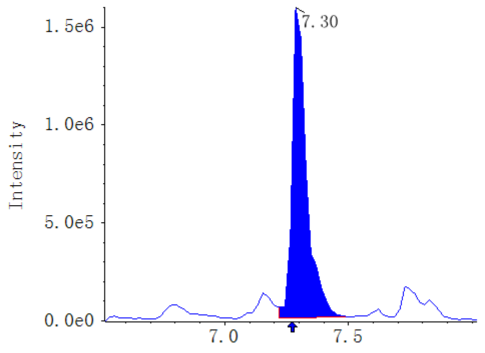

A20024802a_b
rub(C16:0) AREA:5.789e6
S/N:137.8

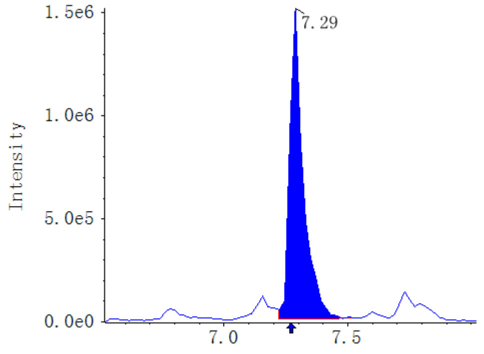

A20024805a_a
rub(C16:0) AREA:8.504e4
S/N:14.7

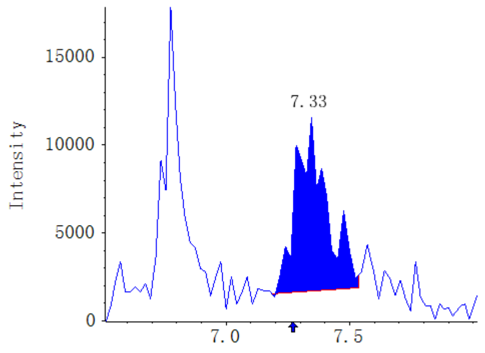

A20024805a_b
rub(C16:0) AREA:8.088e4
S/N:15.6

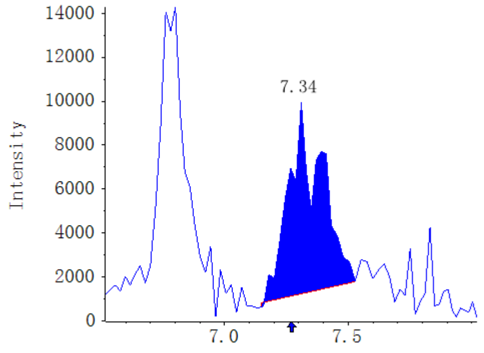

A20024808a_a
rub(C16:0) AREA:3.951e5
S/N:21.6

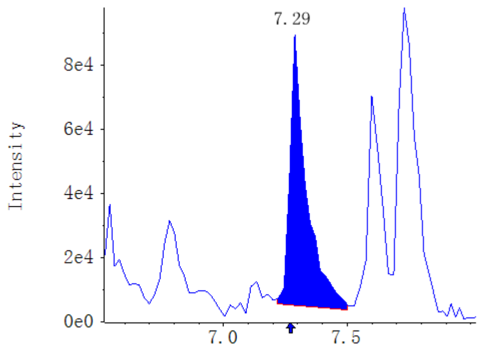

A20024808a_b
rub(C16:0) AREA:3.146e5
S/N:21.8

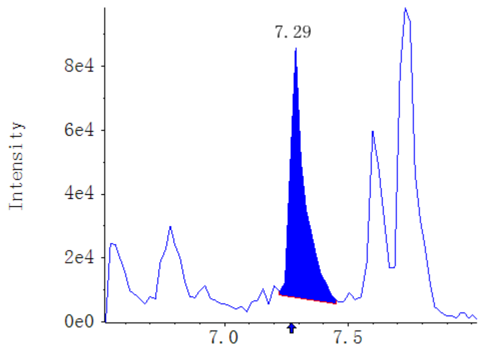

A20024811a_a
rub(C16:0) AREA:1.903e6
S/N:94.3

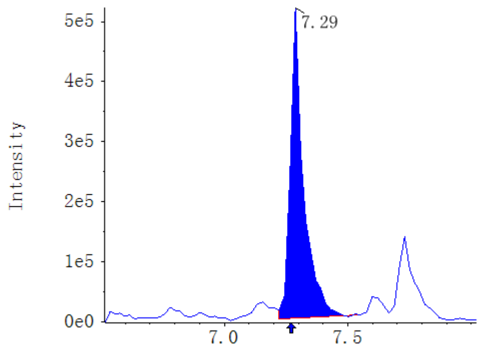

A20024811a_b
rub(C16:0) AREA:1.655e6
S/N:89.1

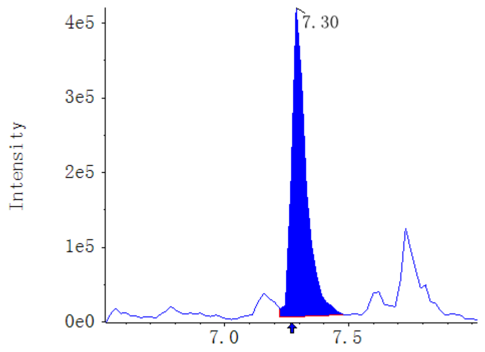

Compound name: violaxanthin myristate
Regression Equation: y = 3.55083 x + 9.23931e-4 (r = 0.99411) (weighting: 1 / x)

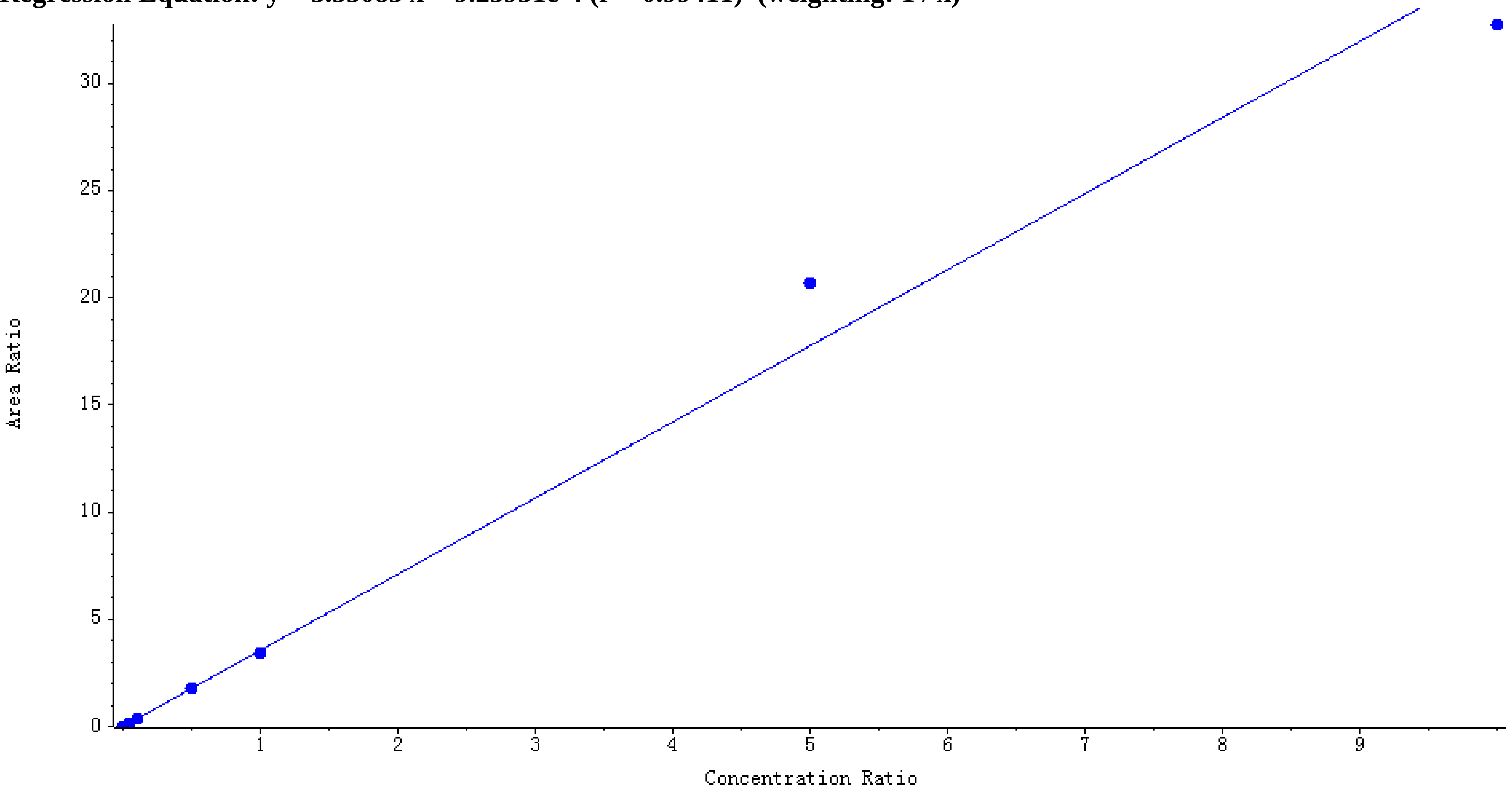

Peak Review

BLANK
vio(C14:0) AREA:N/A S/N:N/A

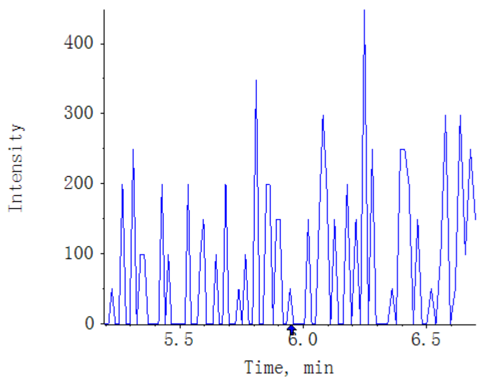

MWMS_20200904_1
vio(C14:0) AREA:N/A S/N:N/A

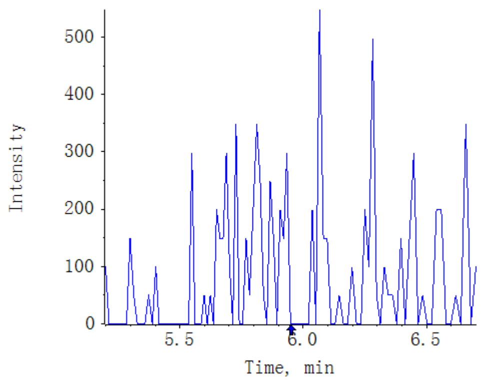

A20024797a_a
vio(C14:0) AREA:N/A S/N:N/A

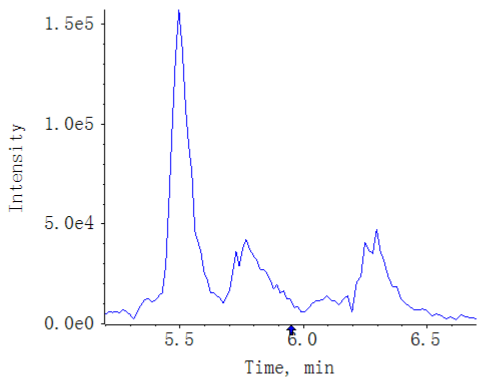

A20024797a_b
vio(C14:0) AREA:N/A S/N:N/A

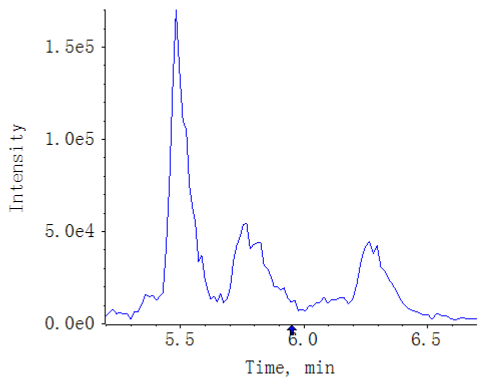

A20024800a_a
vio(C14:0) AREA:6.010e5
S/N:27.9

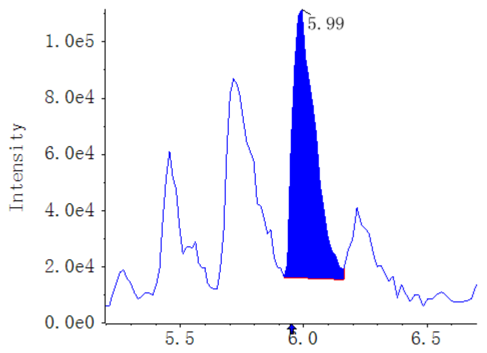

A20024800a_b
vio(C14:0) AREA:5.574e5
S/N:23.7

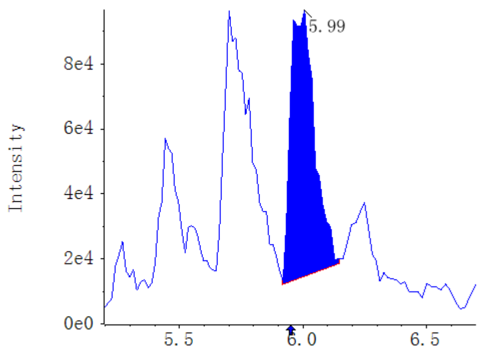

A20024802a_a
vio(C14:0) AREA:4.295e5
S/N:31.2

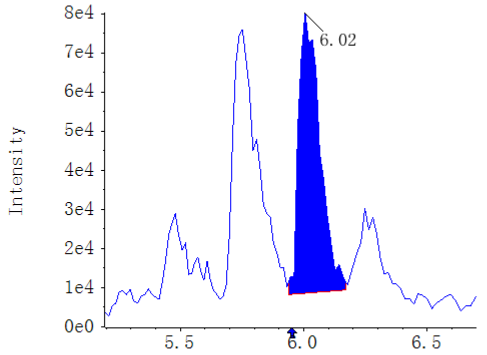

A20024802a_b
vio(C14:0) AREA:4.283e5
S/N:38.9

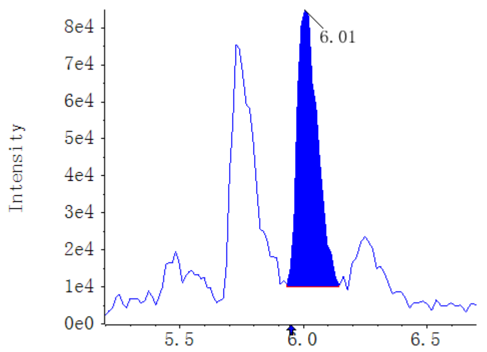

A20024805a_a
vio(C14:0) AREA:N/A S/N:N/A

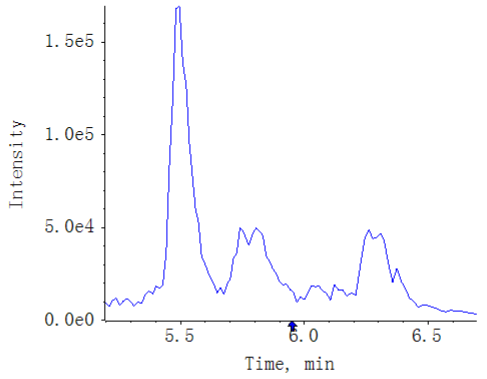

A20024805a_b
vio(C14:0) AREA:N/A S/N:N/A

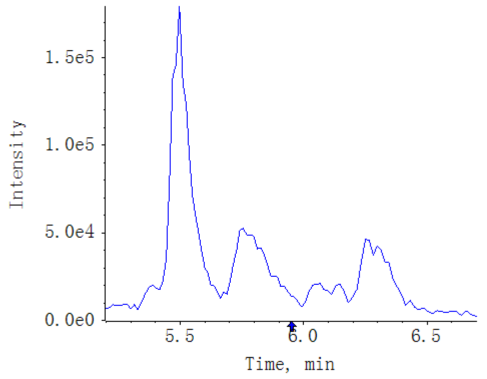

A20024808a_a
vio(C14:0) AREA:8.209e5
S/N:34.8

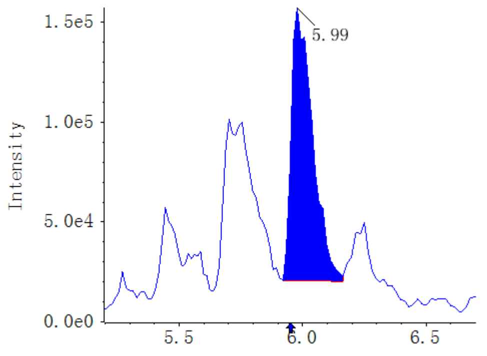

A20024808a_b
vio(C14:0) AREA:8.387e5
S/N:34.0

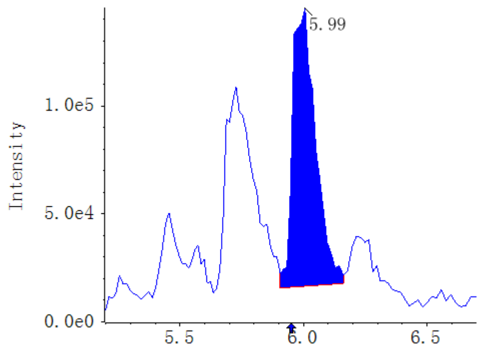

A20024811a_a
vio(C14:0) AREA:5.190e5
S/N:45.8

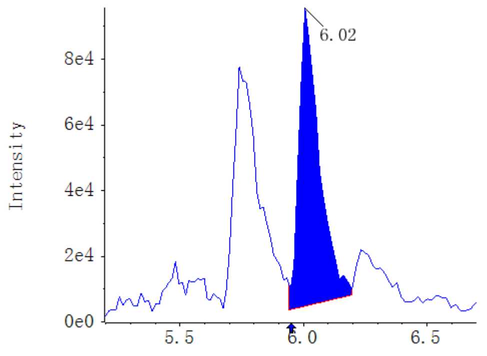

A20024811a_b
vio(C14:0) AREA:4.151e5
S/N:44.2

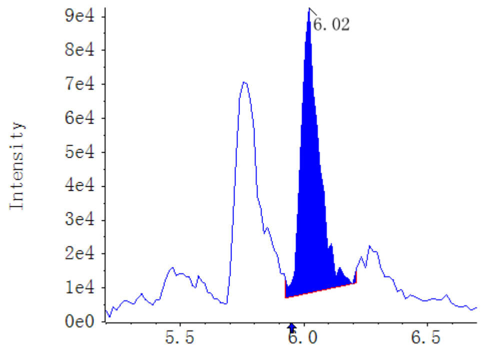

Compound name: violaxanthin palmitate
Regression Equation: y = 3.55083 x + 9.23931e-4 (r = 0.99411) (weighting: 1 / x)

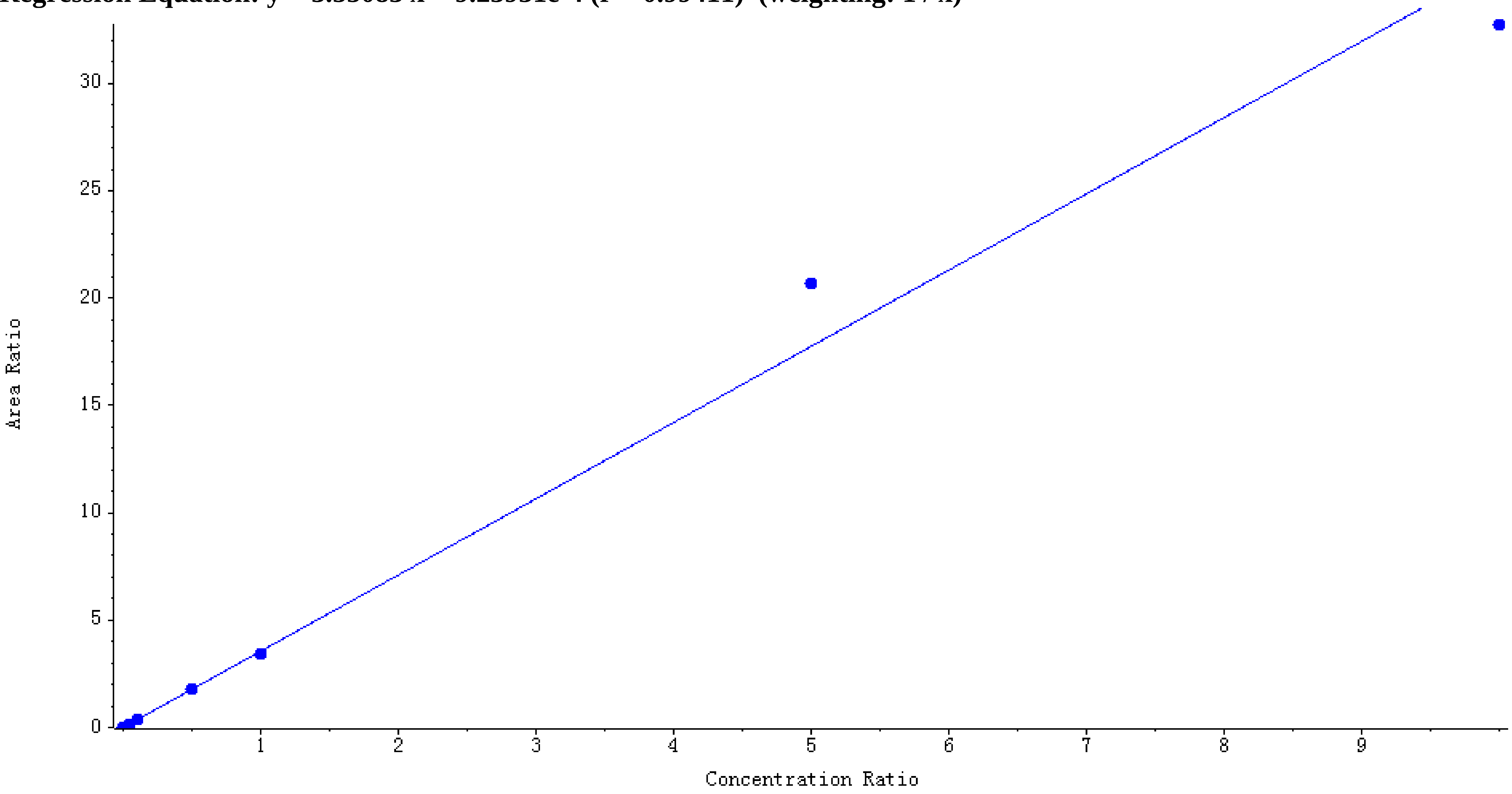

Peak Review

BLANK
vio(C16:0) AREA:N/A S/N:N/A

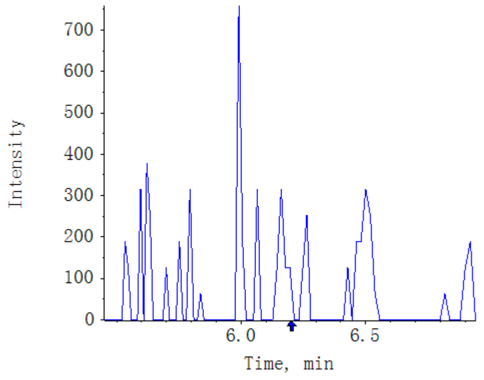

MWMS_20200904_1
vio(C16:0) AREA:N/A S/N:N/A

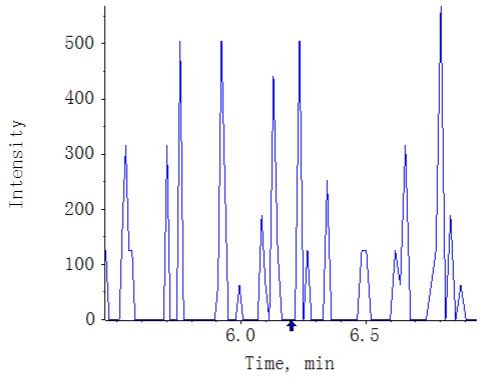

A20024797a_a
vio(C16:0) AREA:4.963e5
S/N:24.0

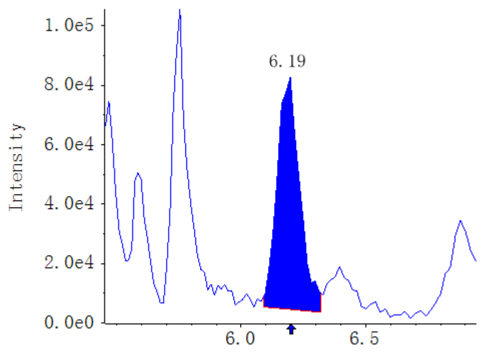

A20024797a_b
vio(C16:0) AREA:5.580e5
S/N:24.9

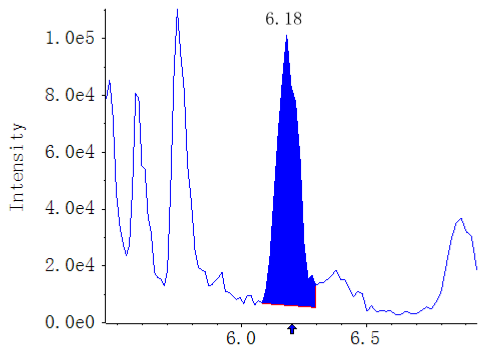

A20024800a_a
vio(C16:0) AREA:4.228e5
S/N:29.8

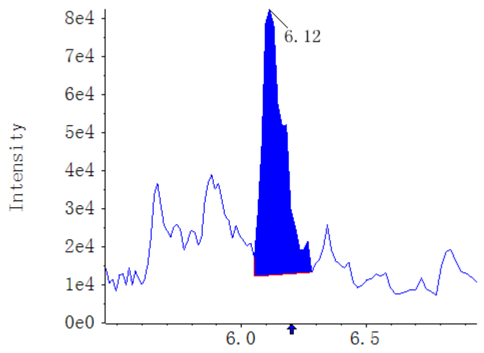

A20024800a_b
vio(C16:0) AREA:3.067e5
S/N:21.4

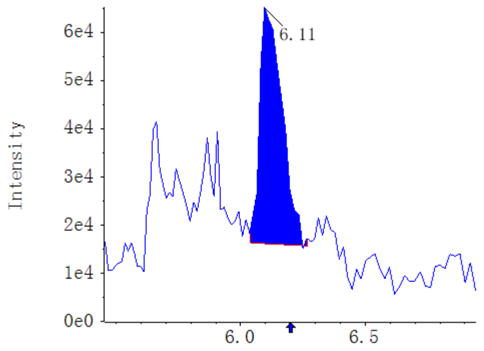

A20024802a_a
vio(C16:0) AREA:1.185e5
S/N:12.4

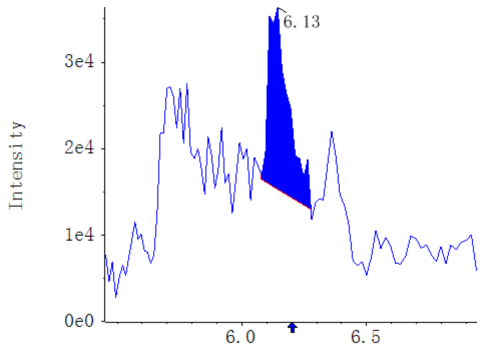

A20024802a_b
vio(C16:0) AREA:9.330e4
S/N:14.0

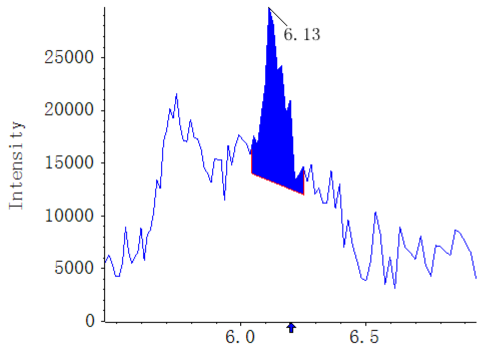

A20024805a_a
vio(C16:0) AREA:6.324e5
S/N:20.7

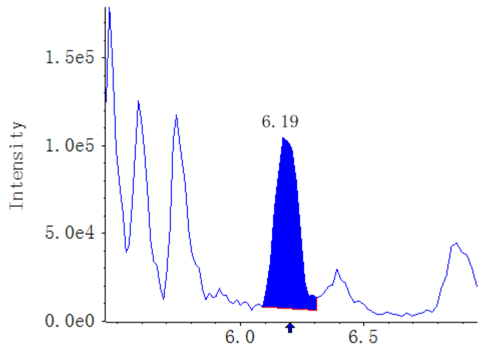

A20024805a_b
vio(C16:0) AREA:5.105e5
S/N:19.8

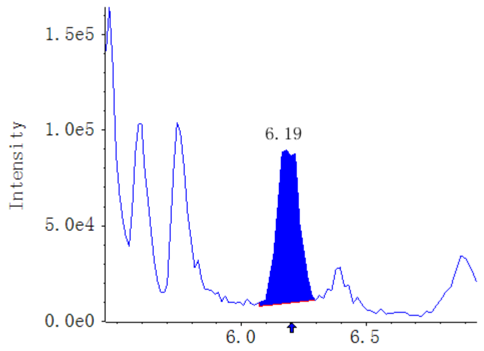

A20024808a_a
vio(C16:0) AREA:3.931e5
S/N:23.9

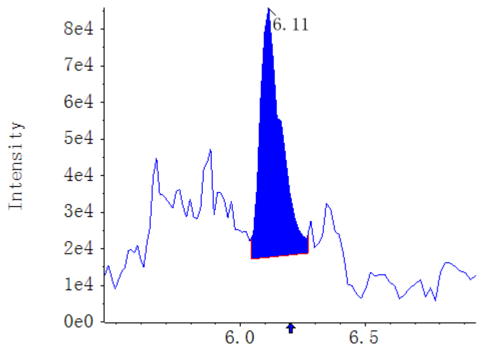

A20024808a_b
vio(C16:0) AREA:3.661e5
S/N:22.7

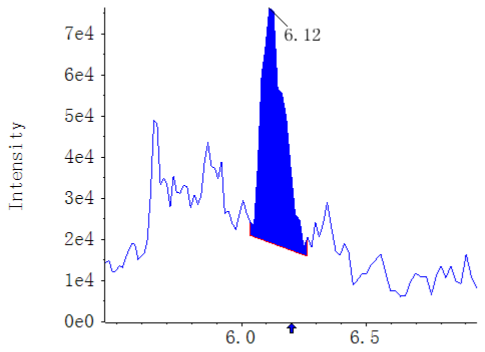

A20024811a_a
vio(C16:0) AREA:1.265e5
S/N:14.7

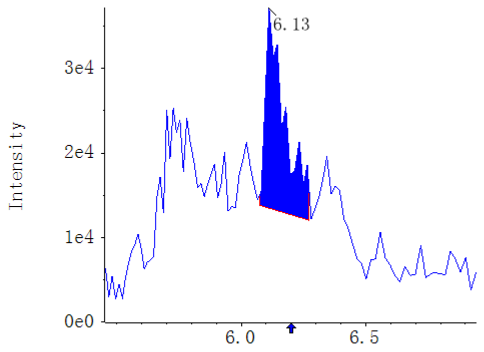

A20024811a_b
vio(C16:0) AREA:1.067e5
S/N:15.4

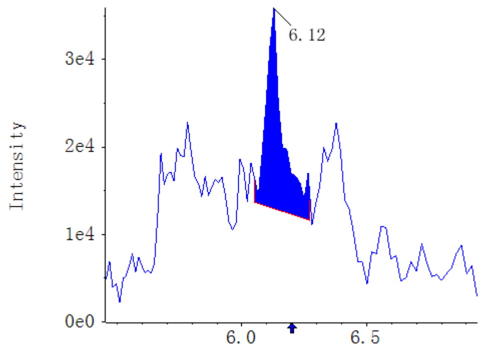

Compound name: violaxanthin dipalmitate
Regression Equation: y = 3.55083 x + 9.23931e-4 (r = 0.99411) (weighting: 1 / x)

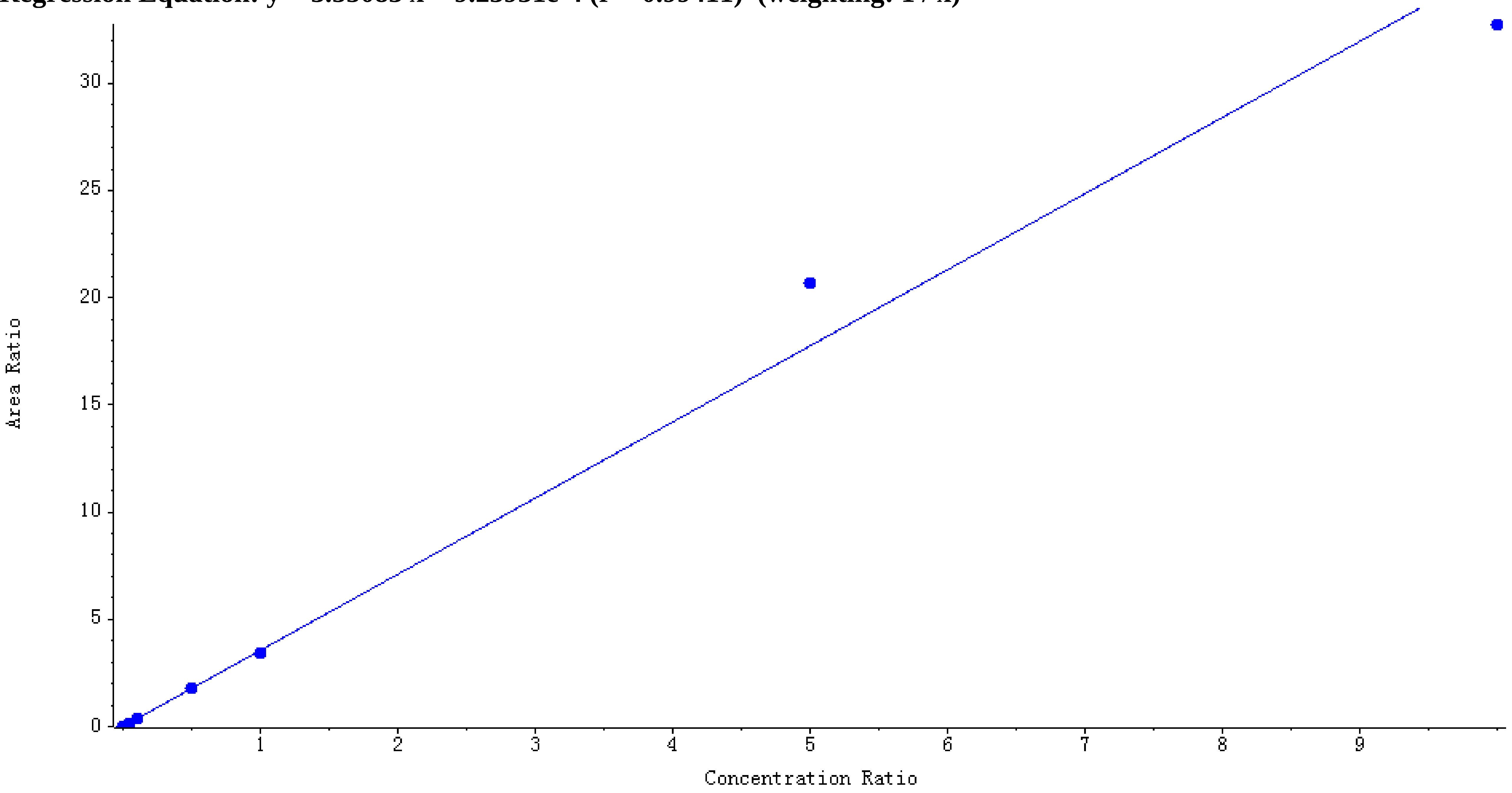

Peak Review

BLANK
vio(C16:0/C16:0) AREA:N/A
S/N:N/A

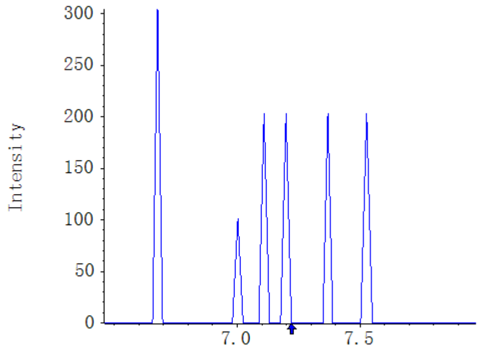

MWMS_20200904_1
vio(C16:0/C16:0) AREA:N/A
S/N:N/A

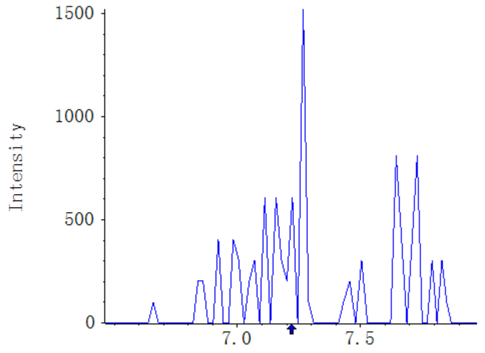

A20024797a_a
vio(C16:0/C16:0) AREA:N/A
S/N:N/A

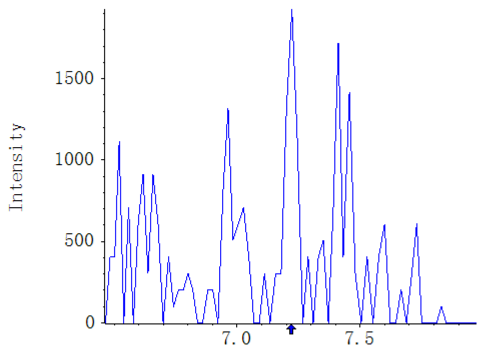

A20024797a_b
vio(C16:0/C16:0) AREA:N/A
S/N:N/A

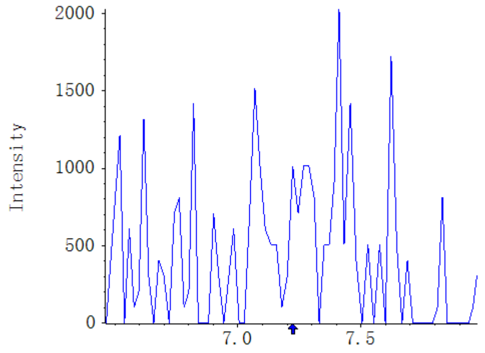

A20024800a_a
vio(C16:0/C16:0) AREA:1.292e6
S/N:78.2

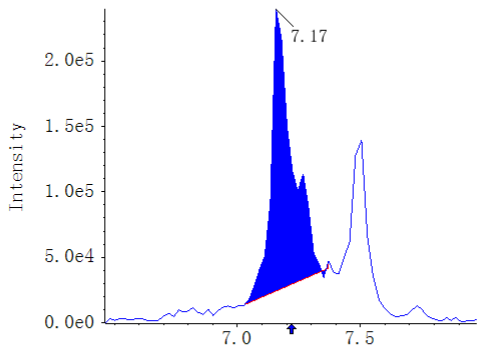

A20024800a_b
vio(C16:0/C16:0) AREA:1.096e6
S/N:60.3

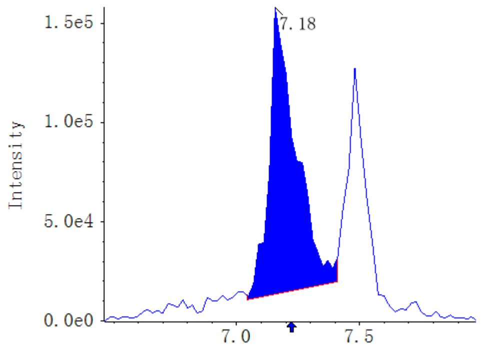

A20024802a_a
vio(C16:0/C16:0) AREA:4.587e5
S/N:19.1

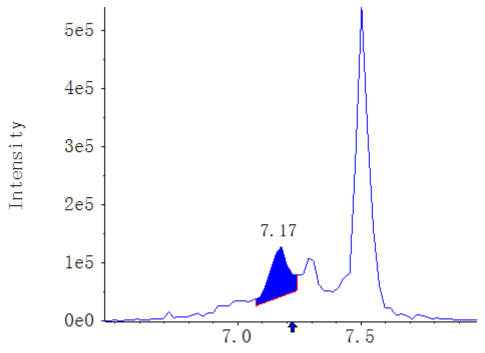

A20024802a_b
vio(C16:0/C16:0) AREA:3.498e5
S/N:16.9

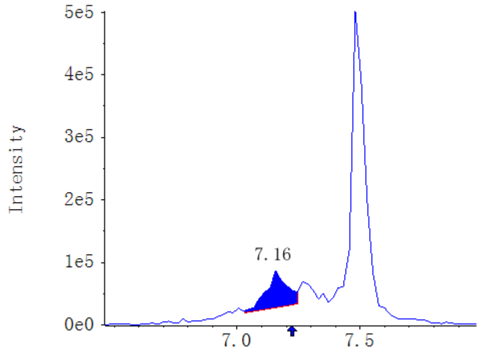

A20024805a_a
vio(C16:0/C16:0) AREA:N/A
S/N:N/A

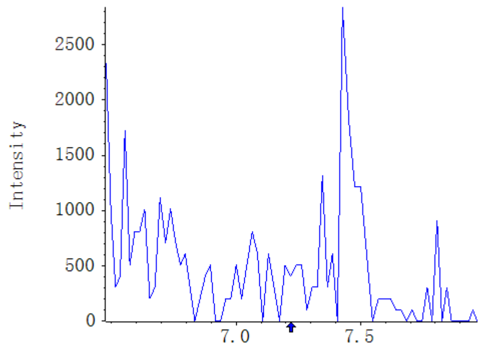

A20024805a_b
vio(C16:0/C16:0) AREA:N/A
S/N:N/A

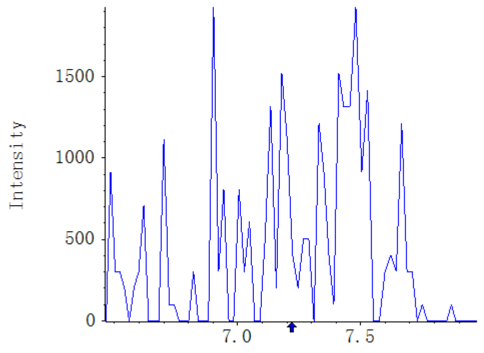

A20024808a_a
vio(C16:0/C16:0) AREA:1.929e6
S/N:106.7

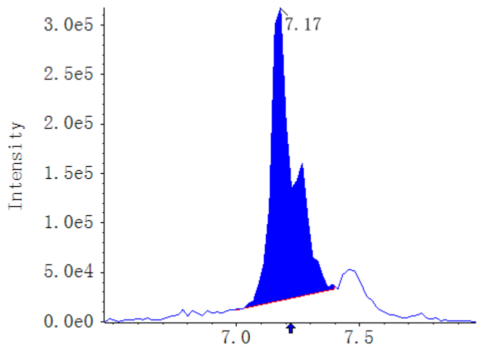

A20024808a_b
vio(C16:0/C16:0) AREA:1.870e6
S/N:123.0

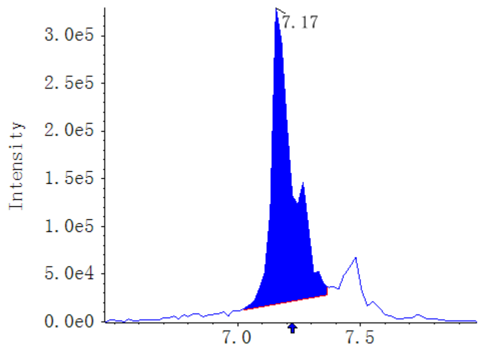

A20024811a_a
vio(C16:0/C16:0) AREA:7.032e5
S/N:34.1

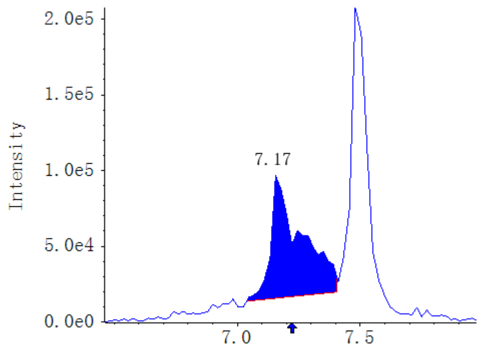

A20024811a_b
vio(C16:0/C16:0) AREA:6.347e5
S/N:26.3

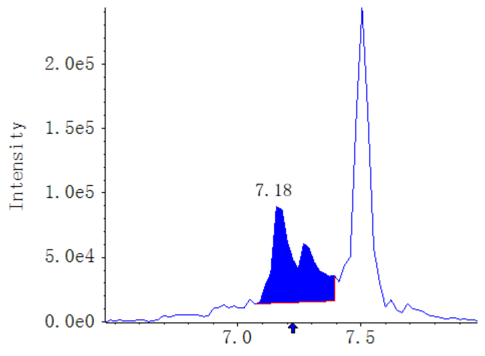

Compound name: zeaxanthin palmitate

Regression Equation: y = 0.19627 x + 0.00647 (r = 0.99741) (weighting: 1 / x)

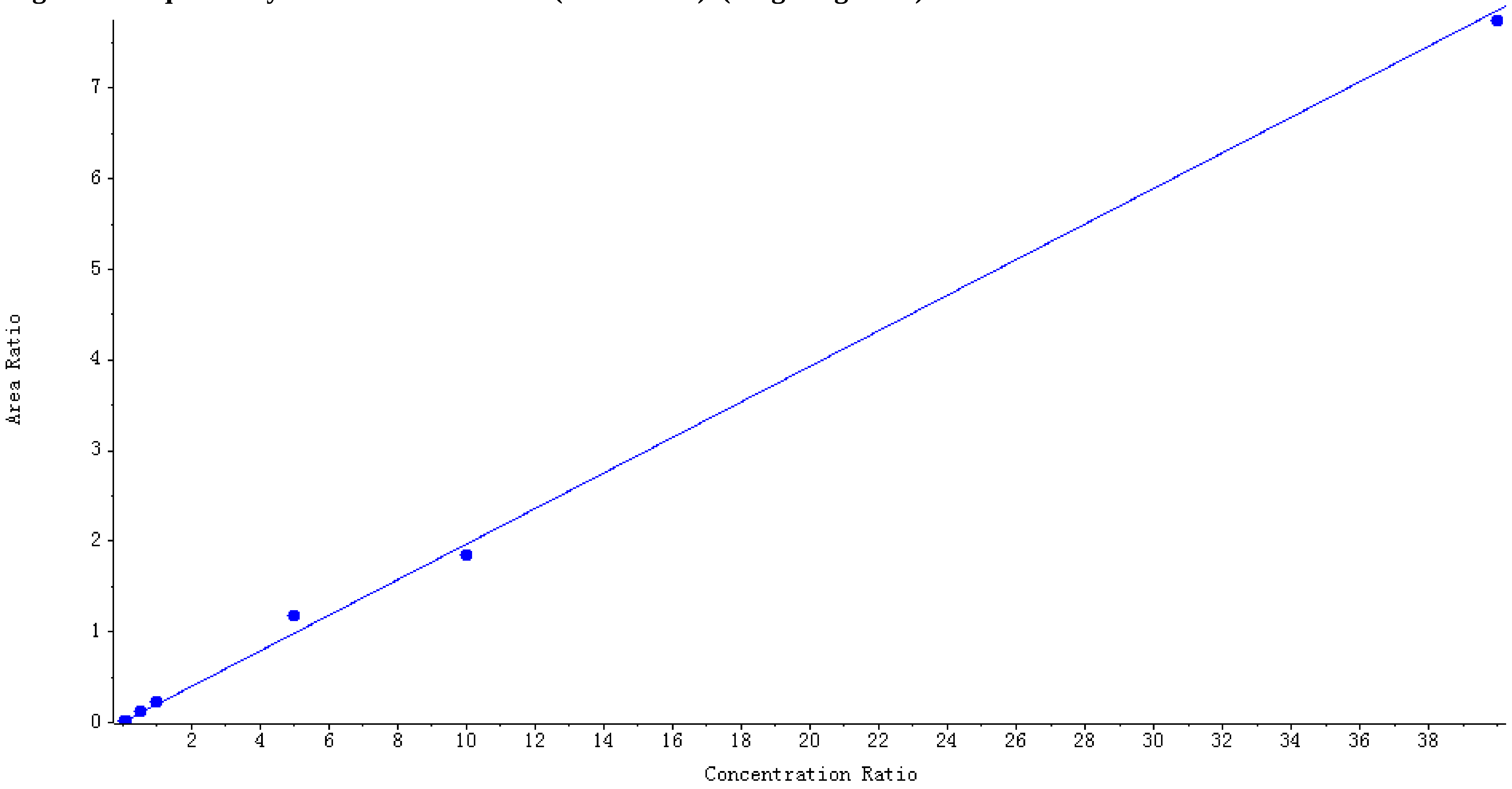

Peak Review

BLANK
zea(C16:0) AREA:N/A S/N:N/A

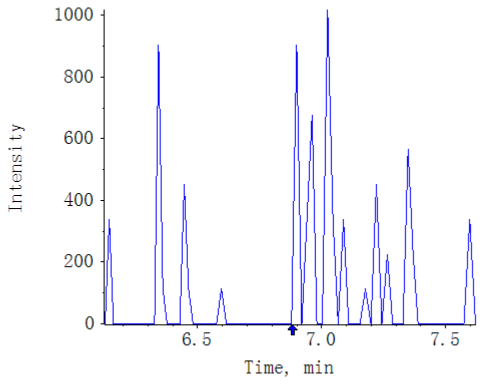

MWMS_20200904_1
zea(C16:0) AREA:N/A S/N:N/A

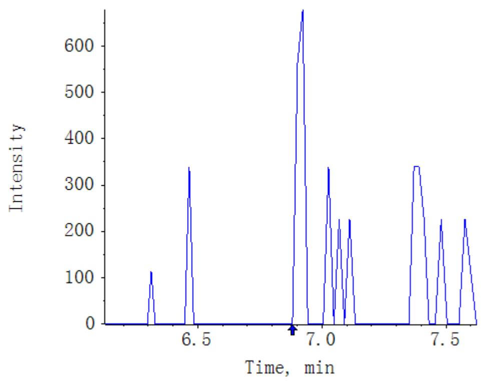

A20024797a_a
zea(C16:0) AREA:N/A S/N:N/A

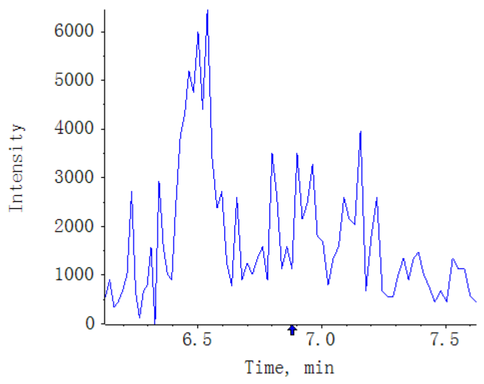

A20024797a_b
zea(C16:0) AREA:N/A S/N:N/A

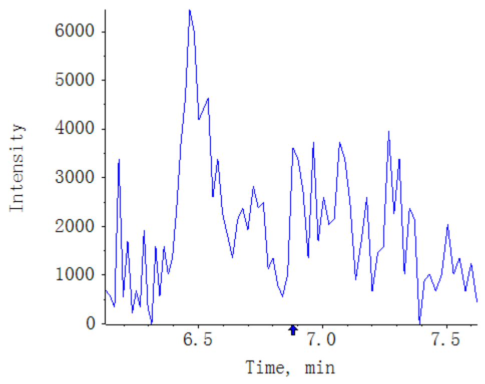

A20024800a_a
zea(C16:0) AREA:1.114e6
S/N:64.4

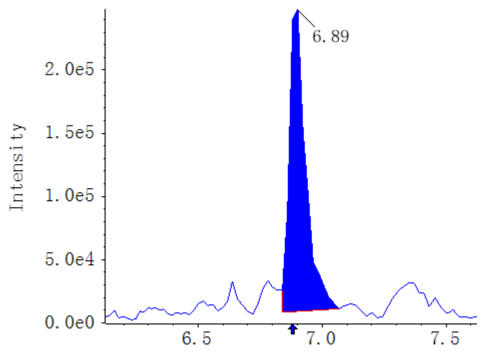

A20024800a_b
zea(C16:0) AREA:1.038e6
S/N:68.1

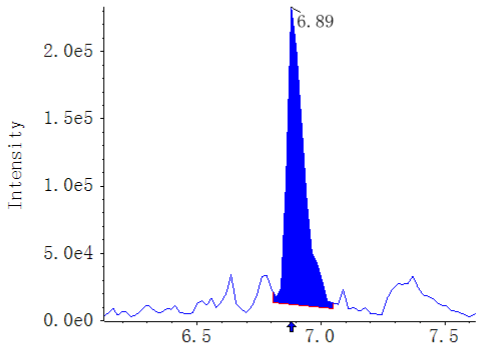

A20024802a_a
zea(C16:0) AREA:5.053e6
S/N:139.8

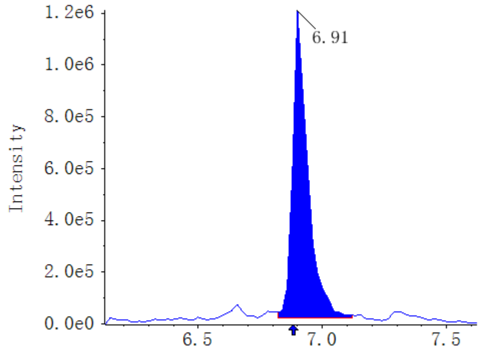

A20024802a_b
zea(C16:0) AREA:2.714e6
S/N:113.1

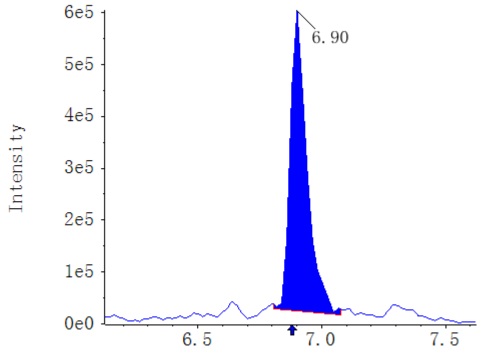

A20024805a_a
zea(C16:0) AREA:N/A S/N:N/A

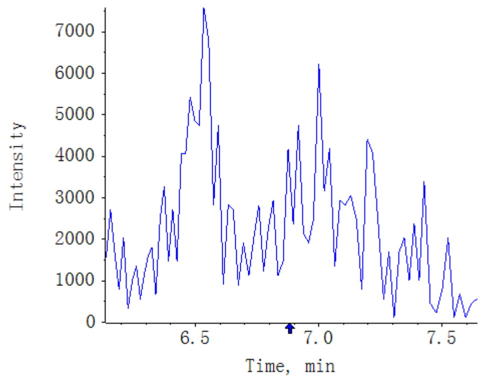

A20024805a_b
zea(C16:0) AREA:N/A S/N:N/A

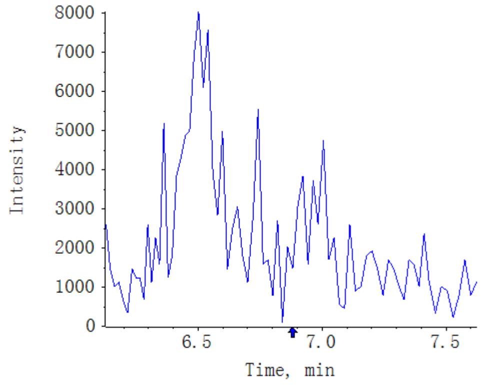

A20024808a_a
zea(C16:0) AREA:3.204e5
S/N:25.4

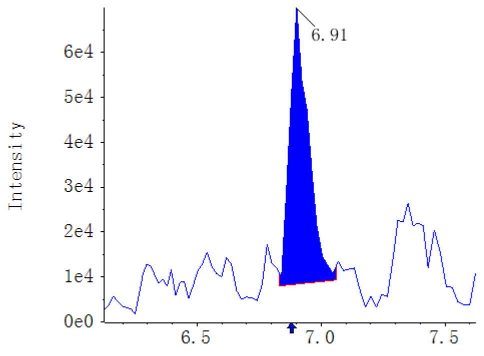

A20024808a_b
zea(C16:0) AREA:2.929e5
S/N:22.1

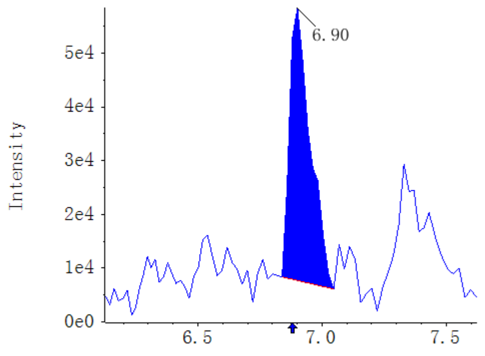

A20024811a_a
zea(C16:0) AREA:7.514e5
S/N:54.1

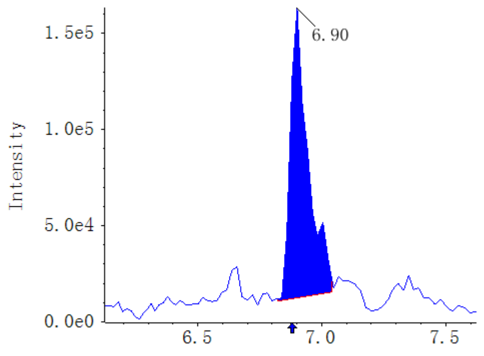

A20024811a_b
zea(C16:0) AREA:7.657e5
S/N:50.4

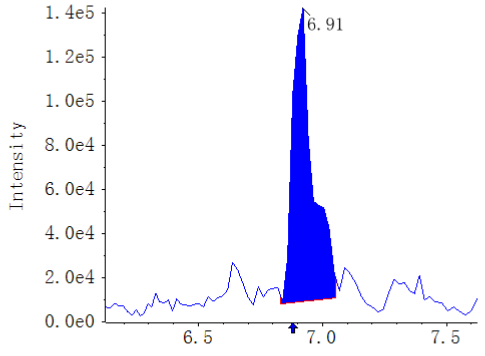

Compound name: zeaxanthin dimyristate
Regression Equation: y = 0.19627 x + 0.00647 (r = 0.99741) (weighting: 1 / x)

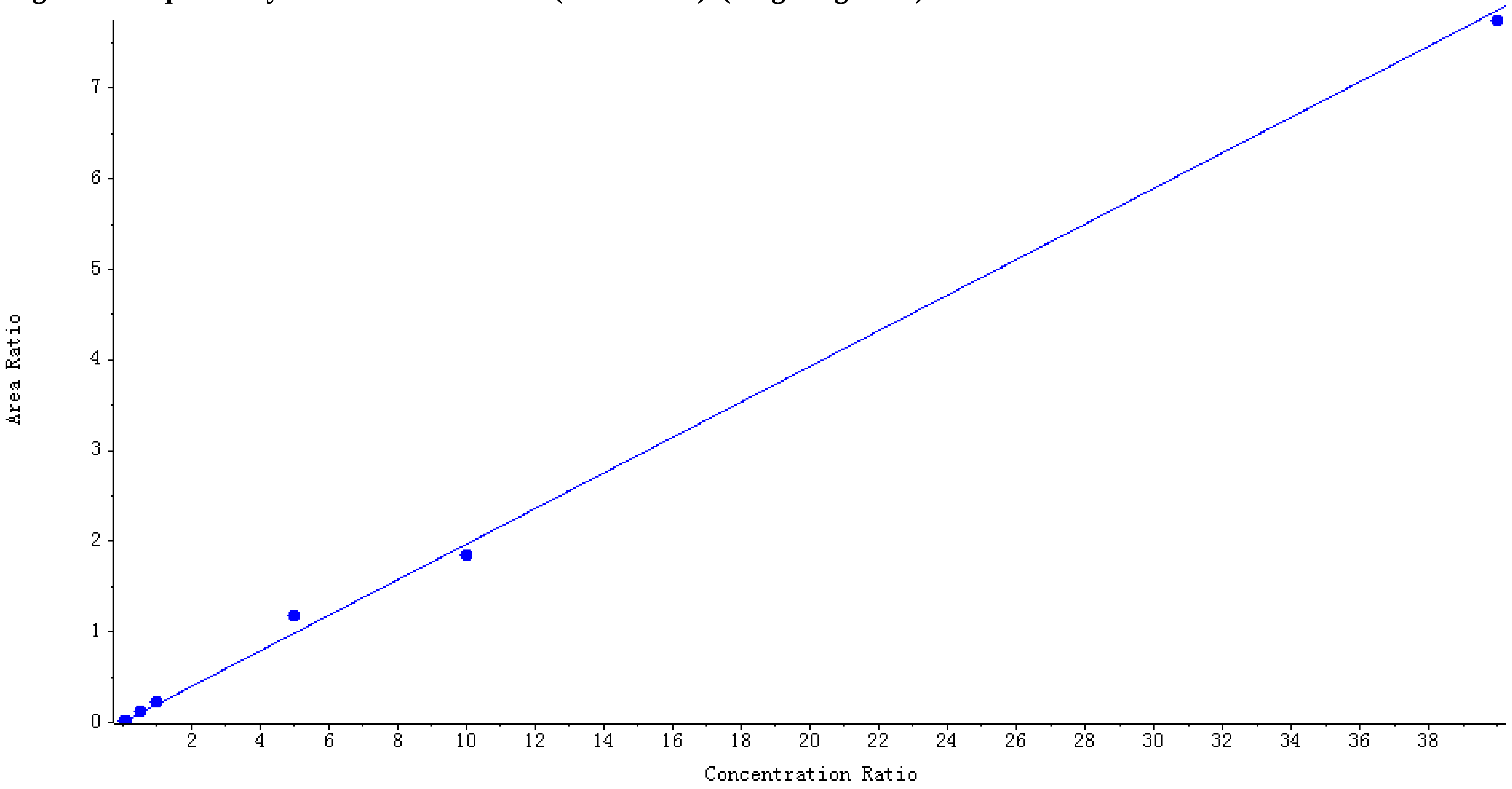

Peak Review

BLANK
zea(C14:0/C14:0) AREA:N/A
S/N:N/A

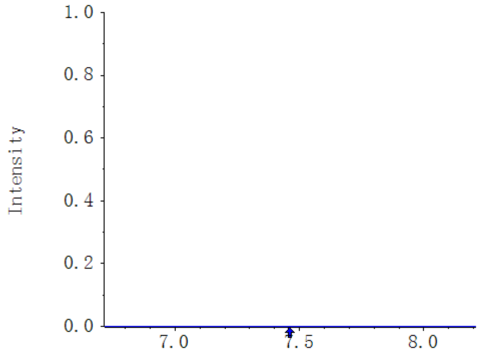

MWMS_20200904_1
zea(C14:0/C14:0) AREA:N/A
S/N:N/A

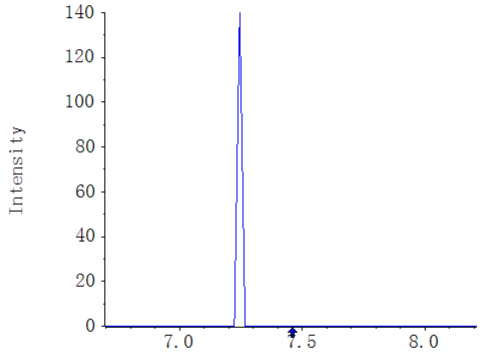

A20024797a_a
zea(C14:0/C14:0) AREA:N/A
S/N:N/A

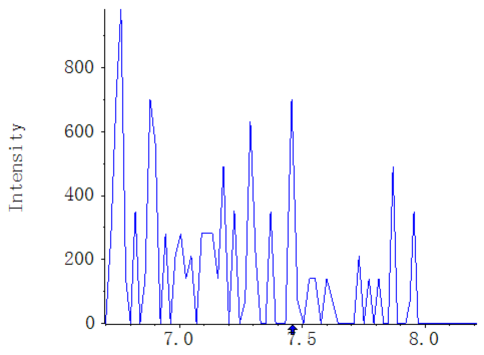

A20024797a_b
zea(C14:0/C14:0) AREA:N/A
S/N:N/A

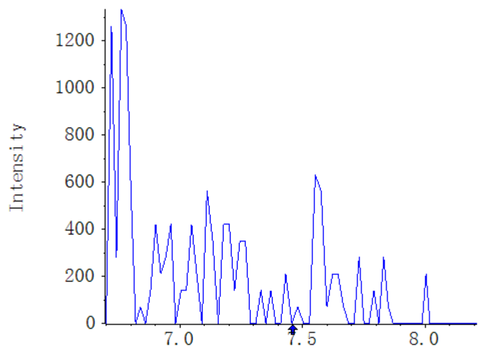

A20024800a_a
zea(C14:0/C14:0) AREA:1.284e5
S/N:163.6

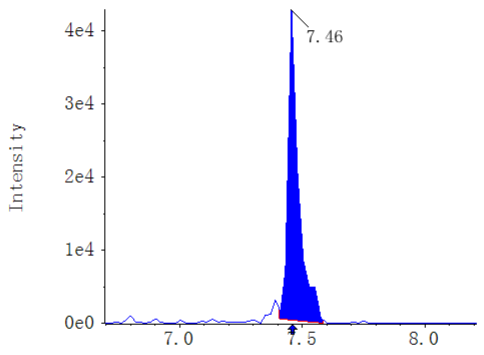

A20024800a_b
zea(C14:0/C14:0) AREA:1.145e5
S/N:131.3

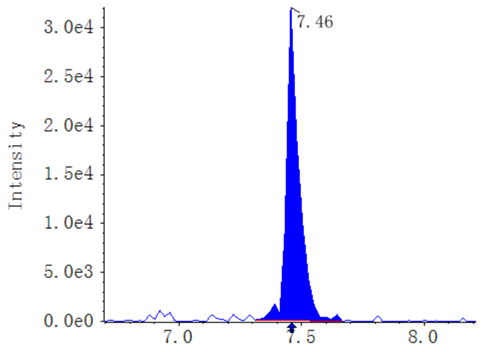

A20024802a_a
zea(C14:0/C14:0) AREA:8.173e4
S/N:169.9

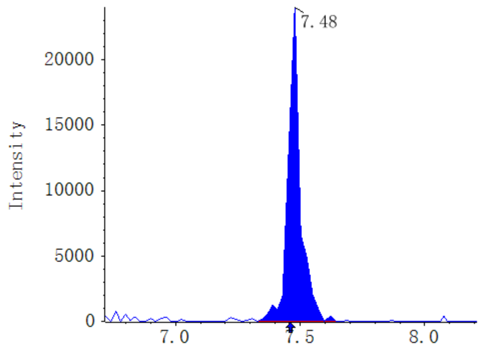

A20024802a_b
zea(C14:0/C14:0) AREA:7.721e4
S/N:75.4

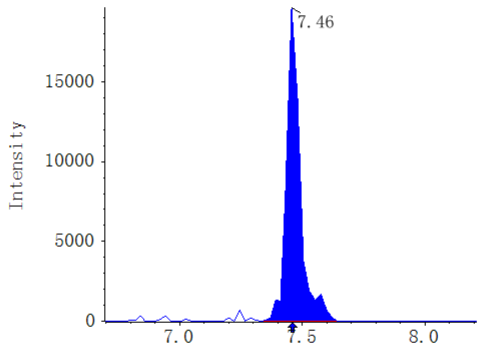

A20024805a_a
zea(C14:0/C14:0) AREA:N/A
S/N:N/A

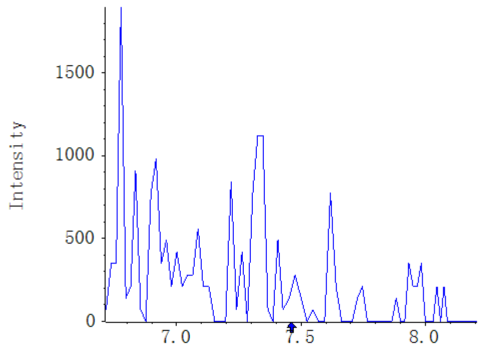

A20024805a_b
zea(C14:0/C14:0) AREA:N/A
S/N:N/A

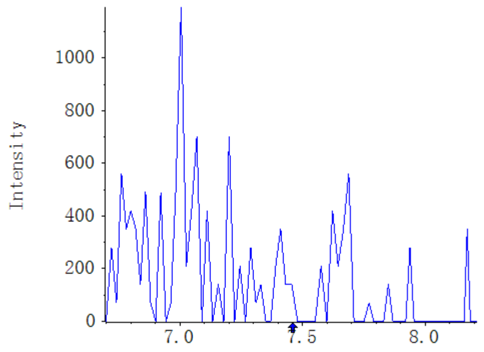

A20024808a_a
zea(C14:0/C14:0) AREA:2.568e4
S/N:52.8

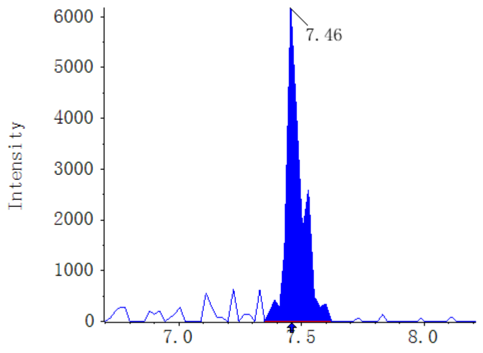

A20024808a_b
zea(C14:0/C14:0) AREA:2.084e4
S/N:38.8

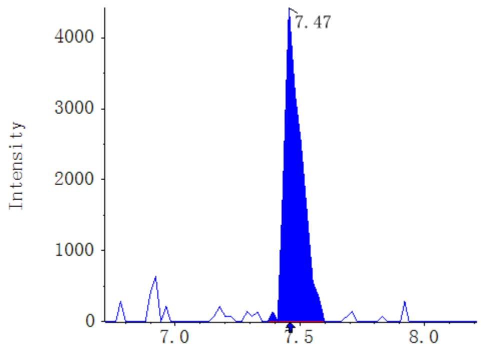

A20024811a_a
zea(C14:0/C14:0) AREA:3.117e4
S/N:64.8

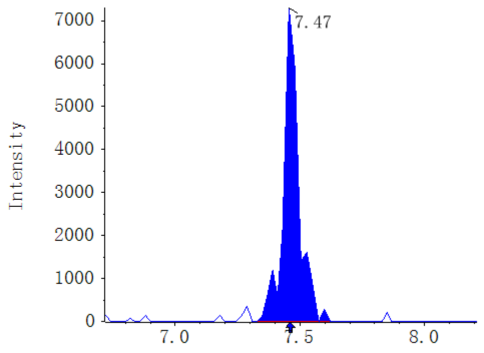

A20024811a_b
zea(C14:0/C14:0) AREA:3.482e4
S/N:48.0

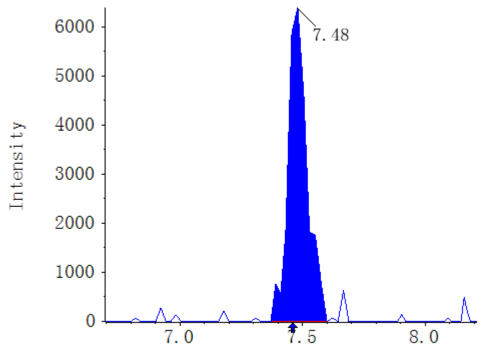

Compound name: zeaxanthin-laurate-palmitate

Regression Equation: y = 0.19627 x + 0.00647 (r = 0.99741) (weighting: 1 / x)

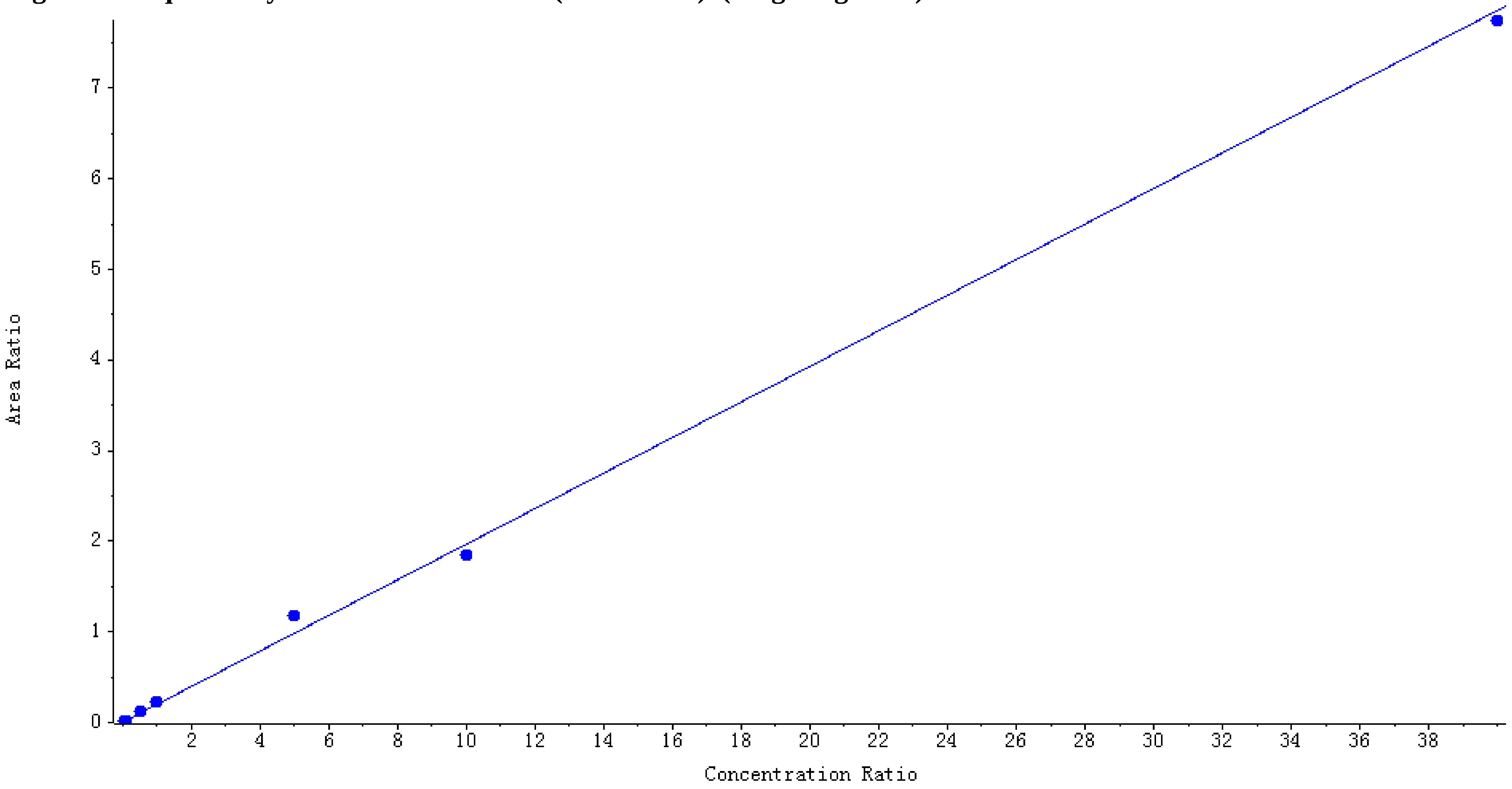

Peak Review

BLANK
zea(C12:0/C16:0) AREA:N/A
S/N:N/A

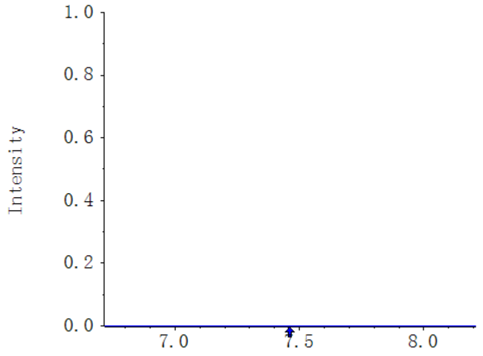

MWMS_20200904_1
zea(C12:0/C16:0) AREA:N/A
S/N:N/A

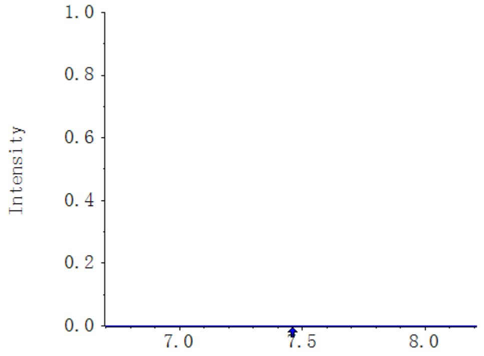

A20024797a_a
zea(C12:0/C16:0) AREA:N/A
S/N:N/A

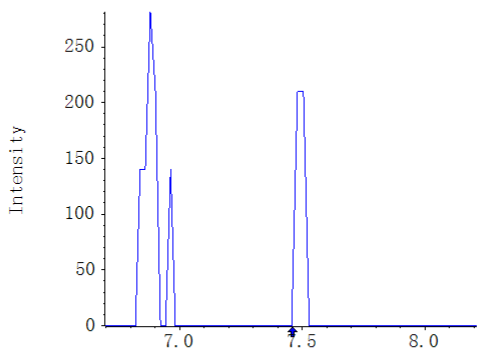

A20024797a_b
zea(C12:0/C16:0) AREA:N/A
S/N:N/A

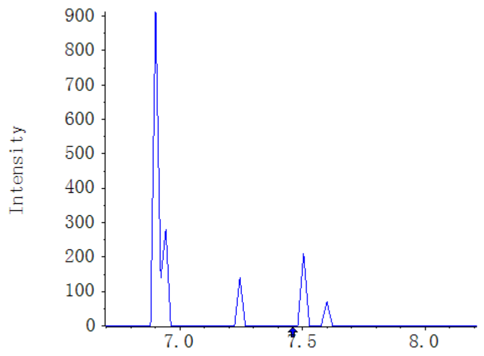

A20024800a_a
zea(C12:0/C16:0) AREA:4.457e4
S/N:45.5

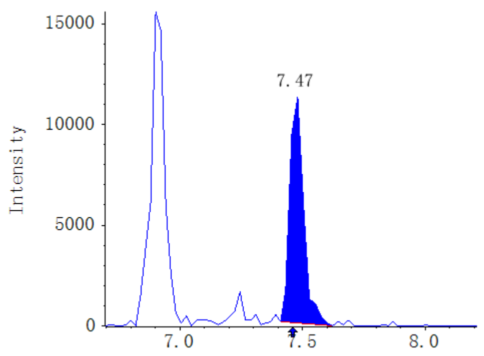

A20024800a_b
zea(C12:0/C16:0) AREA:3.557e4
S/N:39.7

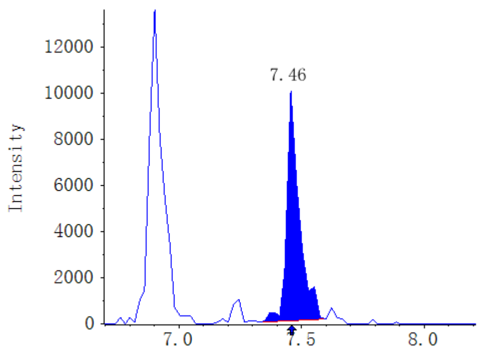

A20024802a_a
zea(C12:0/C16:0) AREA:2.909e4
S/N:32.7

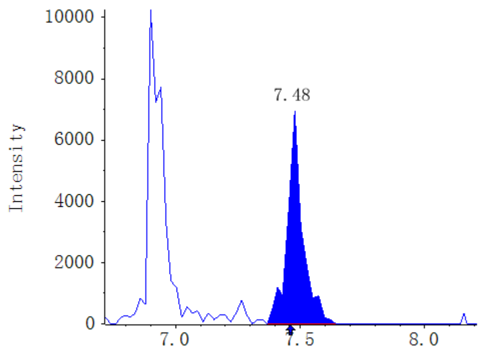

A20024802a_b
zea(C12:0/C16:0) AREA:2.201e4
S/N:33.7

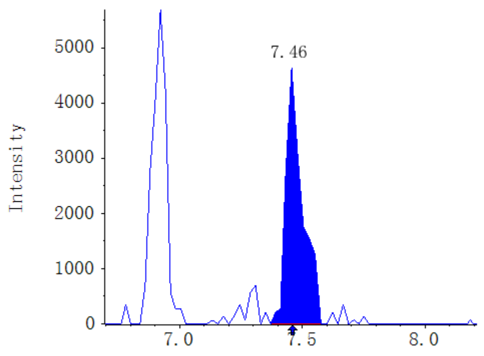

A20024805a_a
zea(C12:0/C16:0) AREA:N/A
S/N:N/A

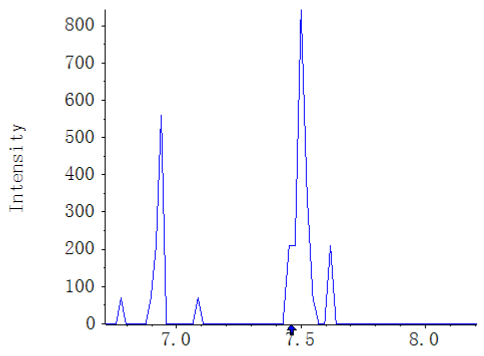

A20024805a_b
zea(C12:0/C16:0) AREA:N/A
S/N:N/A

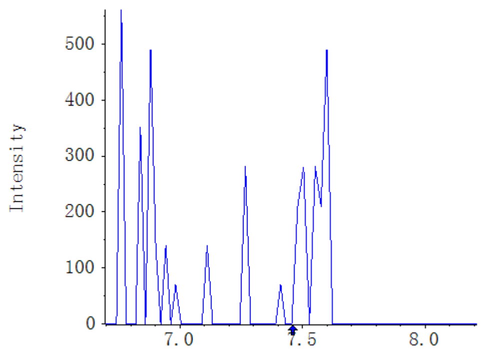

A20024808a_a
zea(C12:0/C16:0) AREA:8.814e3
S/N:12.2

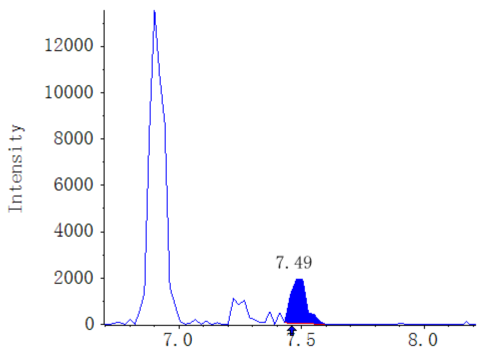

A20024808a_b
zea(C12:0/C16:0) AREA:8.185e3
S/N:13.6

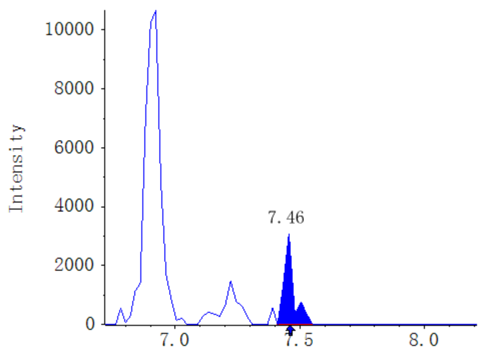

A20024811a_a
zea(C12:0/C16:0) AREA:1.141e4
S/N:19.9

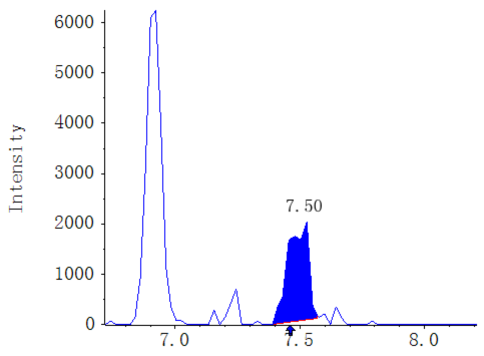

A20024811a_b
zea(C12:0/C16:0) AREA:1.004e4
S/N:21.9

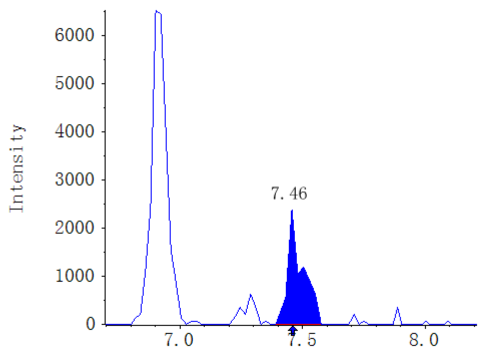

Compound name: zeaxanthin-myristate-palmitate
Regression Equation: y = 0.19627 x + 0.00647 (r = 0.99741) (weighting: 1 / x)

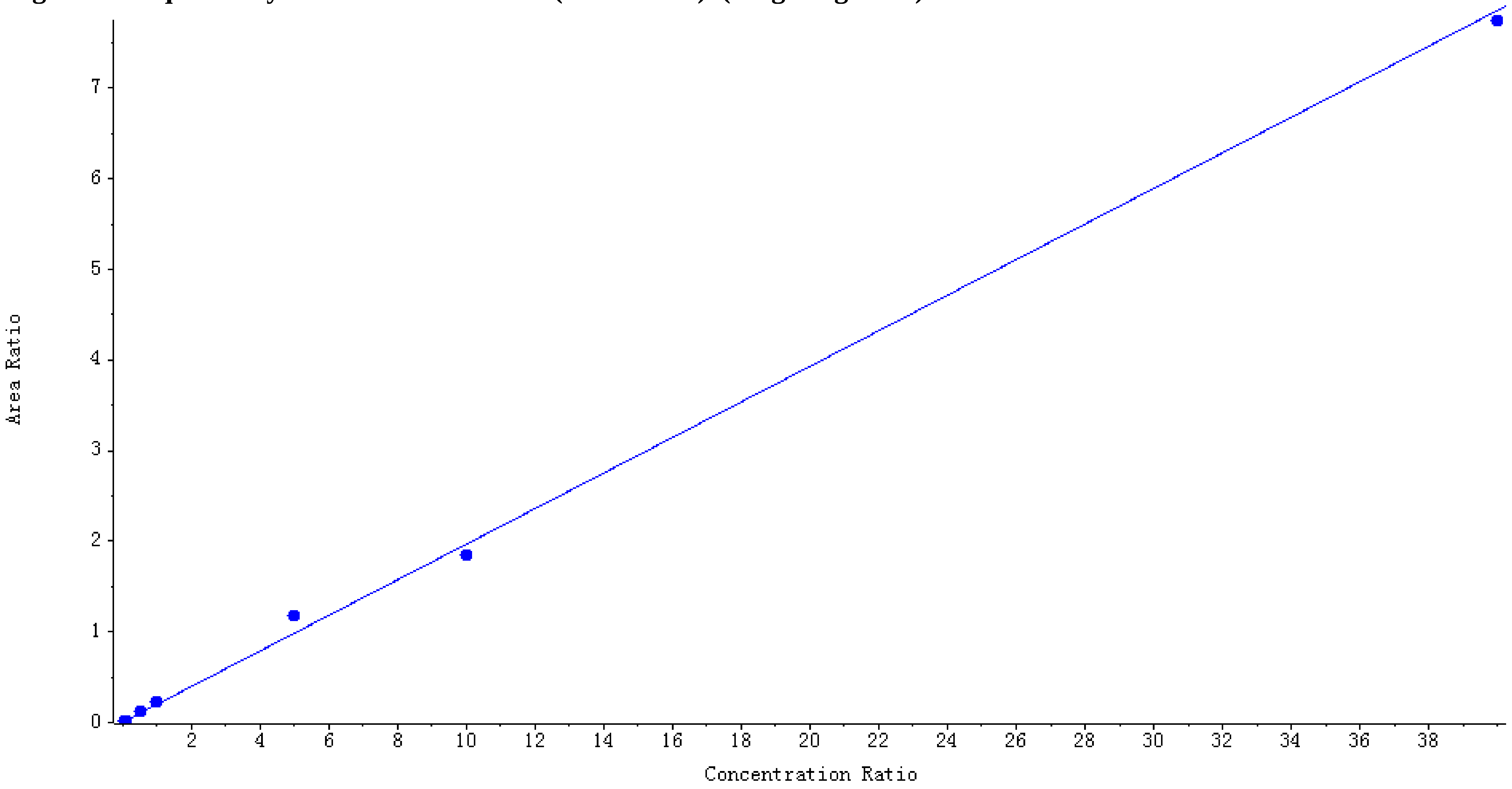

Peak Review

BLANK
zea(C14:0/C16:0) AREA:N/A
S/N:N/A

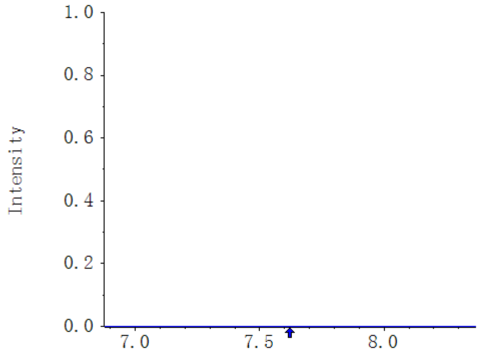

MWMS_20200904_1
zea(C14:0/C16:0) AREA:N/A
S/N:N/A

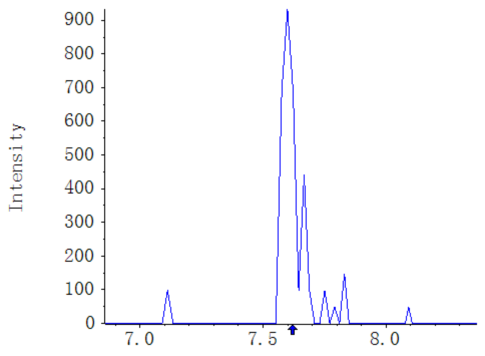

A20024797a_a
zea(C14:0/C16:0) AREA:N/A
S/N:N/A

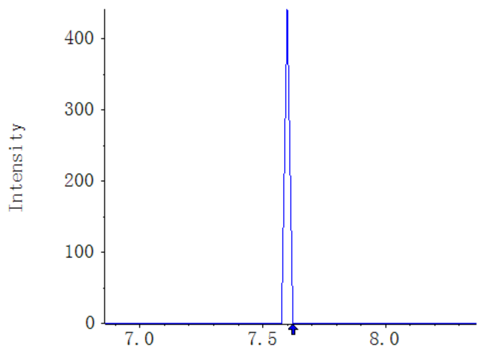

A20024797a_b
zea(C14:0/C16:0) AREA:N/A
S/N:N/A

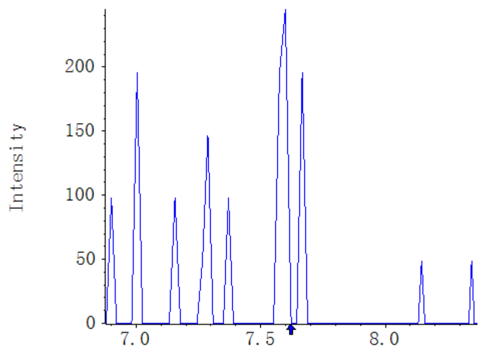

A20024800a_a
zea(C14:0/C16:0) AREA:1.668e5
S/N:165.0

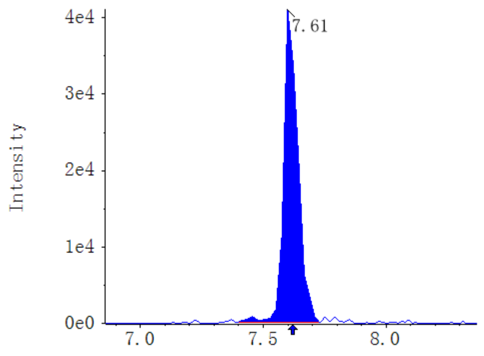

A20024800a_b
zea(C14:0/C16:0) AREA:1.500e5
S/N:219.2

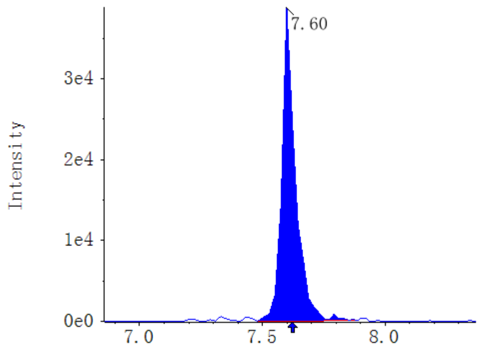

A20024802a_a
zea(C14:0/C16:0) AREA:9.076e4
S/N:92.4

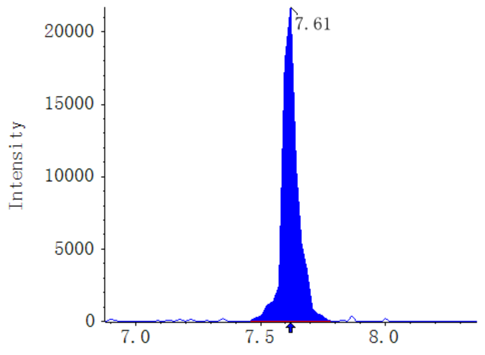

A20024802a_b
zea(C14:0/C16:0) AREA:9.226e4
S/N:189.8

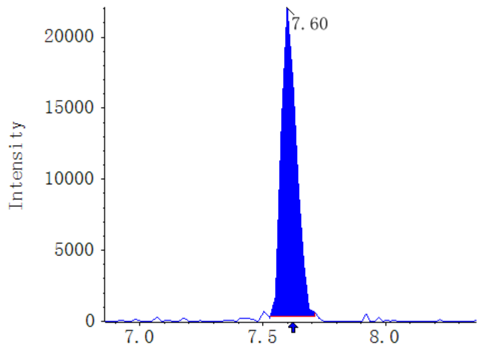

A20024805a_a
zea(C14:0/C16:0) AREA:N/A
S/N:N/A

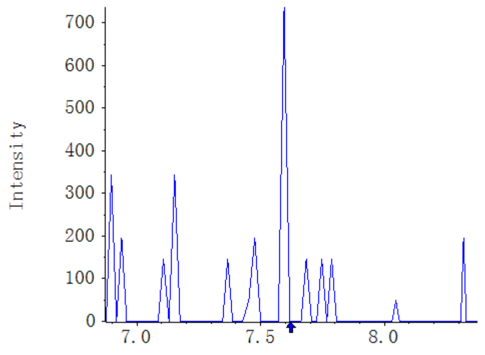

A20024805a_b
zea(C14:0/C16:0) AREA:N/A
S/N:N/A

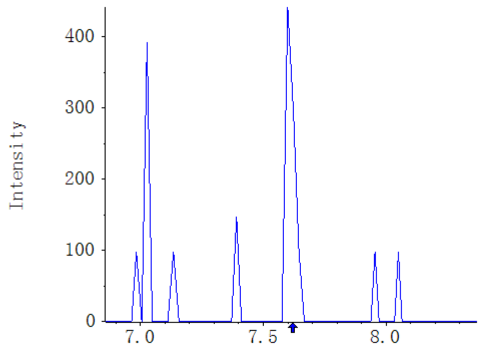

A20024808a_a
zea(C14:0/C16:0) AREA:6.899e4
S/N:119.1

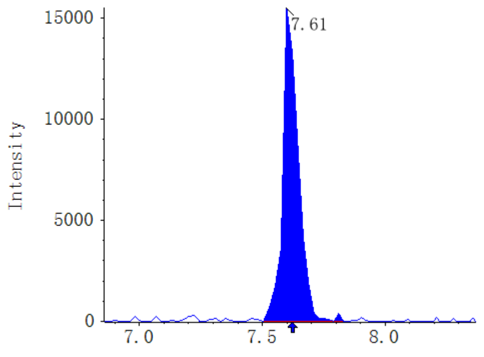

A20024808a_b
zea(C14:0/C16:0) AREA:6.171e4
S/N:158.9

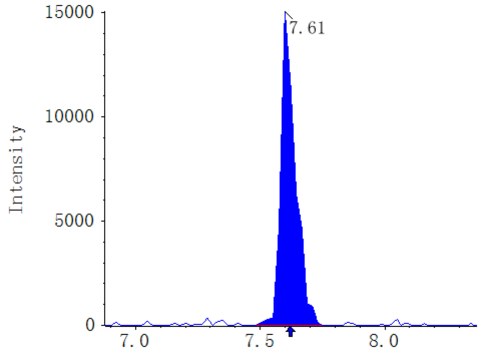

A20024811a_a
zea(C14:0/C16:0) AREA:6.485e4
S/N:237.8

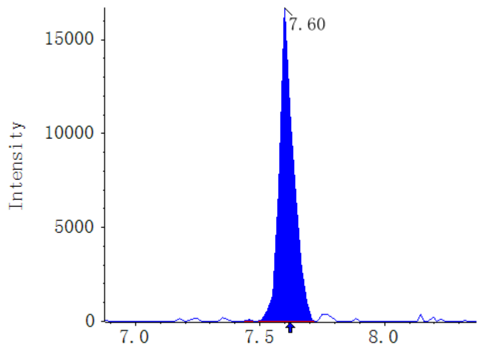

A20024811a_b
zea(C14:0/C16:0) AREA:7.592e4
S/N:116.2

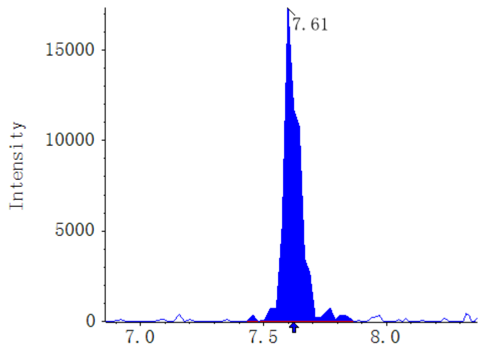

Compound name: zeaxanthin dipalmitate
Regression Equation: y = 0.45801 x + 0.00456 (r = 0.99343) (weighting: 1 / x)

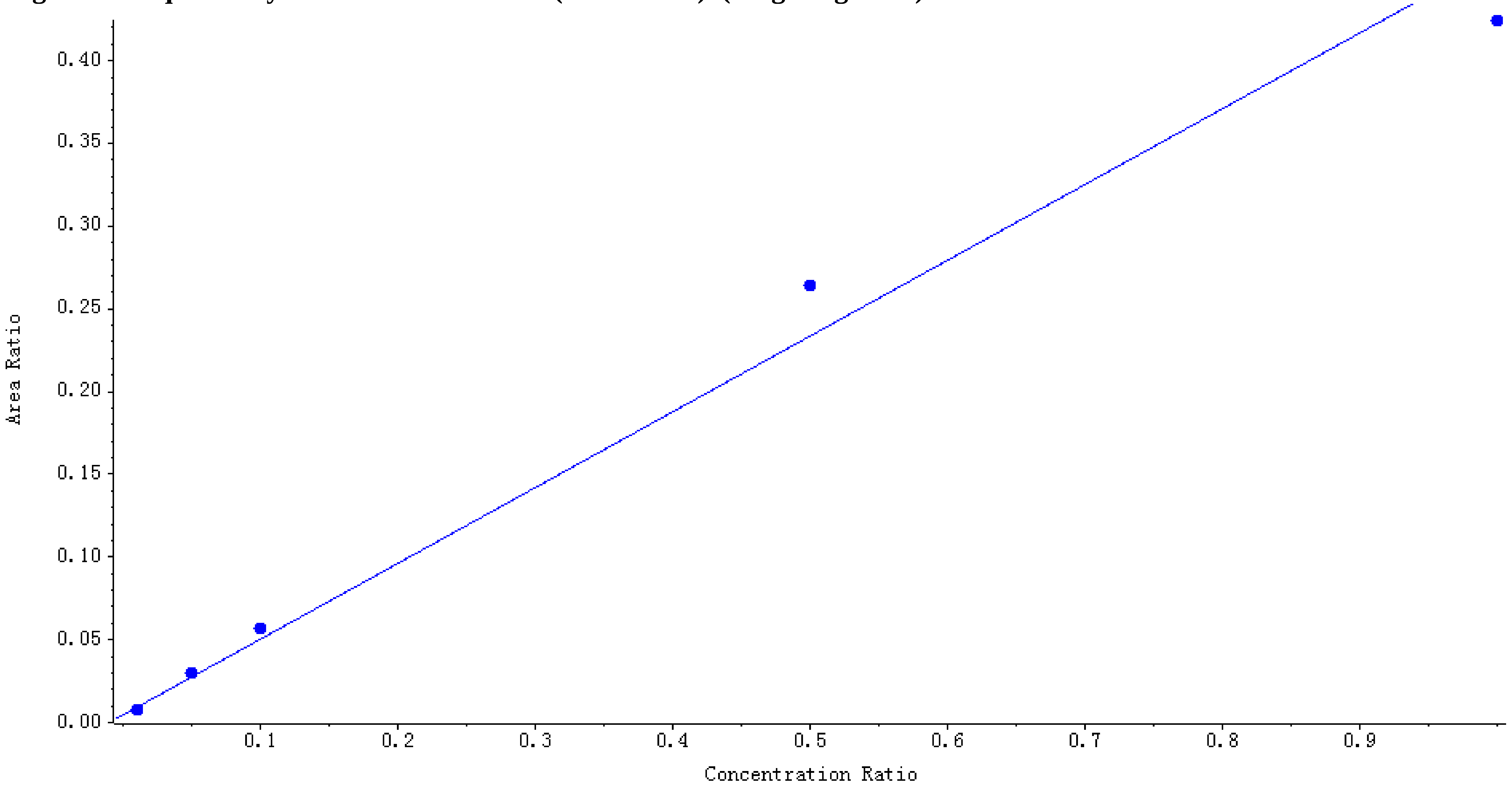

Peak Review

BLANK
zea(C16:0/C16:0)-2 AREA:N/A
S/N:N/A

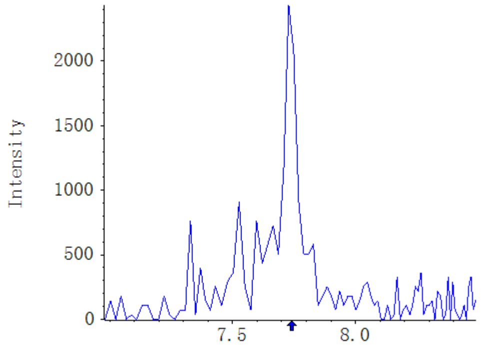

MWMS_20200904_1
zea(C16:0/C16:0)-2 AREA:3.074e5
S/N:315.8

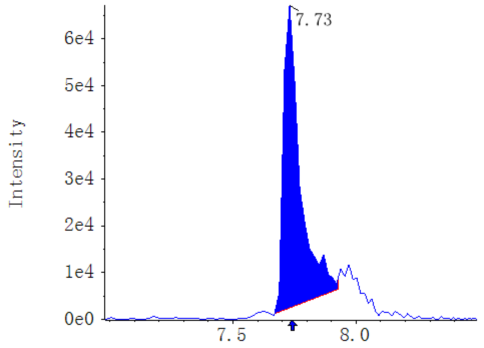

A20024797a_a
zea(C16:0/C16:0)-2 AREA:1.314e4
S/N:17.9

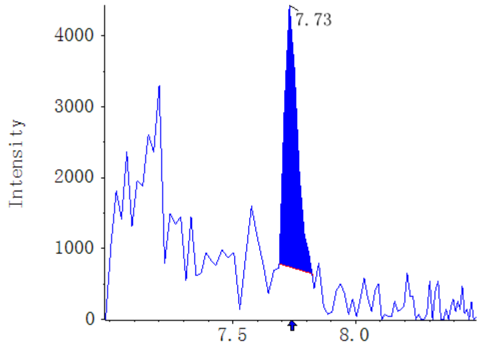

A20024797a_b
zea(C16:0/C16:0)-2 AREA:9.233e3
S/N:10.3

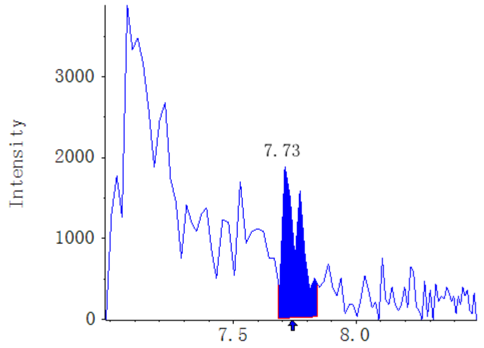

A20024800a_a
zea(C16:0/C16:0)-2 AREA:6.412e6
S/N:230.5

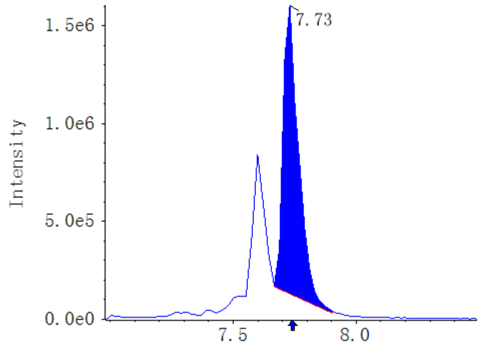

A20024800a_b
zea(C16:0/C16:0)-2 AREA:5.319e6
S/N:261.0

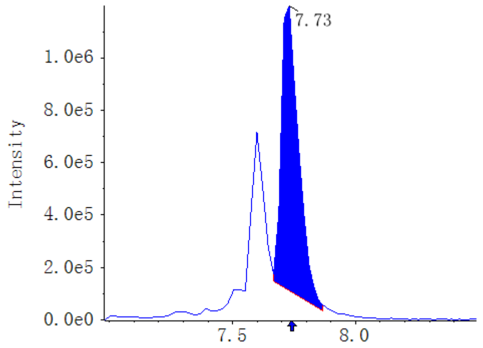

A20024802a_a
zea(C16:0/C16:0)-2 AREA:6.838e6
S/N:256.3

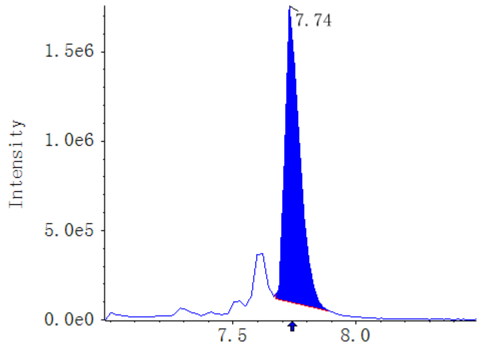

A20024802a_b
zea(C16:0/C16:0)-2 AREA:5.272e6
S/N:278.2

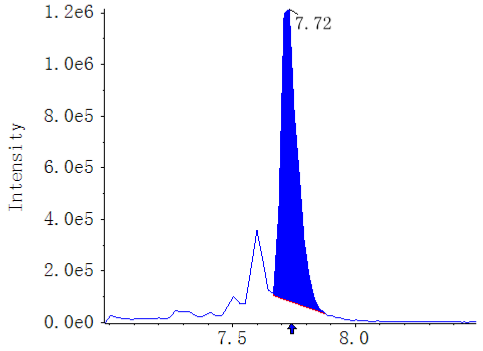

A20024805a_a
zea(C16:0/C16:0)-2 AREA:4.314e4
S/N:35.0

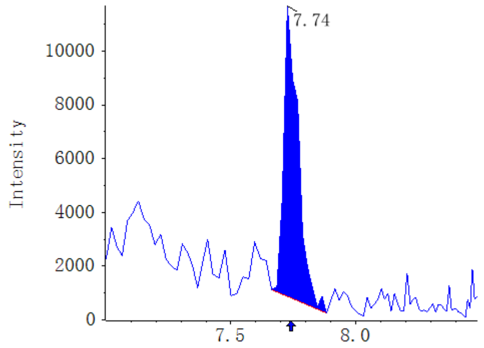

A20024805a_b
zea(C16:0/C16:0)-2 AREA:3.763e4
S/N:41.0

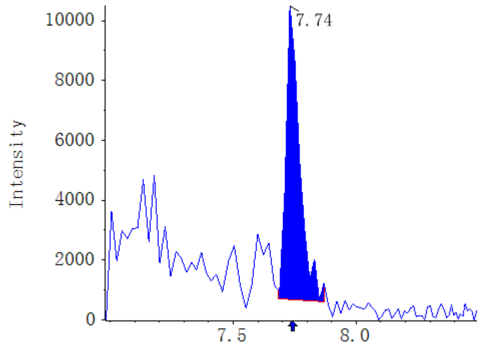

A20024808a_a
zea(C16:0/C16:0)-2 AREA:4.679e6
S/N:258.1

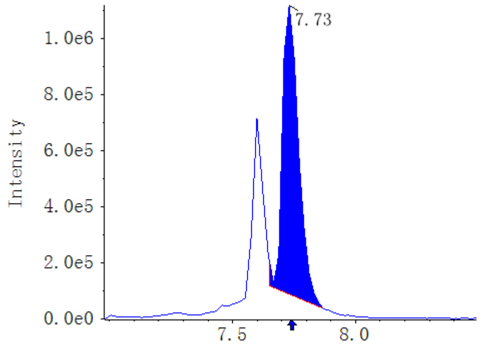

A20024808a_b
zea(C16:0/C16:0)-2 AREA:4.487e6
S/N:203.8

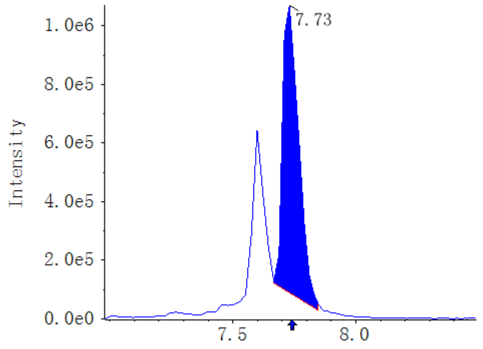

A20024811a_a
zea(C16:0/C16:0)-2 AREA:5.478e6
S/N:252.4

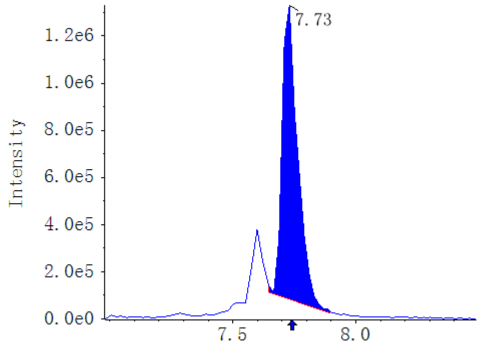

A20024811a_b
zea(C16:0/C16:0)-2 AREA:6.471e6
S/N:236.9

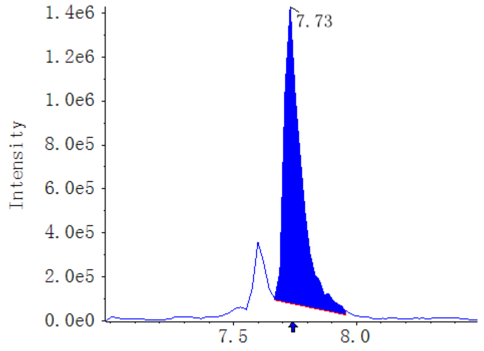

Compound name: zeaxanthin-palmitate-stearate
Regression Equation: y = 0.19627 x + 0.00647 (r = 0.99741) (weighting: 1 / x)

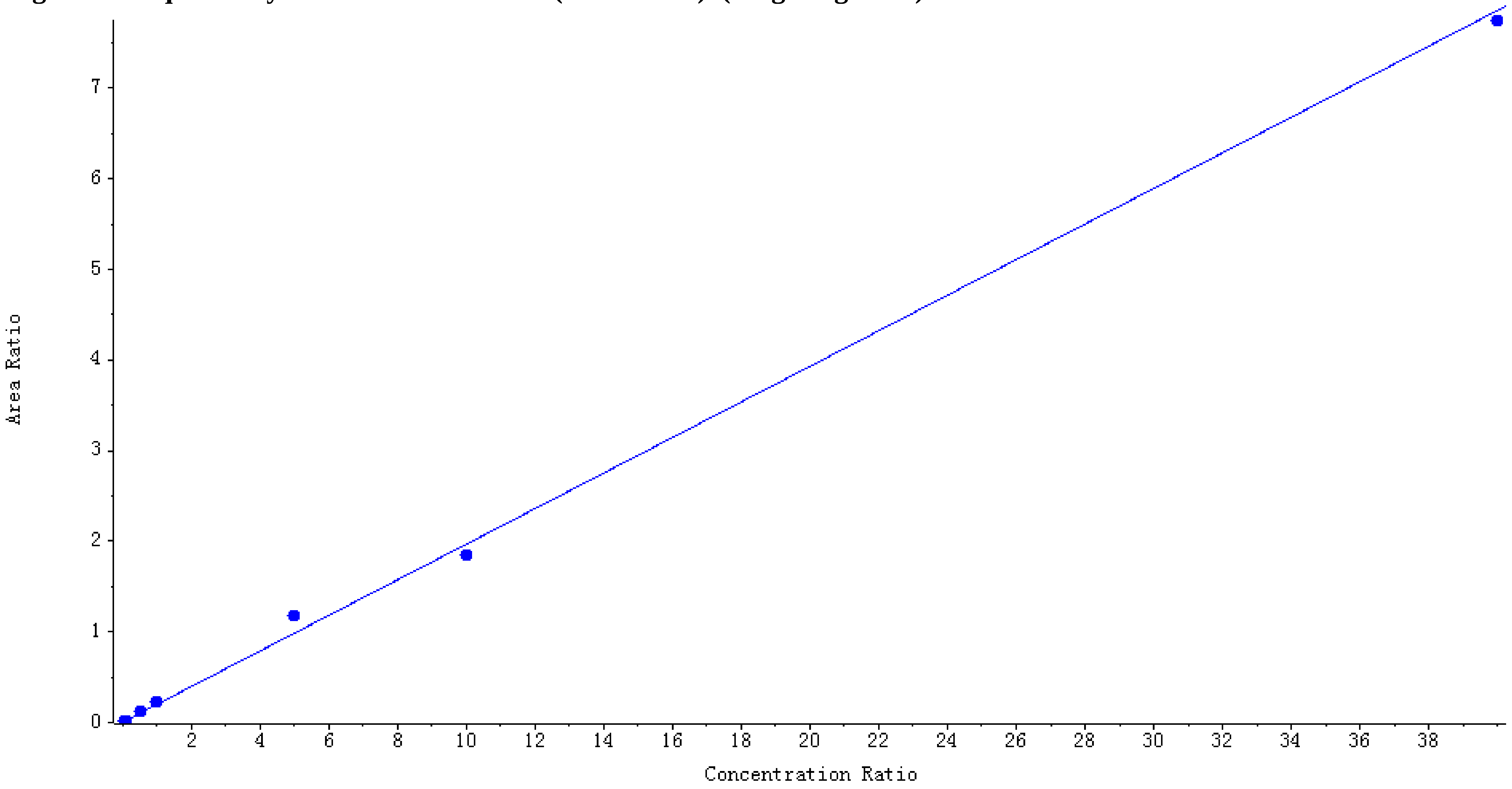

Peak Review

BLANK
zea(C16:0/C18:0) AREA:N/A
S/N:N/A

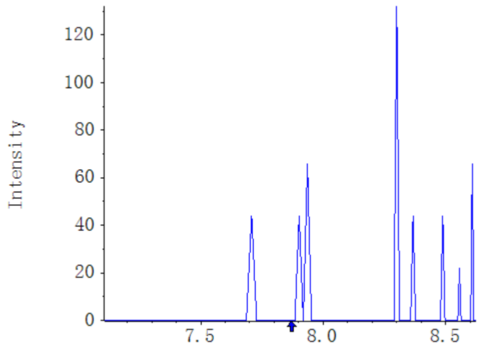

MWMS_20200904_1
zea(C16:0/C18:0) AREA:N/A
S/N:N/A

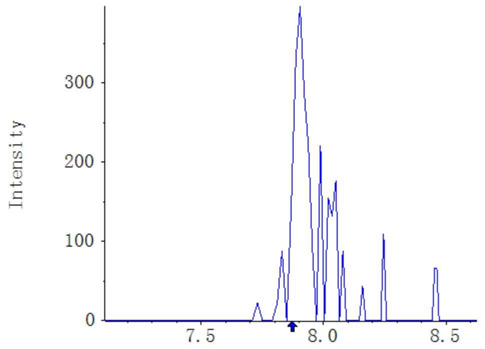

A20024797a_a
zea(C16:0/C18:0) AREA:N/A
S/N:N/A

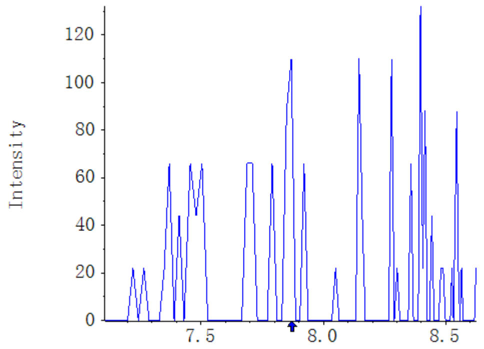

A20024797a_b
zea(C16:0/C18:0) AREA:N/A
S/N:N/A

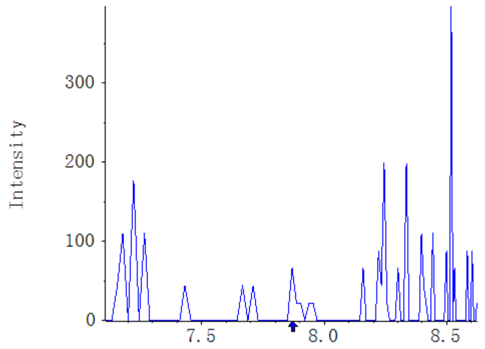

A20024800a_a
zea(C16:0/C18:0) AREA:6.107e4
S/N:163.2

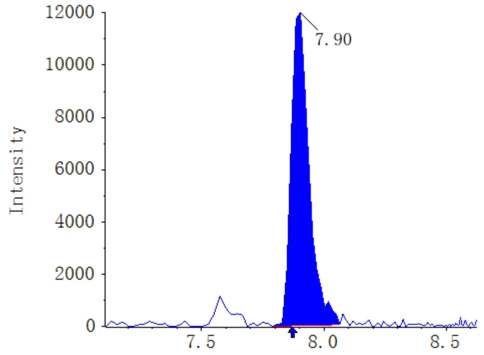

A20024800a_b
zea(C16:0/C18:0) AREA:4.311e4
S/N:186.0

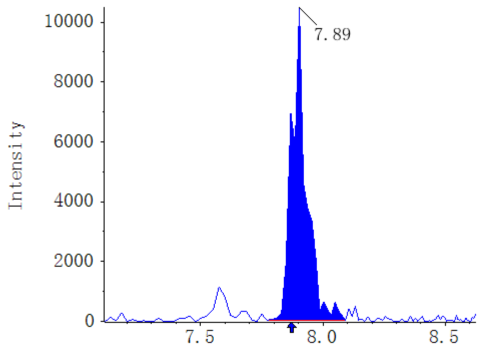

A20024802a_a
zea(C16:0/C18:0) AREA:4.896e4
S/N:190.0

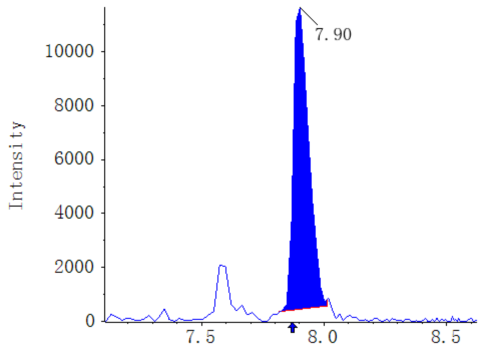

A20024802a_b
zea(C16:0/C18:0) AREA:3.506e4
S/N:131.9

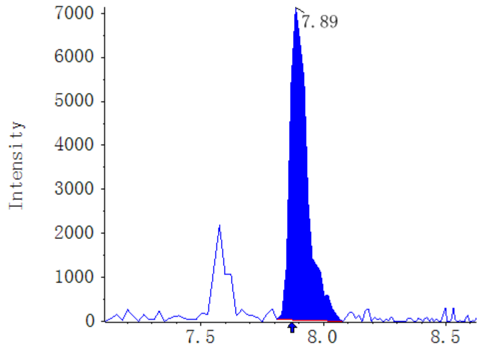

A20024805a_a
zea(C16:0/C18:0) AREA:N/A
S/N:N/A

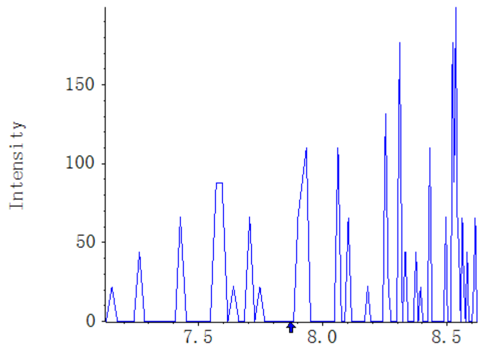

A20024805a_b
zea(C16:0/C18:0) AREA:N/A
S/N:N/A

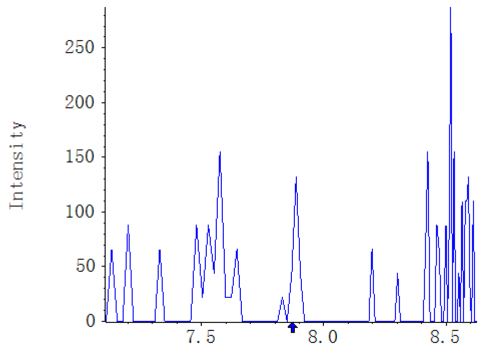

A20024808a_a
zea(C16:0/C18:0) AREA:4.360e4
S/N:208.5

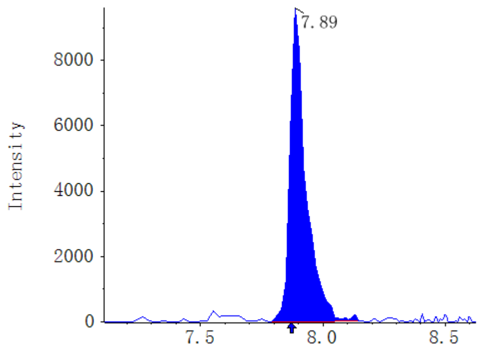

A20024808a_b
zea(C16:0/C18:0) AREA:3.772e4
S/N:214.9

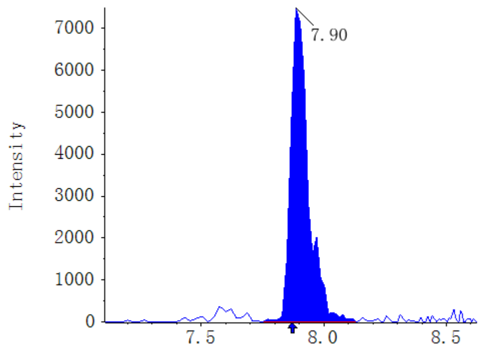

A20024811a_a
zea(C16:0/C18:0) AREA:2.617e4
S/N:84.3

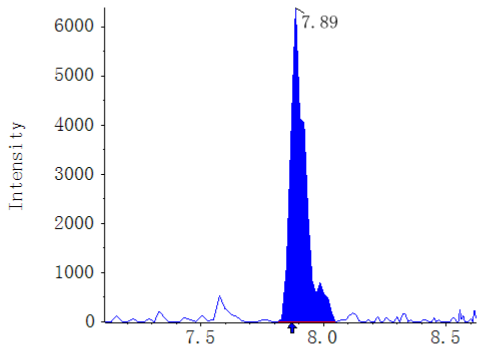

A20024811a_b
zea(C16:0/C18:0) AREA:2.908e4
S/N:173.2

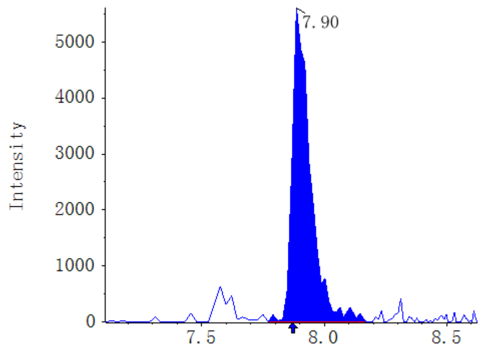

Compound name: zeaxanthin-oleate-palmitate
Regression Equation: y = 0.19627 x + 0.00647 (r = 0.99741) (weighting: 1 / x)

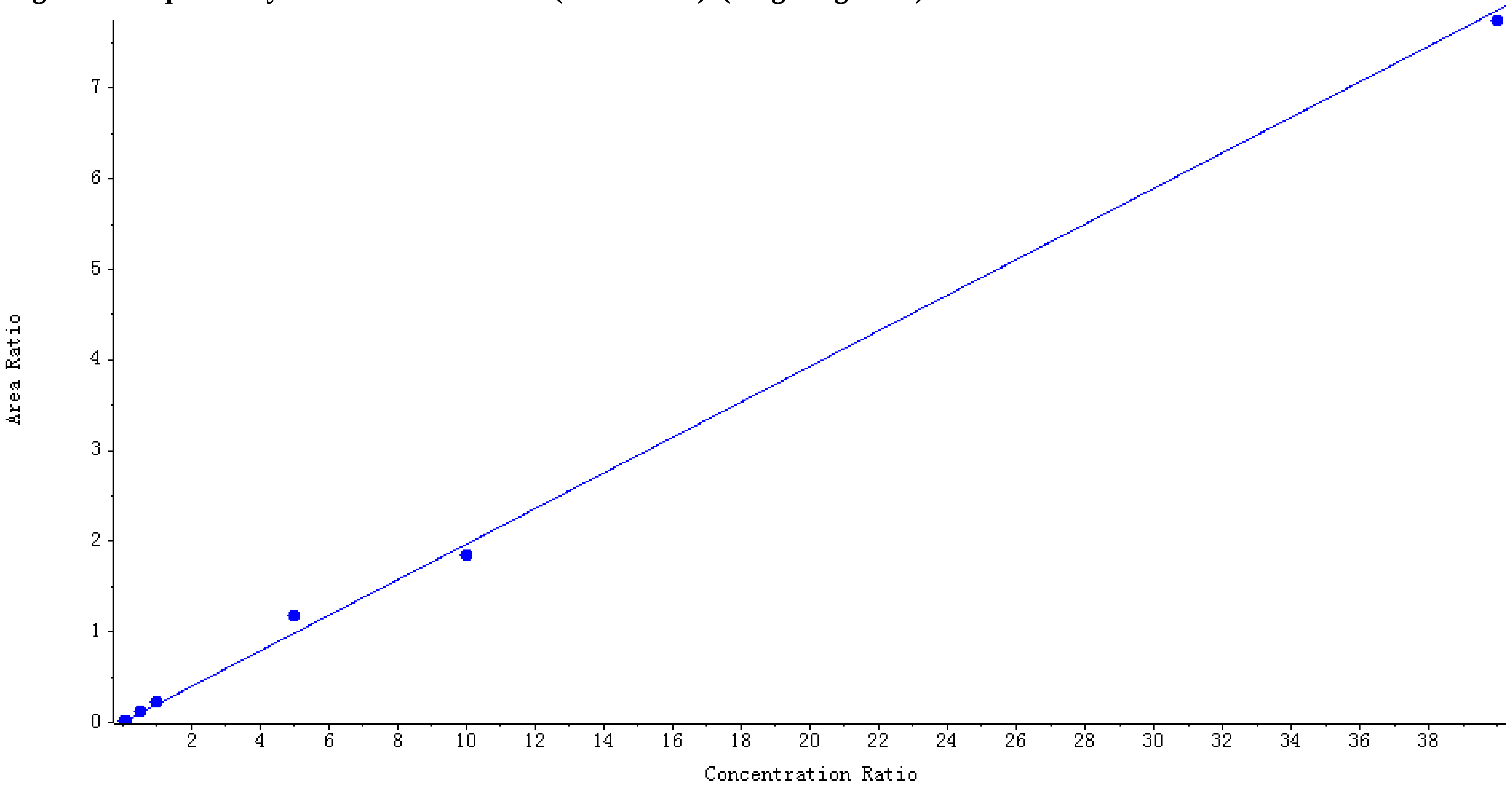

Peak Review

BLANK
zea(C18:1/C16:0) AREA:N/A
S/N:N/A

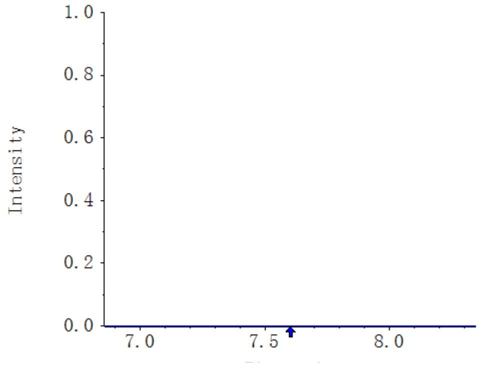

MWMS_20200904_1
zea(C18:1/C16:0) AREA:N/A
S/N:N/A

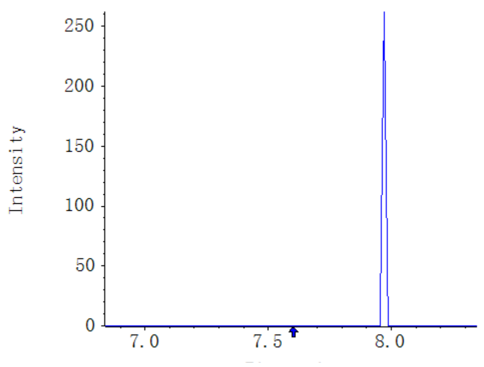

A20024797a_a
zea(C18:1/C16:0) AREA:N/A
S/N:N/A

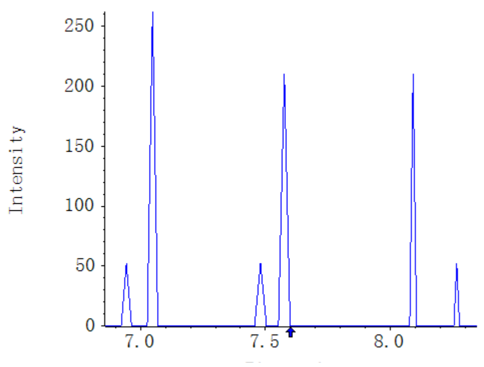

A20024797a_b
zea(C18:1/C16:0) AREA:N/A
S/N:N/A

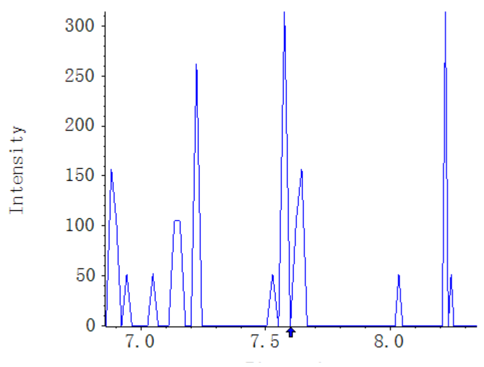

A20024800a_a
zea(C18:1/C16:0) AREA:1.765e5
S/N:181.7

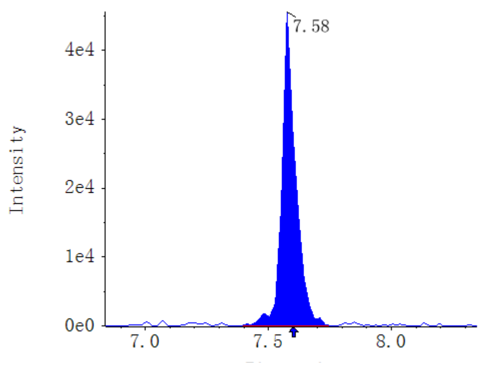

A20024800a_b
zea(C18:1/C16:0) AREA:1.755e5
S/N:244.7

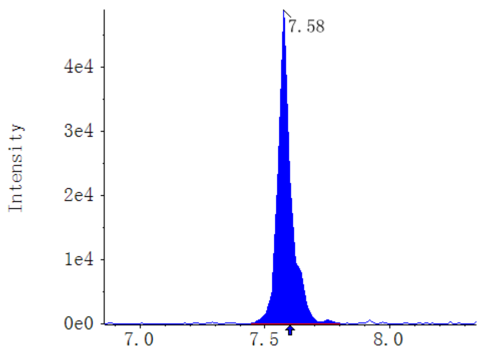

A20024802a_a
zea(C18:1/C16:0) AREA:2.843e5
S/N:493.9

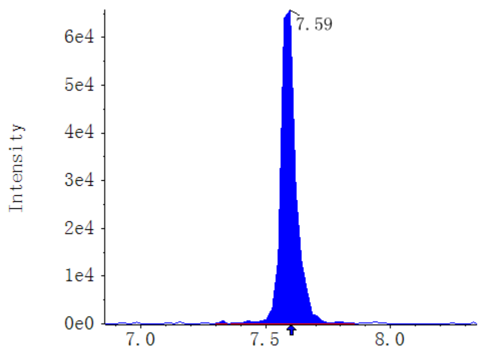

A20024802a_b
zea(C18:1/C16:0) AREA:2.587e5
S/N:225.9

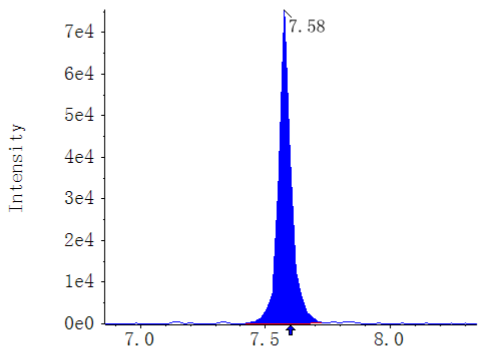

A20024805a_a
zea(C18:1/C16:0) AREA:N/A
S/N:N/A

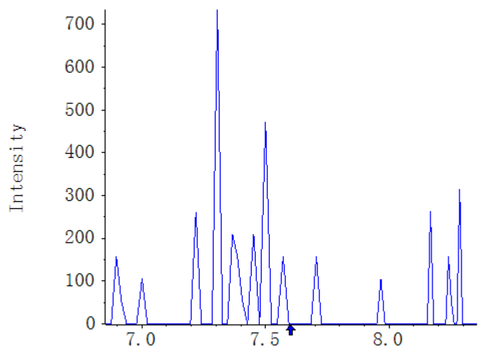

A20024805a_b
zea(C18:1/C16:0) AREA:N/A
S/N:N/A

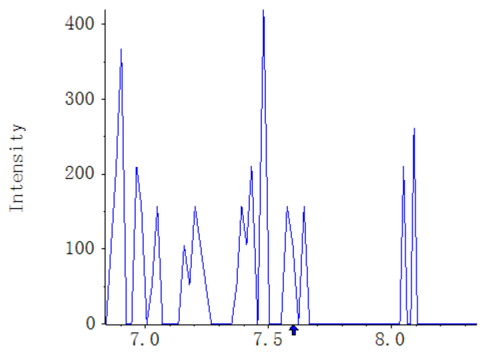

A20024808a_a
zea(C18:1/C16:0) AREA:4.207e4
S/N:103.3

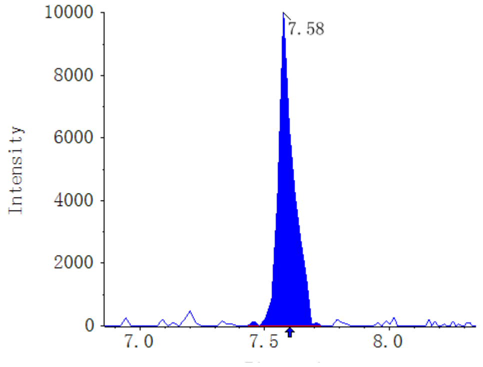

A20024808a_b
zea(C18:1/C16:0) AREA:4.599e4
S/N:89.1

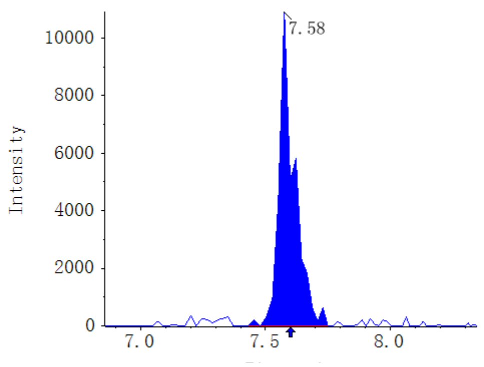

A20024811a_a
zea(C18:1/C16:0) AREA:7.804e4
S/N:228.4

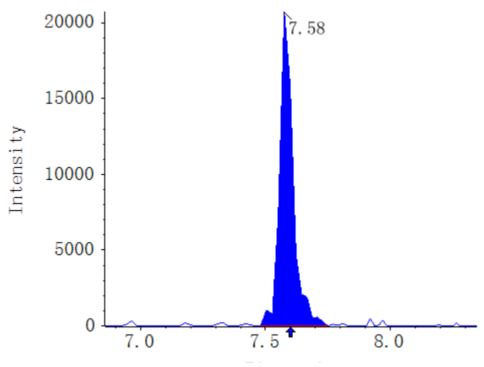

A20024811a_b
zea(C18:1/C16:0) AREA:7.848e4
S/N:124.4

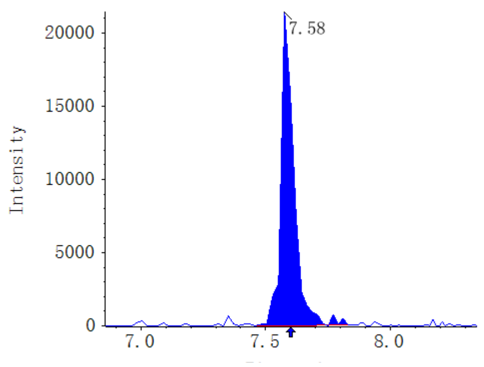

Compound name: β-cryptoxanthin palmitate
Regression Equation: y = 0.34449 x + 0.00821 (r = 0.99717) (weighting: 1 / x)

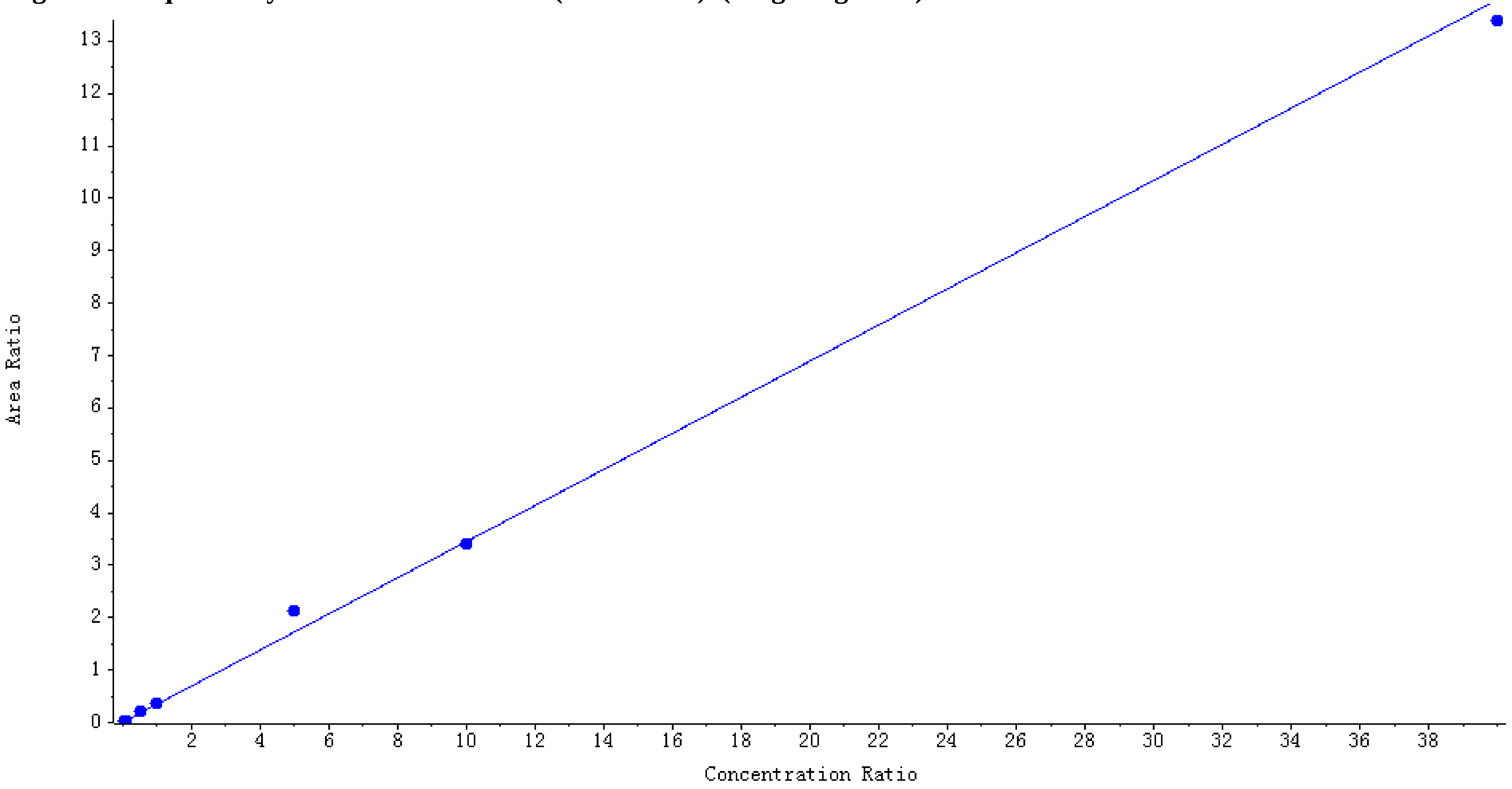

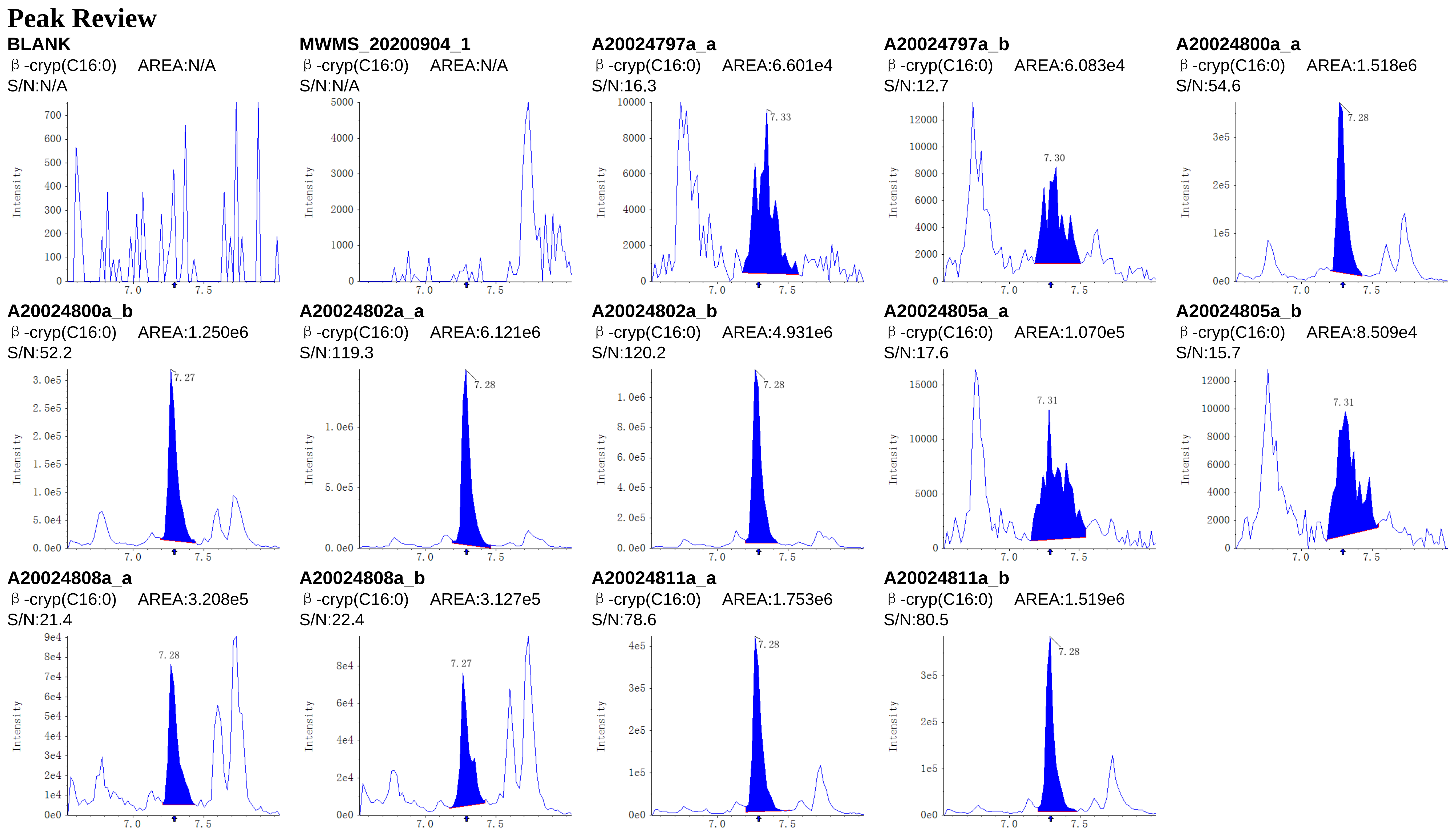

Figure S1. The carotenoid standards and the raw data for quantification including calibration curves.
