# Supplementary material for: Transcriptomics and Metabolomics Reveal the Critical Genes of Carotenoid Biosynthesis and Color Formation of Goji (Lycium barbarum L.) Fruit Ripening
Source: Plants (Basel). 2023 Jul 27;12(15):2791. doi: 10.3390/plants12152791 (PMC10421014; doi:10.3390/plants12152791)
Supplement: Supplementary file 1 [file plants-12-02791-s001.zip › Figures S2 and S3.pdf]

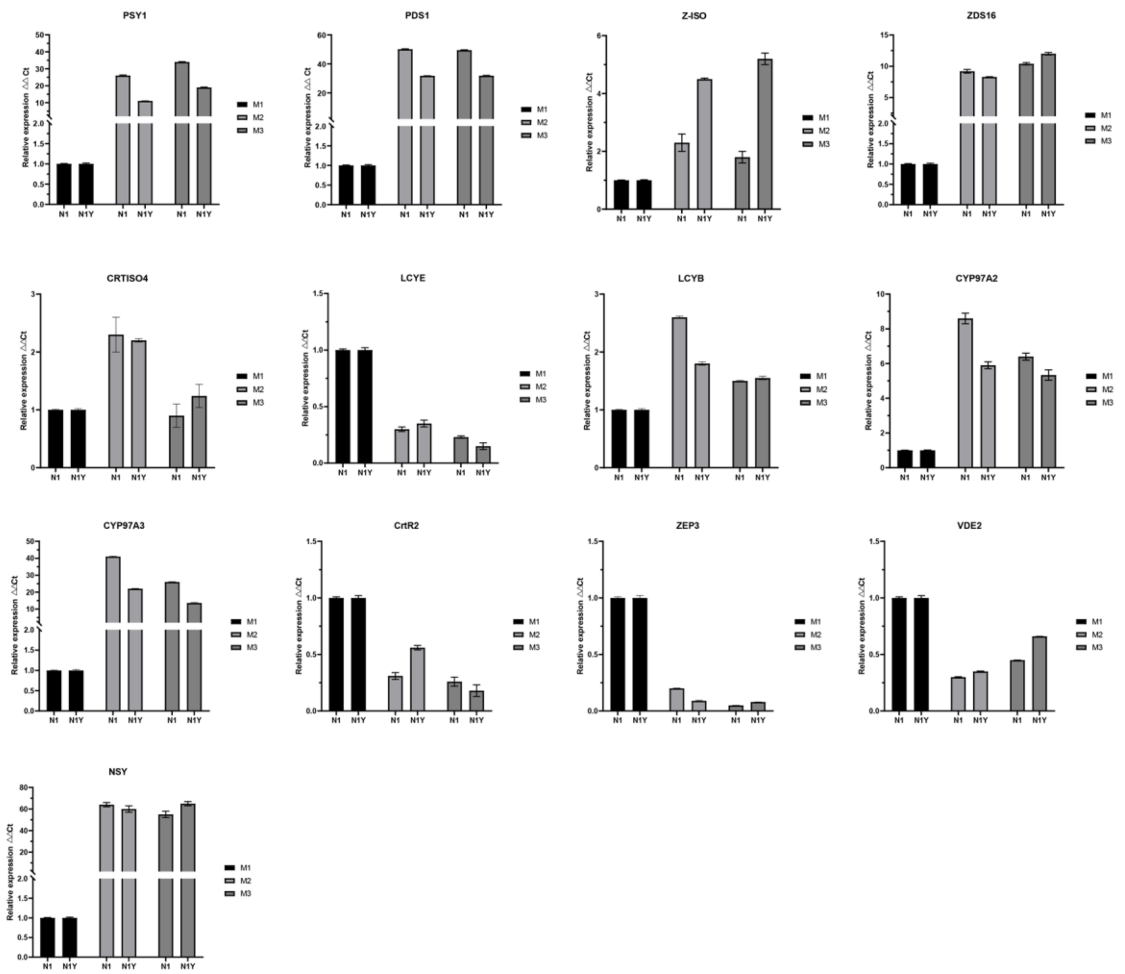

Figure S2. q-PCR validation of RNA-seq data

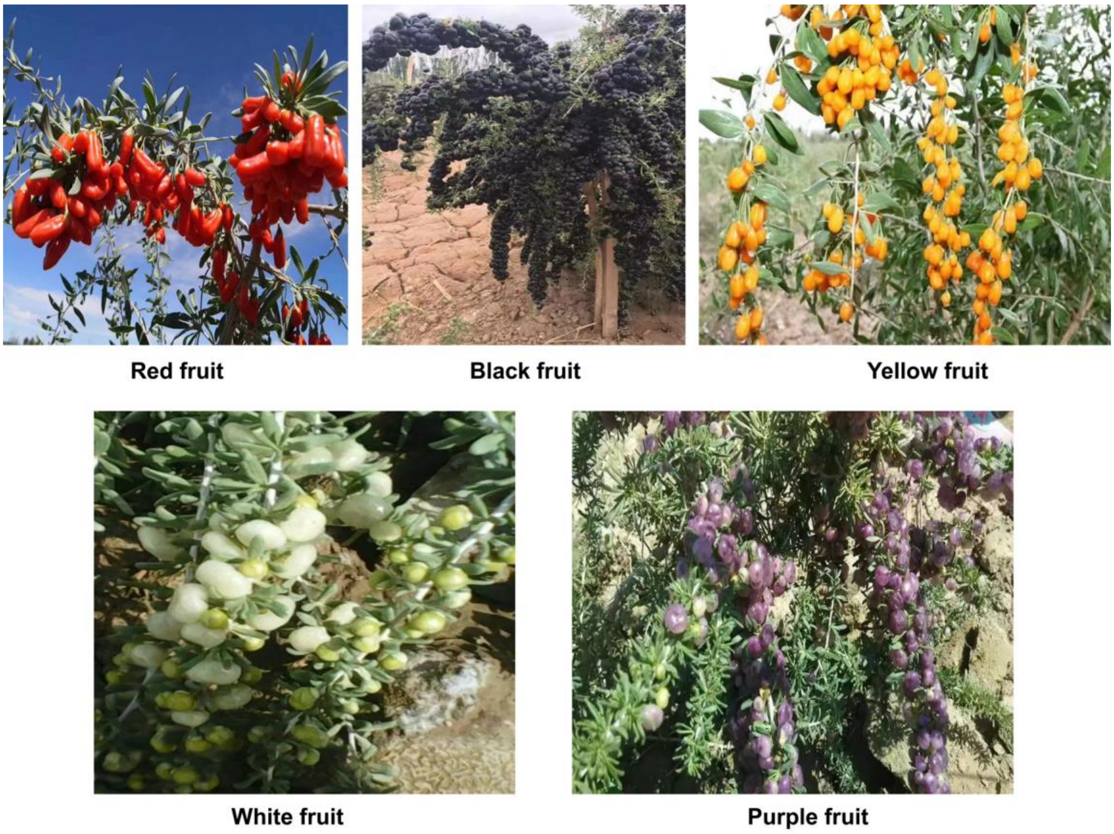

Figure S3. Fruit color of different wolfberry varieties
